# The oldest *Homo erectus* buried lithic horizon from the Eastern Saharan Africa. EDAR 7 - an Acheulean assemblage with Kombewa method from the Eastern Desert, Sudan

**DOI:** 10.1371/journal.pone.0248279

**Published:** 2021-03-23

**Authors:** Mirosław Masojć, Ju Yong Kim, Joanna Krupa-Kurzynowska, Young Kwan Sohn, Maciej Ehlert, Grzegorz Michalec, Marzena Cendrowska, Eric Andrieux, Simon J. Armitage, Marcin Szmit, Ewa Dreczko, Jin Cheul Kim, Ji Sung Kim, Gwang-Soo Lee, Piotr Moska, Modather Abdalla Jadain

**Affiliations:** 1 Institute of Archaeology, University of Wrocław, Wrocław, Poland; 2 Korea Institute of Geoscience and Mineral Resources (KIGAM), Daejeon, Republic of Korea; 3 Faculty of Geoengineering, Mining and Geology, Wroclaw University of Science and Technology, Wrocław, Poland; 4 Department of Geology and Research Institute of Natural Science, Gyeongsang National University (GNU), Jinju, Republic of Korea; 5 Archeolodzy.org Foundation, Wrocław, Poland; 6 Department of Geography, Royal Holloway, University of London, London, United Kingdom; 7 Department of Archaeology, Durham University, United Kingdom; 8 SFF Centre for Early Sapiens Behaviour (SapienCE), University of Bergen, Bergen, Norway; 9 Gdańsk Archaeological Museum, Gdańsk, Poland; 10 Institute of Physics, Division of Geochronology and Isotope Research of the Environmental, Silesian University of Technology, Gliwice, Poland; 11 Department of Archaeology, Al Neelain University, Khartoum, Sudan; University at Buffalo - The State University of New York, UNITED STATES

## Abstract

Although essential for reconstructing hominin behaviour during the Early Palaeolithic, only a handful of Acheulean sites have been dated in the Eastern Sahara region. This is due to the scarcity of sites for this time period and the lack of datable material. However, recent excavations in the Atbara region (Sudan) have provided unique opportunities to analyse and date Acheulean stone tools. We report here on EDAR 7, part of a cluster of Acheulean and Middle Stone Age (MSA) sites that were recently discovered in the Eastern Desert Atbara River (EDAR) region, located in the Eastern Desert (Sudan) far from the Nile valley. At EDAR 7, a 3.5 metre sedimentary sequence was excavated, allowing an Acheulean assemblage to be investigated using a combination of sedimentology, stone tool studies and optically stimulated luminescence dating (OSL). The site has delivered a complete Acheulean knapping *chaine opératoire*, providing new information about the Saharan Acheulean. The EDAR 7 site is interpreted as a remnant of a campsite based on the co-occurrence of two reduction modes: one geared towards the production of Large Cutting Tools (LCTs), and the other based on the flaking of small debitage and production of flake tools. Particularly notable in the EDAR 7 assemblage is the abundance of cleavers, most of which display evidence of flake production. Implementation of giant Kombewa flakes was also observed. A geometric morphometric analysis of hand-axes was conducted to verify a possible Late Acheulean assemblage standardisation in the Nubian Sahara. In addition, the analysis of micro-traces and wear on the artefacts has provided information on the use history of the Acheulean stone tools. Sediment analyses and OSL dating show that the EDAR 7 sequence contains the oldest Acheulean encampment remains in the Eastern Sahara, dated to the MIS 11 or earlier. This confirms that *Homo erectus* occupied the EDAR region during Middle Pleistocene humid periods, and demonstrates that habitable corridors existed between the Ethiopian Highlands, the Nile and the Red Sea coast, allowing population dispersals across the continent and out of it.

## Introduction

Stratified Palaeolithic sites in the Eastern Sahara are rare [1–4]. Besides the sites situated in the Nile valley [5–8] and Egyptian oases [9–12], cave sites in the Red Sea Mountains [13, 14] and individual open-air sites in the desert [15, 16] have been recorded. The number of dense, homogeneous, and datable Palaeolithic assemblages in this part of Africa is far from satisfactory [17]. While sites representing the Levallois tradition and the Late Palaeolithic of the Nile valley [18–21] are better represented, the oldest cultural episodes, e.g. Oldowan pebble tool tradition are absent or limited to a few sites only [22–25], as is the case of Acheulean Industrial Complex (i.e. Mode 2 of Clark [26]) [5, 10–12, 27–31]. It is in this context that several Palaeolithic sites in the Eastern Desert within an ancient watercourse system, referred to as the Eastern Desert Atbara River (EDAR) sites, were studied and dated, providing a unique opportunity to understand the chronology, stratigraphic positions and cultural properties of these rare sequences [32].

**Fig 1:**
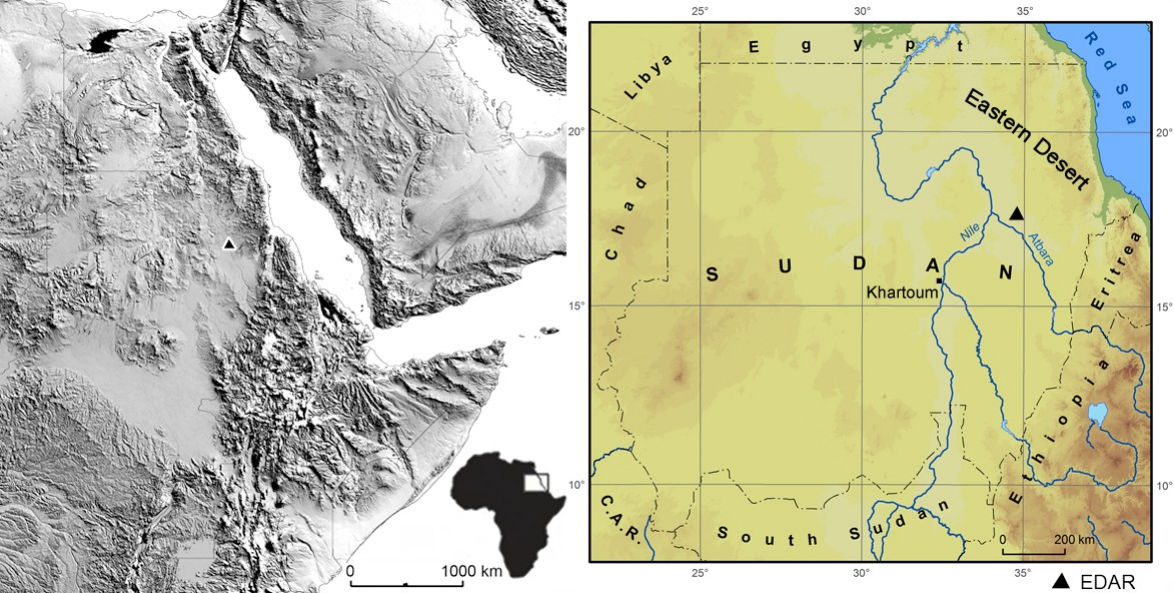
Location of Eastern Desert Atbara River (EDAR) area. Left: NE Africa with the EDAR area marked. Right: map of Sudan with the location of EDAR within the Eastern Desert. Grey-shaded Digital Elevation Model (DEM) derived from Shuttle Radar Topography Mission elevation data (SRTM). The map was prepared from open access Digital Terrain Elevation Data of SRTM Version 3.0 Global 1 arc second dataset (https://doi.org/10.5066/f7pr7tft).

The EDAR study area is located at 17°39’ N, 34°46’ E, in the Eastern Desert of Sudan, and is part of the large Wadi el Arab depression which stretches from the Red Sea Mountains to the Atbara river. EDAR itself is within the Atbara river drainage basin, and lies between the Nile valley to the west and the Atbara valley to the south (about 70 km East from the town of Atbara) (Fig 1). The present landscape of the desert around EDAR is a wide flat plain. Gold mining shafts located there have revealed a complex of Palaeolithic sites, in which both Acheulean and MSA sites have been recognised in their original stratigraphic contexts.

**Fig 2:**
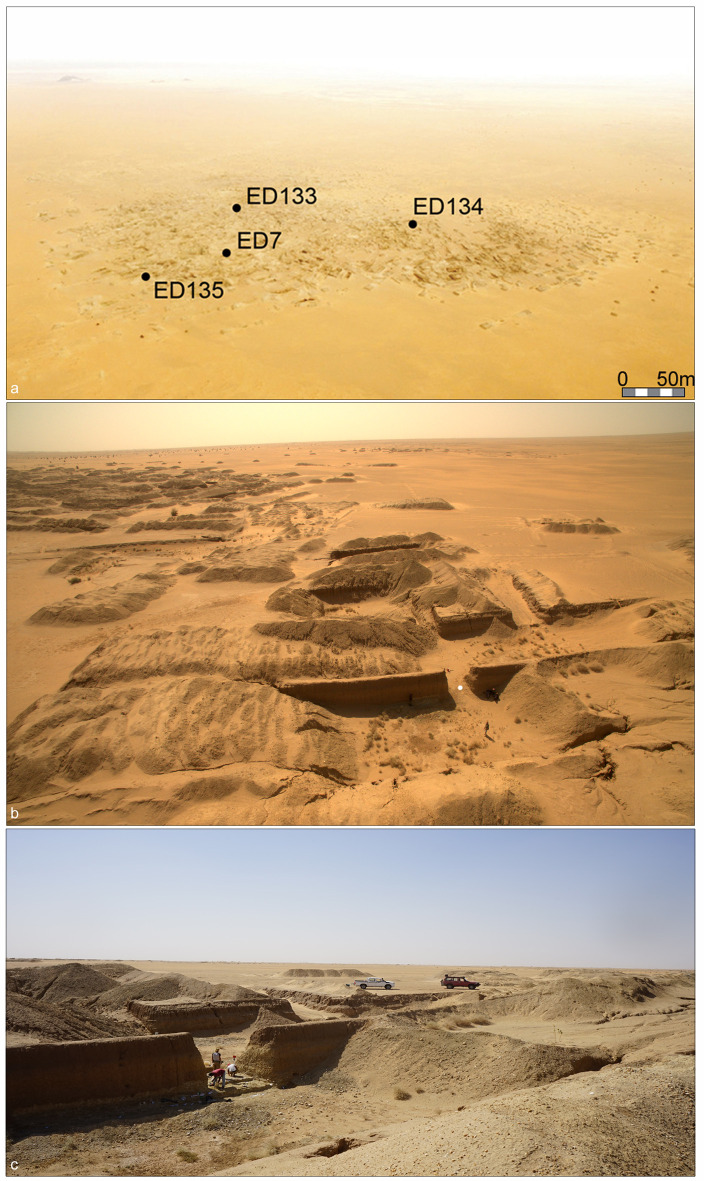
EDAR area. (a) Aerial photograph of the gold mining area annotated to show the location of the main EDAR sites; (b-c) Location (white dot) of the excavation area (3m x 3m) at EDAR 7.

**Fig 3:**
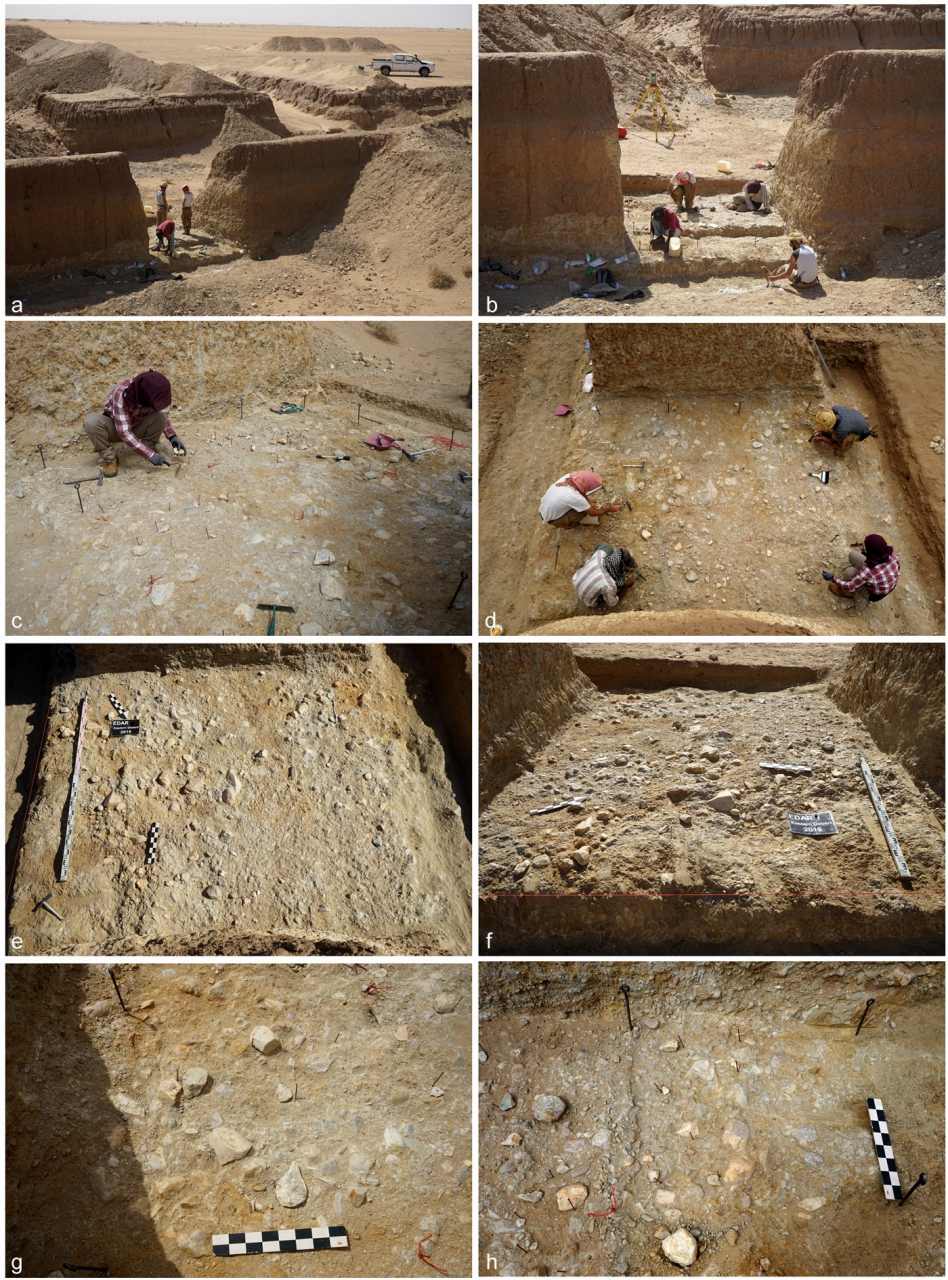
EDAR 7. (a-d) Undisturbed area (3m x 3m) within the mine being excavated; (e-f) Top of the Acheulean horizon; (g) Quartzite hand-axes among the debitage; (h) Quartzite cores and the debitage.

Preliminary examination of the EDAR sites revealed a number of different Acheulean assemblages, including artefacts from the latest African Acheulean, within Middle Pleistocene fluvial braided river channel sediments [32]. This article presents the Acheulean assemblage from the EDAR 7 site, which was excavated in 2019. Discovered at the depth of 3 m below the present-day land surface, it is one of the few dense Acheulean inventories in the Eastern Sahara. EDAR 7 was initially discovered in 2014, when stone artefacts were recognised in the walls of mineshafts (Fig 2A). The area, which has been considerably damaged by recent earthworks, yielded several disconnected profiles containing discrete Acheulean and MSA artefacts (Fig 2B). After several seasons of investigating the site, an intact Acheulean stone assemblage was discovered in one of the mine passages (Figs 2C and 3).

## Materials and methods

All necessary permits were obtained for the described study, which complied with all relevant regulations. Permission was issued by the Director–General of the Sudanese National Corporation for Antiquities and Museums (NCAM). The research was carried out by the Institute of Archaeology, University of Wrocław, Poland together with the Faculty of Archaeology & Tourism, Al Neelain University, (Khartoum), Sudan, the Korea Institute of Geoscience and Mineral Resources (KIGAM), the Research Institute of Natural Science of the Gyeonsang National University, Republic of Korea and Royal Holloway University of London, United Kingdom. During the period of the project, the archaeological artefacts were stored at the Al Neelain University. After completion of the field work all materials were stored in the National Museum in Khartoum.

### Analysis of the lithic inventory and its stratigraphic context

Excavations at EDAR 7 covered an area of 9 m² (Fig 3). The taxonomic affiliation of archaeological horizons was based on the presence of characteristic lithic artefacts: hand-axes, cleavers or other Large Cutting Tools (LCTs). A Microsoft Access database was designed to record the entire lithic inventory. Each artefact was given a separate inventory number, and location within a metre grid was recorded. All artefacts larger than 15 mm (those being smaller or equal to 15 mm were only classified by raw material) were measured, weighed and classified typologically and technologically. In addition, features such as physical state, completeness, heat treatment together with detailed characterisation and measurements for LCTs, cores, debitage, flake tools were recorded. Some of the recorded information is presented in this paper, and the complete database is available as a S1 File.

Spatial analyses, based on data from artefact plotting, were conducted using ArcGis (Desktop 10.6). Distribution and density maps were created for the entire assemblage, as well as for five morphological groups: cores, retouched tools, bifaces, pebble tools and others. Density maps were calculated and plotted using the Kernel Density tool, from ArcGis Spatial Analyst Toolbox.

Stratigraphic sections were exposed above the weathered rhyolite bedrock, where several Acheulean hand-axes were observed in the trench walls. The mineral composition of rocks used as raw material for the production of artefacts was determined using X-ray powder diffraction analysis (XRD). X-ray diffraction measurements were obtained with a Siemens D-5005 diffractometer. Geological samples were collected from stratigraphic trenches and the associated archaeological excavations. Sedimentological data were collected following facies analysis and included observations such as the thickness of units, dominant colour, cementation, presence of carbonate, bedding, structures and artefact content.

### Large cutting tools—geometric morphometric analysis

The assemblage of 23 LCT’s was subject to a geometric morphometric analysis to compare and verify the presence of visible standardisation in Late Acheulean assemblages from the Eastern Sahara region [33–38]. We analysed only those forms of bifacial tools which are entirely preserved or have slight breaks on the distal side. Due to the problem of defining and distinguishing different forms of LCT’s, as well as the problem of the interpretation of morphological variability of products presented in the discussion, all three types of bifacial tools have been included in the analysis–hand-axes, “cleaver-like” hand-axes and cleavers [39]. This approach in the geometric morphometric analysis which includes hand-axes and cleavers was applied in the study of Acheulean assemblages from Western Europe [39].

The sites selected for comparison are situated in Sudan (EDAR 7, EDAR 133 [38]) and Egypt (Dakhla Oasis, site E-72-1 [11], Kharga Oasis 10 [10], Bir Sahara 14 (BS-14) [12]). For most sites the randomly selected samples are of the same size (n = 23), but in the case of BS-14 the sample size was limited to 18 specimens. Every individual number of the artefact was entered to MS Office Excel spreadsheet (separately for each site) and the sample was selected using random sample formula. The artefacts subject to the analysis were made from various types of raw material of local origin (Table 1).

**Table 1:** Sample of hand-axes selected for analysis.

Site	n	Raw material
**EDAR 7**	23	rhyolite and quartzite
**EDAR 133**	23	rhyolite and quartzite
**Kharga Oasis 10**	23	chert
**Dakhla Oasis E-72-1**	23	chert
**Bir Sahara 14**	18	Nubian quarzitic sandstone

Geometrical and morphological analysis followed the method proposed by A. G. Costa [40], which consists in the 2D analysis of semi-landmarks superimposed on the outlines of artefacts, allowing comparisons between stone artefacts using comparative points and identifying differences between given populations [33, 40–43]. The method has certain limitations as it analyses only artefact’s silhouette [39, 44, 45].

The photographs and scans of the objects were aligned along the symmetry axis [46] and outlined with Inkscape (version 0.92.1). The resulting outlines were converted from JPG to TPS format using the tpsUtil64 programme (version 1.78). In the next step, 75 semi-landmarks (including one fixed landmark and 74 semi-landmarks) were superimposed, starting from one fixed landmarked located at the tip or maximal curve of the distal round extremity and also in the case of cleavers and “cleaver-like” hand-axes at the point of crossing main technological axis and distal edge (S1 Fig), with the use of the tpsDig2 automatic curve-tracing function. The landmark coordinates for all the objects were generated with the PAST 3 programme (PAleontological STatistics). All the objects were adjusted in the Procrustes analysis, yielding averaged and standardised images of all the artefacts in a given group (S2 Fig). The transformed objects were compared using principal component analysis (PCA) and the acquired image was tested using Thin-Plate Spline Deformations; this way we were able to see the tendencies in the standardisation of the acquired shape at the extreme points of both axes. Twenty-five landmarks selected proportionally to each object were examined with the use of Multivariate Analysis of Variance and Permutational Multivariate Analysis of Variance (MANOVA, PERMANOVA), to allow identification of statistical differences between the assemblages. These two tests make different assumptions about the underlying datasets, making our analysis more robust: the MANOVA tests comparing equality mean values for univariate population having Normal Distribution, the PERMANOVA tests distance of observation from centroids and is resistant to the assumptions of variance homogeneity and normality of distribution [47, 48]. For each of the tests we assumed an alpha level (0.05) which defines their statistical significance.

### Use wear analysis

In total, 15 quartzite artefacts that fulfilled the following criteria were selected for use wear analysis. Only the forms classified as tools during the technological analysis were included. The tools had to have at least one intact, functional working edge. Additionally, edge-rounding and micro-chipping was taken into consideration. Artefacts were cleaned for 2 to 5 minutes in an ultrasonic bath. All artefacts were observed under a NIKON Eclipse LV 100 with magnifications between 200× and 500×. Analysis included scanning of the entire edge of each tool, as well as the areas immediately adjacent to it. Additionally, ridges and surfaces in the middle of artefacts were evaluated for post-depositional wear. Where evidence for use wear was found, it was documented with a Hirox RH-2000 digital microscope. Images were taken with a motorised extended focus system. Enhanced digital processing including anti-halation and contrast adjustments helped to visualise the traces better and reduce the glare of the highly reflective surfaces [49].

Due to the heterogeneous nature of quartzite, surface traces develop irregularly and in patches, being more abundant on the crystals than on the matrix [50–52]. Moreover, various diagnostic alterations such as fractures, impact pits or striations, may not necessarily occur on the same edge. These properties, combined with the high reflectivity of its surface, make quartzite more challenging to analyse than homogenous materials such as chert [53]. Therefore, greater magnifications, usually between 200× and 500×, are required to observe specific surface features [54].

Although far from being the most frequently studied raw material, quartzite has gained popularity in use wear analysis in the past few years [50, 55, 56]. Numerous experiments were conducted, concerning both the formation of use wear [53, 57, 58] and post-depositional traces [59, 60]. Their results provided guidelines for observation and interpretation of quartzite use wear.

### Optically Stimulated Luminescence (OSL) dating

Samples were collected in opaque metal tubes hammered into the face of a cleaned section. The samples were then processed in the Korean Institute of Geoscience and Mineral Resources (KIGAM) and in Gliwice Absolute Dating Method Centre (GADAM) laboratories under subdued red light. Sunlight exposed material was removed and retained for dose rate measurements.

Carbonates and organic matter were removed from the unexposed sample using 1M HCl and 20 volumes H_2_O_2_ respectively. Pure quartz was extracted from the 90–212 or the 90–125 μm fractions using density separations at 2.62 and 2.70 g/cm^3^ and a subsequent HF acid etch (23M HF for 40 min followed by an 10M HCl rinse). Refined quartz was deposited as a monolayer on aluminium discs using Silkospray silicone oil.

Single-aliquot regenerative-dose procedures [61] were applied to all samples using preheats of 260, or 220°C for 10 s prior to measurement of the natural/regenerated luminescence intensity (PH1), and 160, or 220°C for 10 s prior to measurement of the test dose luminescence intensity (PH2; S1 Table). These temperatures were deemed the most appropriate to preheat the samples following dose recovery preheat tests (S3 Fig).

At KIGAM measurements were carried out using a Freiberg Instruments Lexsyg Smart system TL/OSL reader equipped with a Hamamatsu bi-alkaline photomultiplier tube (H7360-02), while at GADAM measurements were performed on a Daybreak Model 2200 reader. Irradiations were carried out using ^90^Sr/^90^Y beta sources. Stimulations were carried out at 125°C for 60 s using blue light emitting diodes. Aliquots were heated at 5°C/s during all heating steps and a 10 second pause at 125°C prior to optical stimulation was used, to allow for the lag in temperature between the thermocouple and sample. The OSL intensity is that recorded during the first 1.5 seconds of stimulation with a background signal subtracted. All growth curves were fitted using a saturating exponential plus linear function, a typical growth response curve is displayed in S4 Fig. The OSL characteristics of the quartz from Sudan show a rapidly decaying signal and continuously growing dose response curve, which makes it well-suited for application of the SAR protocol used in this study.

Recycling ratios and recuperation were calculated to monitor the performance of the SAR procedure [61], while sample purity was assessed by measuring an IR depletion ratio [62]. Aliquots not yielding recycling or IR depletion ratios consistent with unity or displaying recuperation greater than 5% of the natural signal were rejected. The sample equivalent dose was calculated using the Central Age Model (CAM) [63] on the D_e_ values obtained from accepted aliquots. Radial plots showing the spread of the D_e_s and the calculated CAM for each sample are presented in S5 Fig and Table 2.

**Table 2:** OSL data summary and ages.

Sample^1^	Radionuclide concentrations^2^			Sample depth	Cosmic dose rate^3^	Total dose rate^4^	Equivalent dose^5^	Age^6^
	K (%)	U (ppm)	Th (ppm)	(m)	(Gy/ka)	(Gy/ka)	(Gy)	(ka)
**EDAR7-1 (K)**	0.28 ± 0.01	0.39 ± 0.09	0.75 ± 0.04	2.7 ± 0.05	0.15 ± 0.02	0.54 ± 0.03	152 ± 12	280 ± 27
**EDAR7-2 (K)**	0.34 ± 0.02	0.64 ± 0.12	1.14 ± 0.05	2.2 ± 0.05	0.16 ± 0.02	0.69 ± 0.03	138 ± 5	199 ± 12
**EDAR7-3 (K)**	0.31 ± 0.02	0.45 ± 0.10	0.87 ± 0.04	1.0 ± 0.05	0.19 ± 0.02	0.63 ± 0.03	100 ± 8	158 ± 15
**EDAR7-4 (K)**	0.54 ± 0.02	0.45 ± 0.02	1.56 ± 0.06	0.8 ± 0.05	0.19 ± 0.02	0.90 ± 0.03	19.8 ± 2.9	22 ± 3.4
**EDAR7-5 (K)**	0.60 ± 0.02	1.0 ± 0.10	1.50 ± 0.06	0.6 ± 0.05	0.20 ± 0.02	1.08 ± 0.04	10.9 ± 2.2	10 ± 2.1
**EDAR-135-S6 (G)**	0.12 ± 0.02	0.24 ± 0.02	0.79 ± 0.12	3.6 ± 0.05	0.14 ± 0.01	0.43 ± 0.02	168 ± 6	391 ± 30

^1^ Samples measured at KIGAM are followed by (K), while those measured at GADAM are followed by (G).

^2^ Radioisotope concentrations were measured using high resolution gamma spectrometry and converted to dose rates following Guérin et al. (2011) [64].

^3^ Cosmic dose rates were calculated following Prescott and Hutton (1988) [68] and using overburden densities of 1.8 g/cm^3.^

^4^ The total dose rates were corrected for grain sizes of 90–212 μm and 8 ± 3% moisture content.

^5^ Equivalent dose rates were calculated using the Central Age Model (CAM) [63].

^6^ The datum of the age calculation is 2019.

Radioisotope concentrations were measured with Canberra gamma spectrometers with HPGe detectors–the samples did not display any sign of radioactive desequilibria. Since the moisture content of the samples is expected to have changed during burial, a value of 8 ± 3% was assumed for all samples, to account for the plausible range of past conditions and humidity changes.

Beta and gamma dose rates were calculated from radioisotope concentrations and moisture contents using the conversion factors of Guérin et al. [64]. The alpha dosed rind of the quartz grains was assumed to have been removed by HF etching [65] and hence the alpha dose rate was taken to be zero. Beta dose rates were corrected for grain size using the attenuation factors of Guérin et al. [66] and an etch attenuation factor after Bell [67]. Cosmic ray dose rates were calculated based on the altitude, latitude and longitude, present-day burial depth and overburden density of the sample [68]. Overburden densities of 1.8 g/cm^3^ were assumed. The CAM D_e_, the dose rates and ages for each sample were calculated using DRAC [69] and are presented in Table 2.

## Results

### Composite sedimentary stratigraphy of the EDAR area

**Fig 4:**
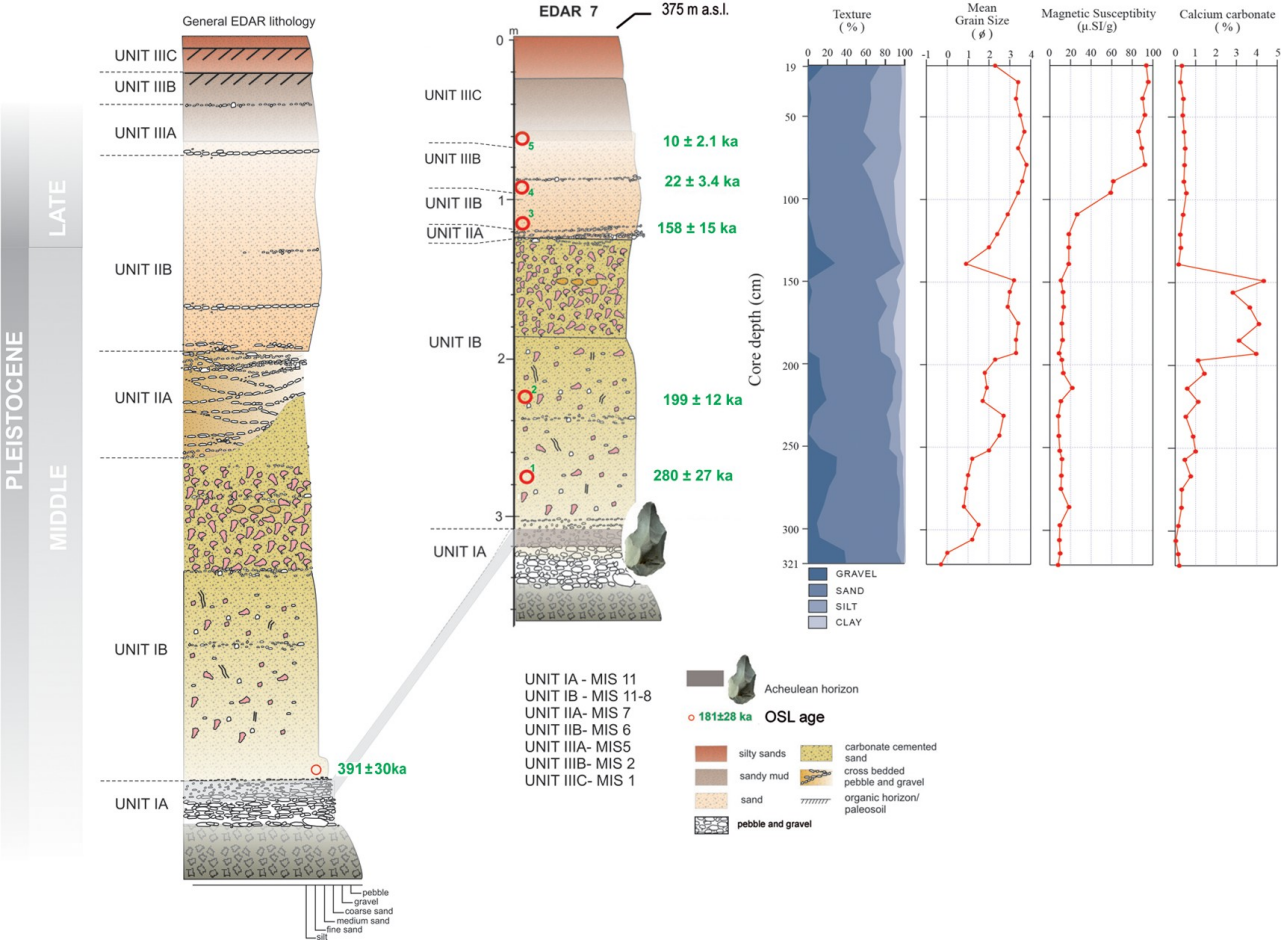
Sediment lithology and chronology of EDAR site 7. Sequence of OSL dates (in green) above the Acheulean horizon. Lithology of the site: 0-30cm—fine-grained, dark brown sand; 30-70cm—fine-grained sand with organic matter, grey-brown, buried soil; 70–105 cm—medium-grained sand with individual pebbles, yellow-brown; 105–120 cm—gravel sand with gravel laminates; 120–250 cm—coarse-grained, light brown sand enriched with carbonate concretions, single layers of gravel; 250–300 cm—sands with gravels, fining upwards; 300–350 cm—gravel with carbonate binder, gravel with the long axis aligned along the N-S course; from 350—weathered rocks of the bedrock, rhyolite.

A thick sedimentary sequence containing a Pleistocene to Holocene succession was excavated in the EDAR area. Detailed analysis of the Pleistocene fluvial sediments has been carried out through five correlated profiles, each subdivided into units (Fig 4). The simplified cross section of EDAR sedimentary deposits shows a ~5 m thick stratigraphy divided into three units (Units I-III) bounded by erosional surfaces [32]. Unit I consists of a stratified pebble gravel unit (Unit IA) and massive sand with abundant calcium carbonate nodules (Unit IB), suggesting that the sediment deposition occurred in braided streams followed by calcium carbonate precipitation under arid climatic conditions. Unit II comprises planar-to cross-stratified pebble gravel (Unit IIA) overlain by a metre-thick, massive and carbonate-cemented sand (Unit IIB), indicating another episode of fluvial incision and sediment deposition followed by carbonate precipitation under more arid climatic conditions. Unit II is overlain by a thin and continuous gravel layer, probably representing a desert pavement or relict paleo-topographic surface which may be produced by the removal of sand and dust by wind and intermittent rain. The overlying Unit III comprises yellow to dark brown silt sands, which can be divided into three sub-units based on subtle changes in grain size and sediment colour. The unit is structureless but contains rare pebbles and tubular voids interpreted as rhizoliths.

### Sediment lithology of EDAR 7

**Fig 5:**
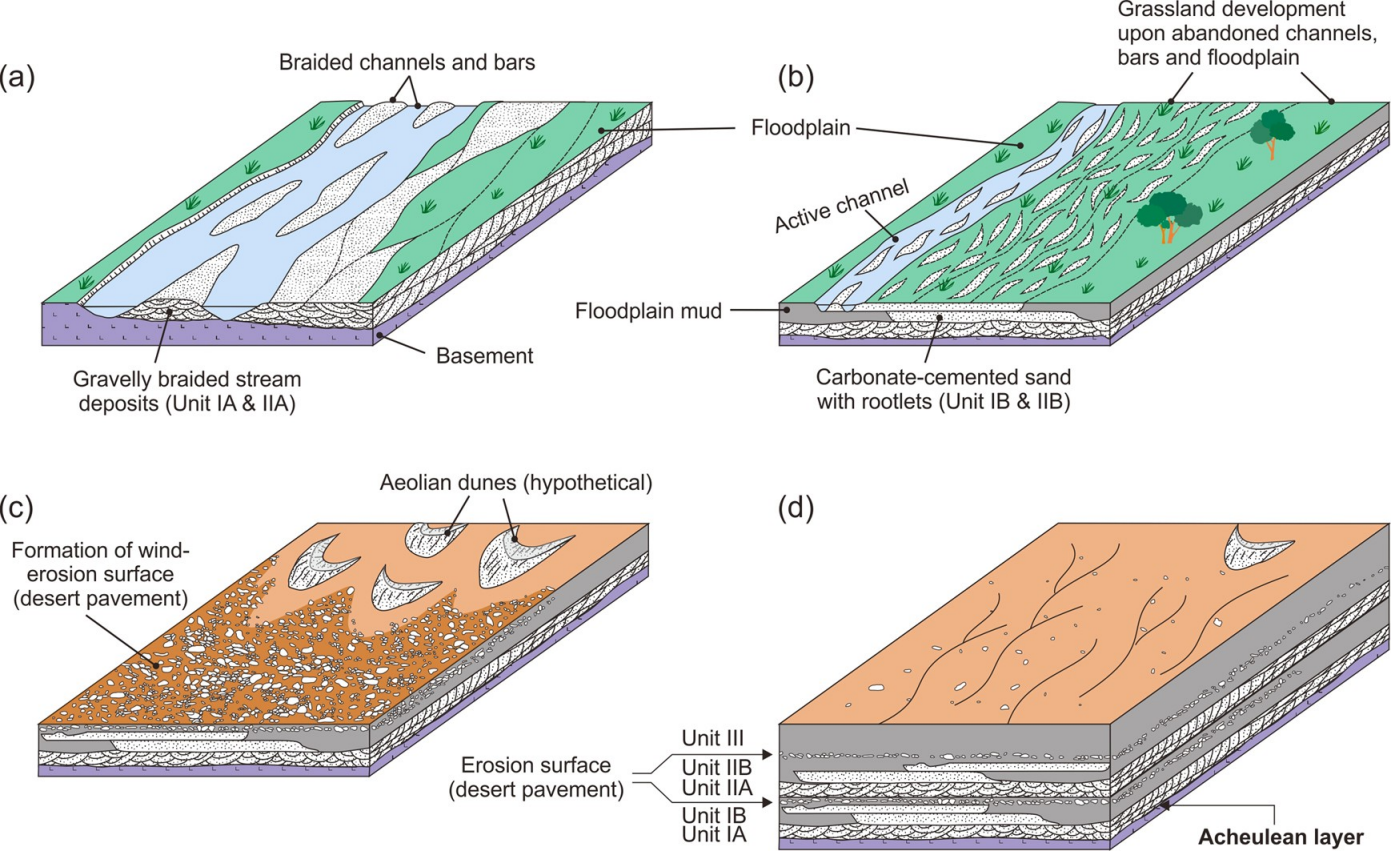
A schematic illustration of the changing depositional environments in the EDAR area. (a) The area was initially characterised by gravelly braided streams because of wet climatic conditions, resulting in deposition of Unit IA, which contains an Acheulean layer, upon the basement rocks. (b) During a transition to arid conditions, the majority of the area changed into a grassland with occasional floods, resulting in precipitation of calcium carbonate in sandy fluvial deposits of Unit IB. (c) During a period of extreme aridity, the area was subject to wind erosion and resulted in the formation of a desert pavement, which is recognised by a thin and continuous gravel layer in section. (d) The wet-to-dry cycle of climate change, depicted in (a), (b) and (c), was repeated, resulting in superposition of similar fluvial deposits (Unit II) upon Unit I. Afterwards, the area was subject to more intense Aeolian processes together with rare overland flows resulting from rare rainfall events.

The 3.5 metre-thick sedimentary sequence at EDAR 7 sits upon rhyolite bedrock (Fig 4). The lowermost layer (UNIT IA) contains an Acheulean horizon. This unit is composed of a cross-stratified thin alluvial pebble bed containing rare cobbles with a diameter of up to 30 cm, topped by well-rounded gravels supported in a well-sorted sand matrix. The orientation of elongated pebbles indicates north to south paleo-flow direction. Overlying Unit IB is 1.5 m thick and composed of massive medium- and coarse-grained sands cemented with secondary calcium carbonate, also containing a band of carbonate concretions. The overall features of this unit suggest rapid deposition of sand by floods, followed by precipitation of calcium carbonate under dry climatic conditions. Unit IIA starts at a depth of about 120 cm, and is characterised by a cross-stratified gravel layer indicating the fluvial origin of the sediment. The characteristics of this deposit suggest that the sediments were deposited from braided streams, which were probably shallow and ephemeral and had variable paleo-current directions and discharge (Fig 5). The variations in fluvial style and discharge between UNIT IA and IIA are connected with climatic changes and resultant river network modifications. Above this unit carbonate-cemented sands with concretions (UNIT IIB) appear again. The upper layers of the site comprise massive structureless fine-grained sands, indicating periods of slow but continuous sedimentation and slow denudation. An intermediate silty sand layer marks a period of reduced clastic input and morphological stability. Sediment facies starting with gravel bed stream deposit and terminating with structureless silty sands indicate environmental changes from stream channel to arid savannah and grassland with the development of a soil profile. The unconformity surfaces between Units II and III are erosive surfaces occurring locally. These are desert pavements which have formed through winnowing sand and dust by the wind and intermittent rain, leaving the larger clasts behind. The desert pavements indicate the onset of intense aeolian processes.

### Multi-proxy records of EDAR 7

The mean grain size is highly variable through the EDAR 7 sequence. Grain size analysis and magnetic susceptibility therefore provides important clues to the sediment provenance, transport and depositional condition. It generally decreases with depth and the primary trends agree well with granulometric texture (Fig 4). Relative mean grain size decreases at two intervals, from 157 to 201 cm (Unit IB) and from 254 to 331cm (Unit IA), respectively recognised as fluvial levels. Magnetic susceptibility is the degree to which a material can be magnetised by an external magnetic field. Therefore, magnetic susceptibility provides information about sediment composition. Magnetic susceptibility (MS) at EDAR 7 generally increases with depth. While the mean values of MS range between 10 and 20 × 10^−5^ SI in general, the highest MS occurs below 254 cm (average 84.4× 10^−5^ SI). MS maximum occurs between 271 and 331 cm (Unit IA), where values up to 95.4 × 10^−5^ SI were measured. In EDAR site 7, we may interpret that high MS in the lowermost part, mostly fluvial in origin with secondary calcium carbonate concentrations, is derived from high concentration of detrital magnetic minerals during fluvial sedimentation. Secondary calcium carbonate content shows an abrupt increase from 200 cm in depth with a strong cyclicity, conspicuously increasing up to 4.0% between 153 and 201cm, where mean grain size decreases.

### Chronology

The Acheulean layer (unit IA) was too cemented to sample, but a sample in the lowermost part of unit IB in EDAR 7 was taken, providing a minimum age for the Acheulean horizon. A similarly positioned sample was also taken at the nearby site EDAR 135, to refine the dating of the Acheulean layer. At EDAR 7, samples were also taken in the middle of unit IB, in unit IIA, and in the overlying sand layers units IIB, and III.

The equivalent dose distributions from these samples have a range of overdispersion values (OD; the relative spread of equivalent doses after measurement uncertainties are excluded). The samples EDAR7-4 and EDAR7-5, which were taken from aeolian sand devoid of archaeological material overlying the entire studied sections, have OD values of ~50 and 70%, which is far higher than what would be expected if they were well-bleached and unbioturbated. A geological analysis of units IIB and III shows that these layers are affected by soil formation processes which may have led to the vertical movements of sand grains. The use of the minimum age model was therefore not considered for these samples despite their large OD, since it is highly sensitive to the inclusion of younger grains into an older sediment body [70]. The CAM was used for the age calculations for these two samples, though if younger grains are intruded into these levels by pedogenic processes, this approach will yield age underestimates. Unit IIB and III were dated to 22 ± 3.4 (EDAR7-4) and 10 ± 2.1 ka (EDAR7-5) respectively, and most likely correspond to MIS2 arid episodes favouring the deposition of aeolian sands which were fixated and bioturbated at the beginning of the Holocene.

Samples EDAR7-1, EDAR7-2, EDAR7-3 and EDAR135-S6 have relatively low OD, ranging from ~10 to ~30%. These values suggest that the sediments were well-bleached prior to deposition, and not subsequently subject to bioturbation. Consequently, the Central Age Model (CAM) was used to calculate ages for these samples [63]. Despite the measurements of De’s above 100 Gy the samples did not show any sign of saturation in EDAR7. The samples taken at the contact between units IA and IB were dated in EDAR 7 to 280 ± 27 ka (EDAR7-1) and to 391 ± 30 ka in EDAR 135 (EDAR135-S6), suggesting that the Acheulean level in EDAR 7 is MIS9, but possibly MIS11-13 or older. The morphology and wear-conditions of artefacts in UNIT IA indicate that they may have been reworked by overland flows and re-deposited into younger sediment (280 ka) reinforcing the hypothesis that these artefacts are at least MIS11-13.

The other sample taken in the middle of unit IB at EDAR 7 was dated to 199 ± 12 ka (EDAR7-2). EDAR7-3 was sampled directly at the interface between unit IIA and IB, and yields an age of 158 ±15 ka. This dates the deposition of unit IIA to between 199 ± 12 ka and 158 ±15, suggesting an MIS7/6 age. The ~100 ka hiatus between the units IIA and IIB probably relates to erosion during intense arid episodes of the uppermost unit IIIA (visible in other sites in EDAR). Moreover, erosion of sediments laid down during periods of low sedimentation rates could have been enhanced by tectonic activity, i.e. during the uplift of the Nubian massif [71].

### EDAR 7 lithic inventory

#### Artefacts taphonomy

Preservation state (S2 Table), degree of erosion and breakage patterns can be used to determine the taphonomic history of the lithic inventory. Less than 0.5% of the assemblage is fresh. The remaining 99.5% display only slight traces of abrasion or is abraded. The degree of abrasion in all raw material groups is approximately the same. Over 99% of both quartzites and rhyolites are abraded, which reflects the fluvial environment within which the assemblage was found. Such deposition, especially within a high-energy fluvial system, coupled with the brittleness of the raw material and different aspects of knapping and usage processes explain the breakage patterns:13.5% of quartzite and 65.9% of rhyolite artefacts are incomplete. No evidence of contact with fire was observed on the artefacts.

### General structure

The EDAR 7 assemblage includes 918 artefacts weighing 115.4 kg in total (Table 3). Raw materials used are dominated by quartzite (90.5%) and fine-grained greenish rhyolite (8.7%), both available in the immediate surroundings. The Hudi chert, identified from outcrops in the vicinity of EDAR 7 and archival sites [27] was used only occasionally (only one artefact in EDAR 7, but this raw material was also present at other EDAR sites). The assemblage consists of products of all stages of core use and tool production. Among 138 tools (15% of the assemblage), 44 large cutting tools (LCT) were identified, which is 32% of the tool assemblage. Apart from one hammer stone, the remaining tools are retouched flake forms, hardly ever exceeding 3 cm in length (67% in the category of tools). Over 7% of the assemblage are cores, almost without exception made from quartz. These cores are very variable in size, ranging from giant cores to small specimens. Blanks with waste (debris) constitute over 77% of the assemblage. The most numerous in this group are chips, making up over 40% of the assemblage.

**Table 3:** Lithic inventory from EDAR 7.

Basic Inventory Categories	Metamorphic				Sedimentary				Igneous		Total			
	Quartzite		Quartzitic sandstone		Chert		Hudi Chert		Rhyolite					
	n	%	n	%	n	%	n	%	n	%	n	%	weight (g)	%
**LCT**	27	3,2	1	100	1	100	-	-	15	18,75	44	4,79	26684	23,11
**Ret. tool**	77	9,2	-	-	-	-	-	-	16	20	93	10,13	10134	8,78
**Core**	67	8	-	-	-	-	-	-	2	2,5	69	7,52	49225	42,64
**Flake**	226	27	-	-	-	-	1	1	29	36,25	256	27,89	22798	19,75
**Debris**	76	9,1	-	-	-	-	-	-	7	8,75	83	9,04	4304,5	3,73
**Chip**	360	43,1	-	-	-	-	-	-	11	13,75	371	40,41	523	0,45
**Hammerstone**	1	0,2	-	-	-	-	-	-	-	-	1	0,11	196,5	0,17
**Worked slab**	1	0,2	-	-	-	-	-	-	-	-	1	0,11	1578	1,37
**Total**	835	100	1	100	1	100	1	100	80	100	918	100	115443	100

General categories and raw materials.

### Cores

**Fig 6:**
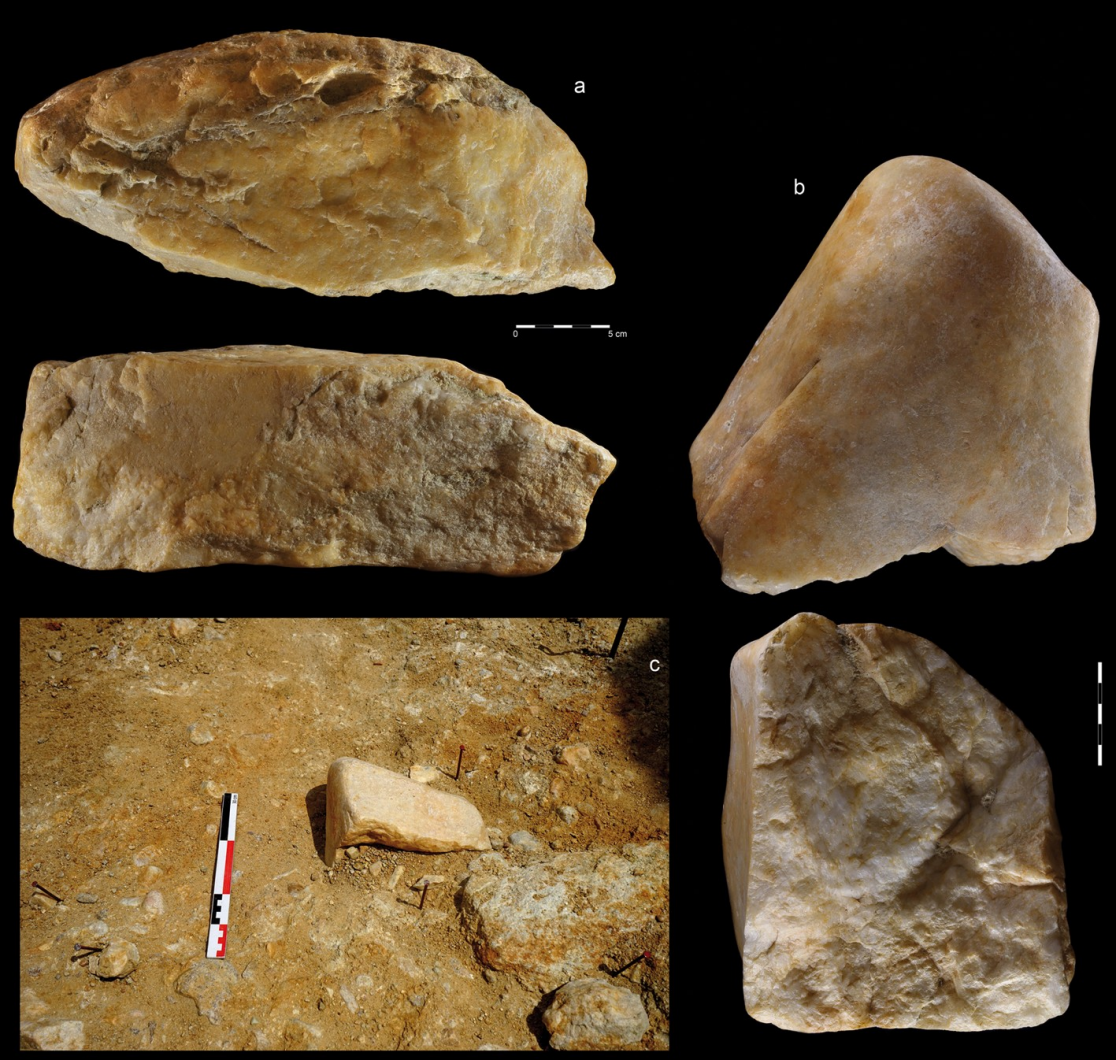
Giant cores from EDAR 7. (a) quartzite amorphic giant core, size: L-342 mm, W-115 mm, Th-140 mm, weight-9,2 kg (art. no. 555); (b, c) quartzite amorphic giant core, size: L-200,9 mm, W-160 mm, Th-190 mm, weight-6,9 kg (art. no. 83).

**Fig 7:**
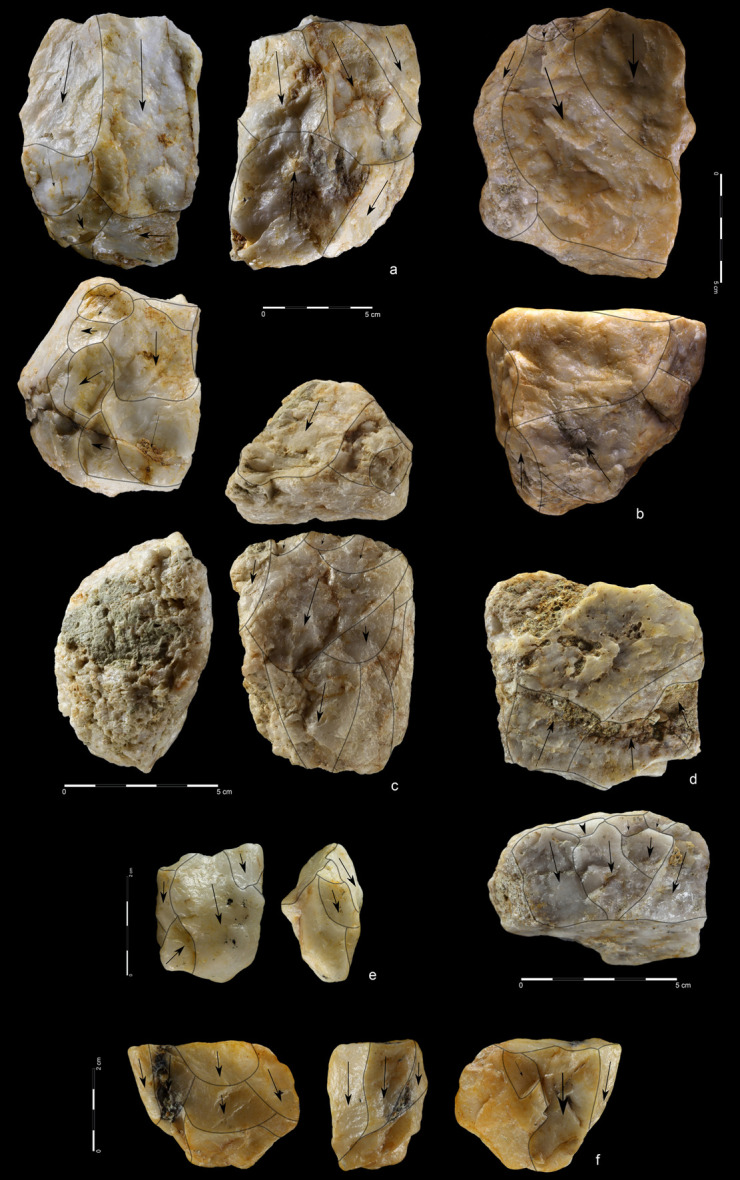
Quartzite unidirectional cores from EDAR 7. (a) art. no. 96, (b) art. no. 480, (c) art. no. 132, (d) art. no. 419, (e) art. no. 268, (f) art. no. 155. Note the difference in size between cores (i.e. max length of core no. 96–108 mm, core no. 268–27 mm).

Cores are abundant at EDAR 7, constituting 7.5% of the inventory (see S3 Table for the frequency of individual categories of cores by raw material group). With the exception of two rhyolite examples, all cores are made of quartzite. In terms of degree of exploitation, over 70% of cores are considerably exploited blocks (S4 Table). Even though the cores belong to ten separate categories, closer examination reveals that three categories dominate, quite well reflecting the implemented technological measures. Initial cores and amorphic forms, which usually display negatives of removal situated chaotically on the perimeter of the blocks, jointly constitute over 40% of all the cores. This group also includes two giant cores, which do not display evidence of advanced reduction (Fig 6). The second group in terms of its size is single-platform, unidirectional cores with crude striking platforms– 30% (Fig 7). The third characteristic group is the cores with negatives of flakes knapped on more than two platforms, where evidence of exploitation is seen on a greater part of core’s surface. These three groups constitute over 85% of all the cores. Cores from the remaining categories occur only individually, even though discoidal cores constitute a substantial category (S6 Fig).

In terms of size and weight of cores the assemblage is diversified (S5 Table). Giant cores are present; the largest is longer than 34.0 cm and its weight exceeds 9 kg (Fig 5). These blocks were used in biface manufacture and have many analogies at Acheulean sites [72–74]. On the other hand, the assemblage also includes quartzite cores of microlithic proportions, whose length and width hardly ever exceeds 3–4 cm (S7 and S8 Figs). These blanks were used to produce tools, such as small endscrapers and perforators.

### Debitage and waste

**Fig 8:**
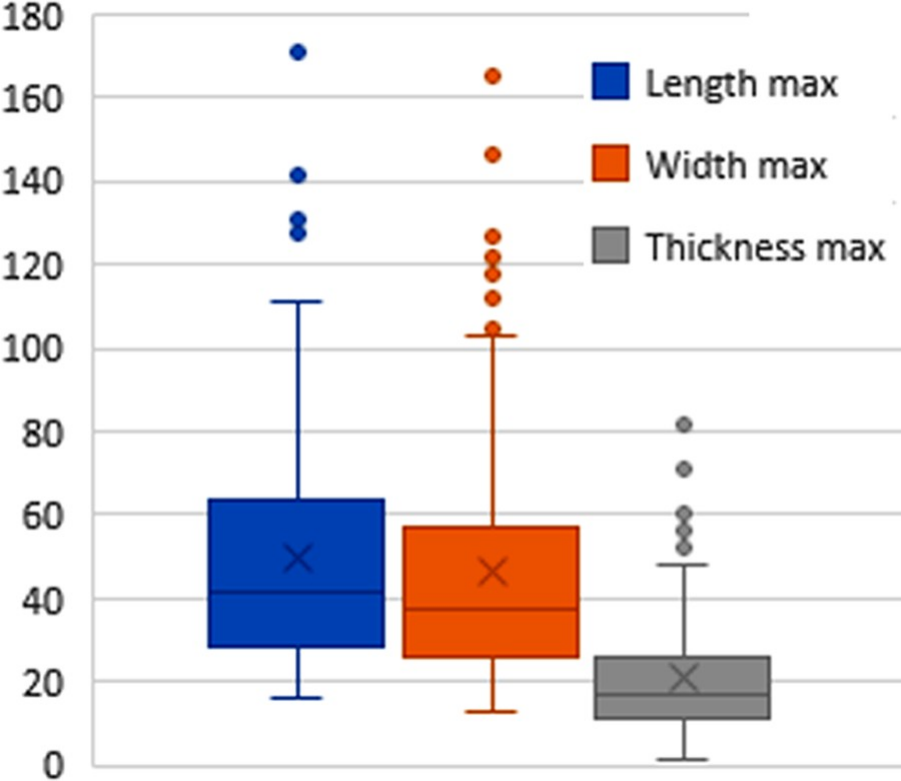
Dimensions (mm) of complete flakes (n = 197).

Debitage and waste (debris and chips, i.e. flakes with length ≤ 15 mm), numbering 710 artefacts, constitute over 77% of the EDAR 7 assemblage (Table 3). Blanks and waste weighed over 27 kg, which is ~24% of the weight of the EDAR 7 assemblage. No products which could be interpreted as blades were observed among the blanks, so this category is made up by unretouched flakes, including core management pieces and technical forms, such as hand-axe shaping flakes. Fig 8 and S6 Table present diversified lengths and weights of complete flakes (67% of blanks). Mean length and width of complete flakes is near 50 mm, with a mean thickness of ~20 mm.

**Fig 9:**
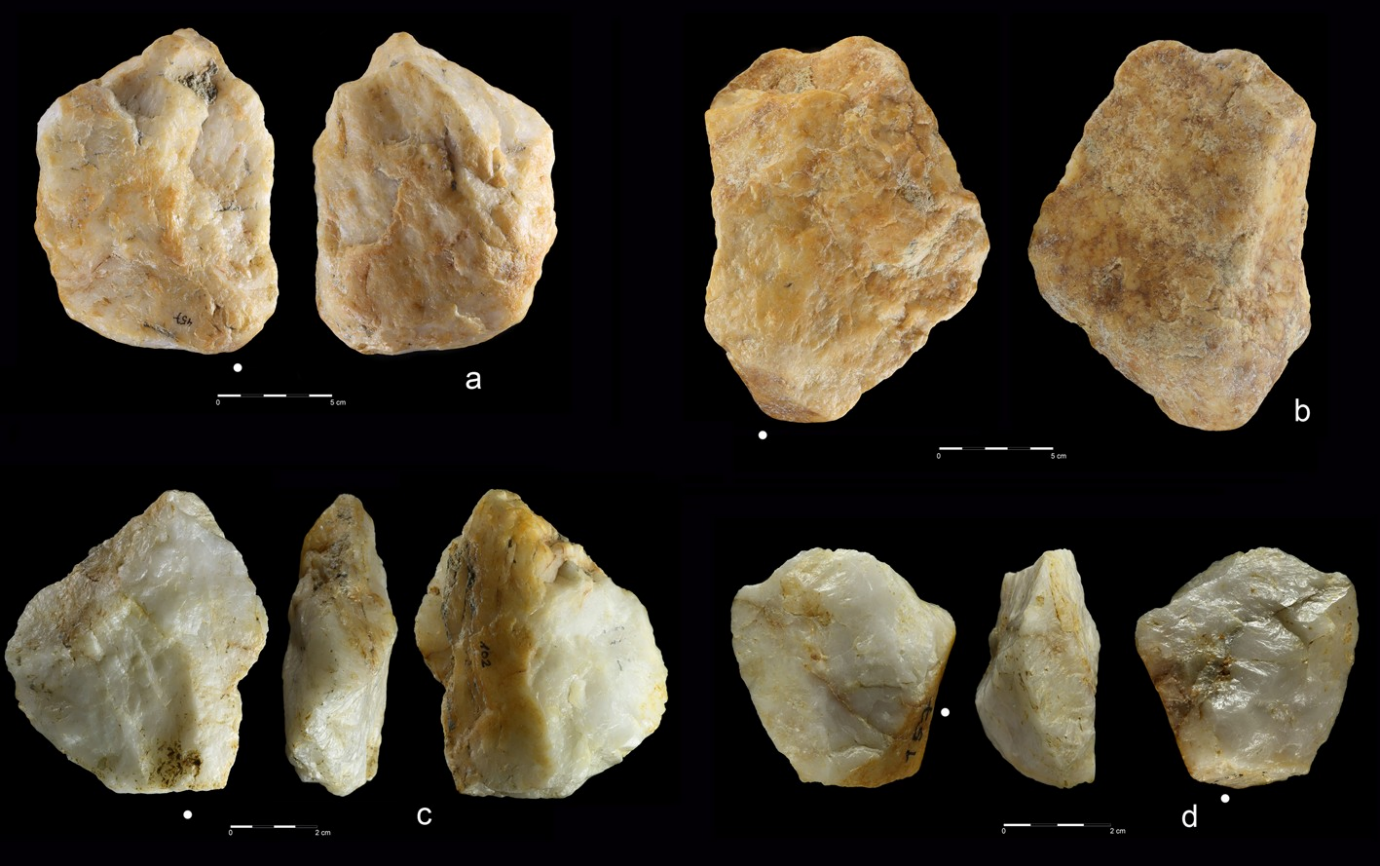
Selection of flakes from EDAR 7. Cortex flakes: (a) art. no. S7, (b) art. no. 517, (c) art. no. 102. Dorsally plain flake: (d) art. no. 157. White dot marks the impact point and butt.

The analysis of natural surfaces on the dorsal sides of flakes shows that over 20% are non-cortical. The remaining products, over 75%, are partially or completely cortical (Fig 9). If the two categories with the greatest contribution of cortex (51%-100% of cortex) are treated jointly, they constitute ~40% of flakes. This suggests that preliminary flaking took place at the site. Common presence of natural surfaces is also clearly seen in the case of the direction of blow on the dorsal side (S7 Table) and the types of flake butts (S8 Table). In the case of the direction of blow on the flakes dorsal side, in as many as 30% of products they are invisible (dorsal side covered by cortex). Presence of unidirectional negatives is in principle the same as multidirectional ones (more than two directions) and each constitutes ~30% of the assemblage of flakes. This reflects preliminary unidirectional exploitation of frequently big cores on the one hand, and on the other it is the evidence of advanced multidirectional exploitation of small quartzite blocks.

Although flakes with plain butts were dominant (>40%), forms with cortical butts were present and constituted a significant fraction (>20%). Overall, butt attributes prove that limited preparation measures were used in core reduction (fewer than 3% are prepared). Hard percussors were used in stone working, which is substantiated by, e.g. thickness of the flakes and absence of faceted butts and lip [75, 76]. One hand-axe edge modification flake was identified in the assemblage, but it is possible that some multidirectional quartzite blanks are also connected with this activity.

Two products with two ventral faces were identified among the flakes (Fig 9D). Small forms of this kind are known as ‘dorsally plain flakes’ as defined by Dag and Goren-Inbar [77], while larger ones are known as Kombewa flakes [78]. While the former are not intentional products, the latter were predetermined with the intention of biface production.

The blanks group includes 3 quartzite flakes, larger than 10 cm and of substantial weight (the heaviest specimen exceeds 1.5 kg–see S9 Table). Such forms can be defined as giant flakes *sensu* M. Kleindienst [79] or Clark [80]. Potentially, they could have been used in the production of bifacial tools [81].

### Tools

#### Metrical and morphological features of bifacial tools

**Fig 10:**
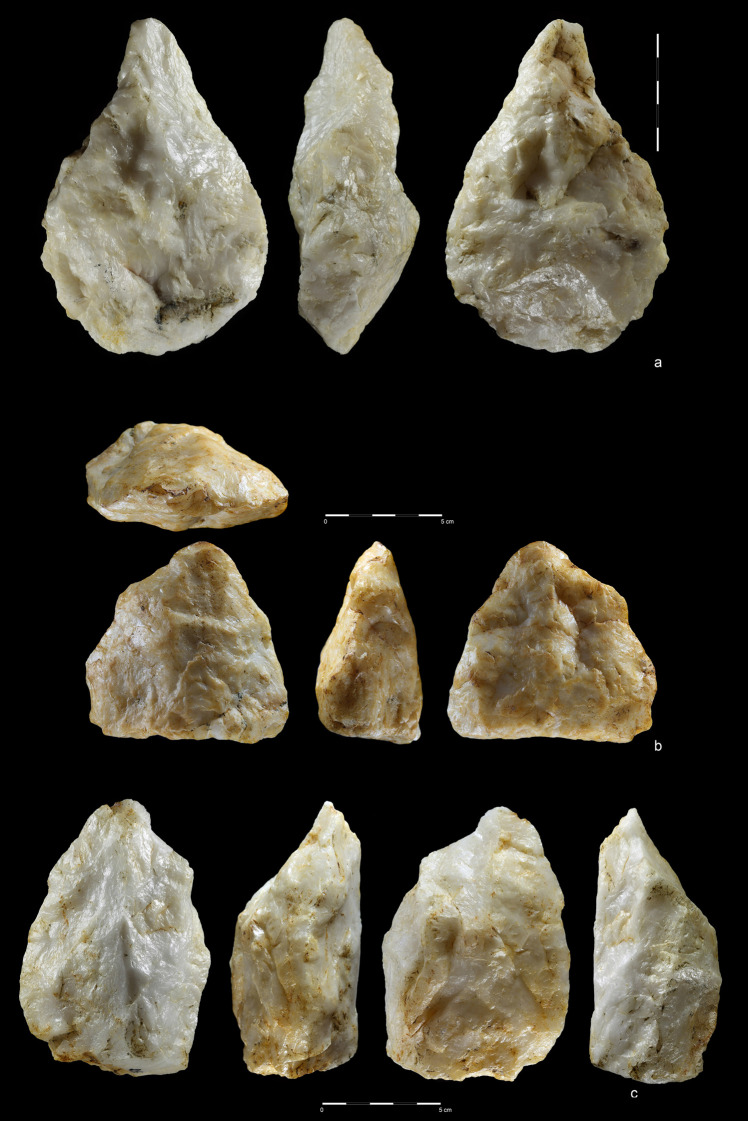
Hand-axes. Quartzite. (a) art. no. 97, (b) art. no. 156, (c) art. no. 370.

**Fig 11:**
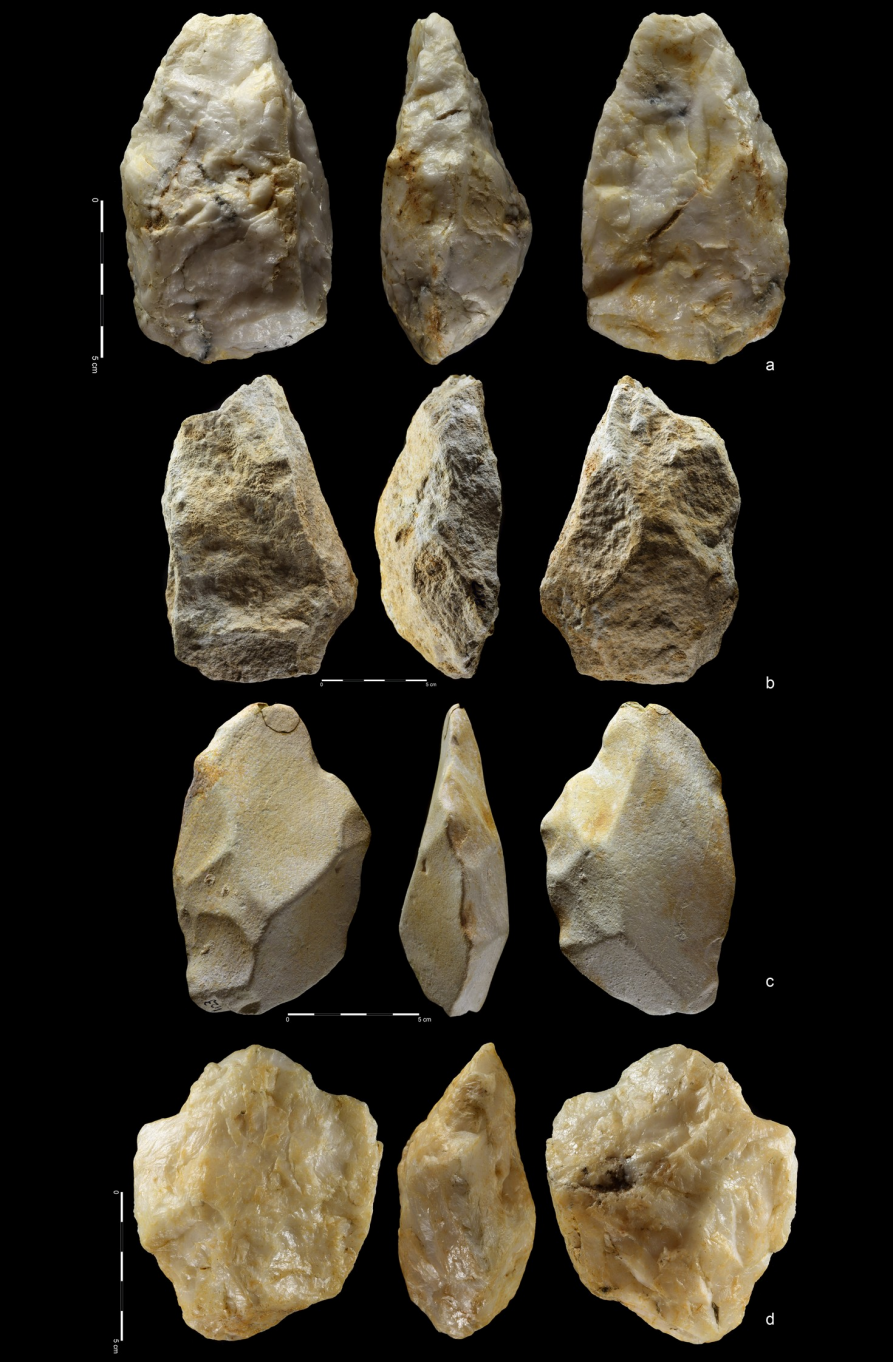
Hand-axes cleaver-like. Quartzite (a,d), rhyolite (b,c). (a) art. no. 234, (b) art. no. 208, (c) art. no. 423, (d) art. no. 465.

**Fig 12:**
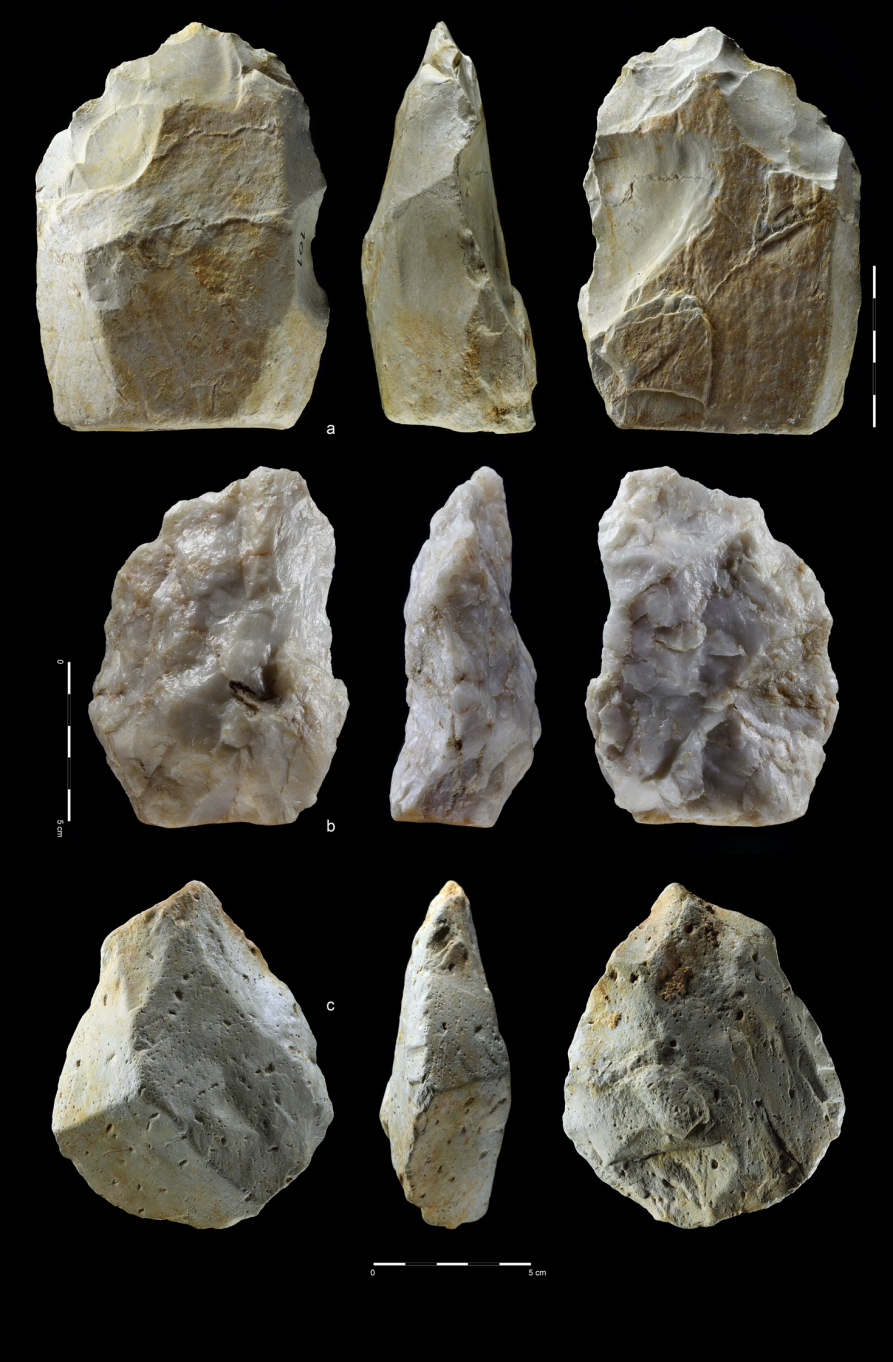
Hand-axes. Rhyolite (a,c), quartzite (b). (a) art. no. 101, (b) art. no. 178a, (c) art. no. 45.

**Fig 13:**
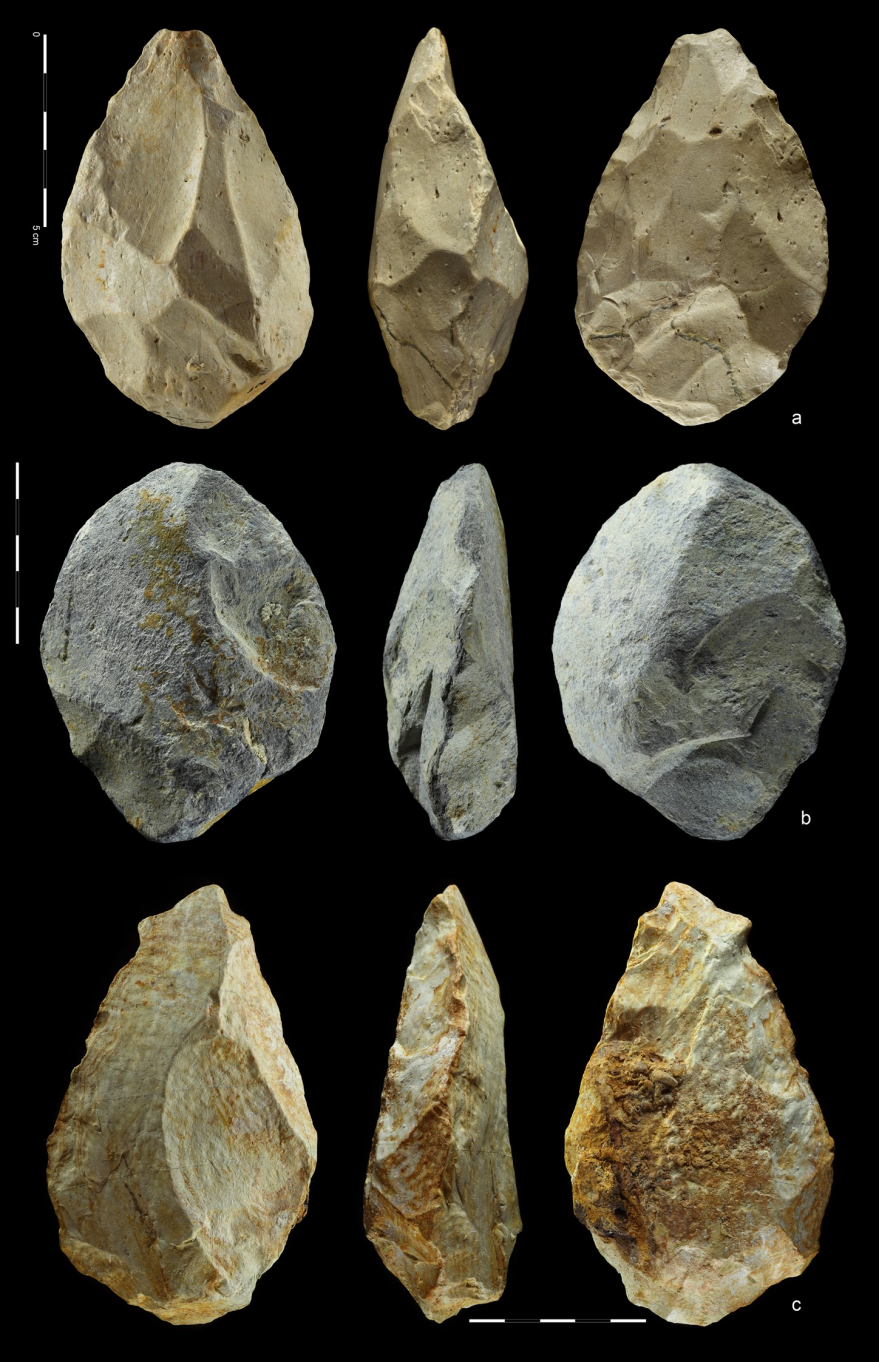
Hand-axes. Rhyolite. (a) art. no. 306, (b) Hand-axe on Kombewa flake (art. no. 323), (c) Hand-axe on flake (art. no. 326).

**Fig 14:**
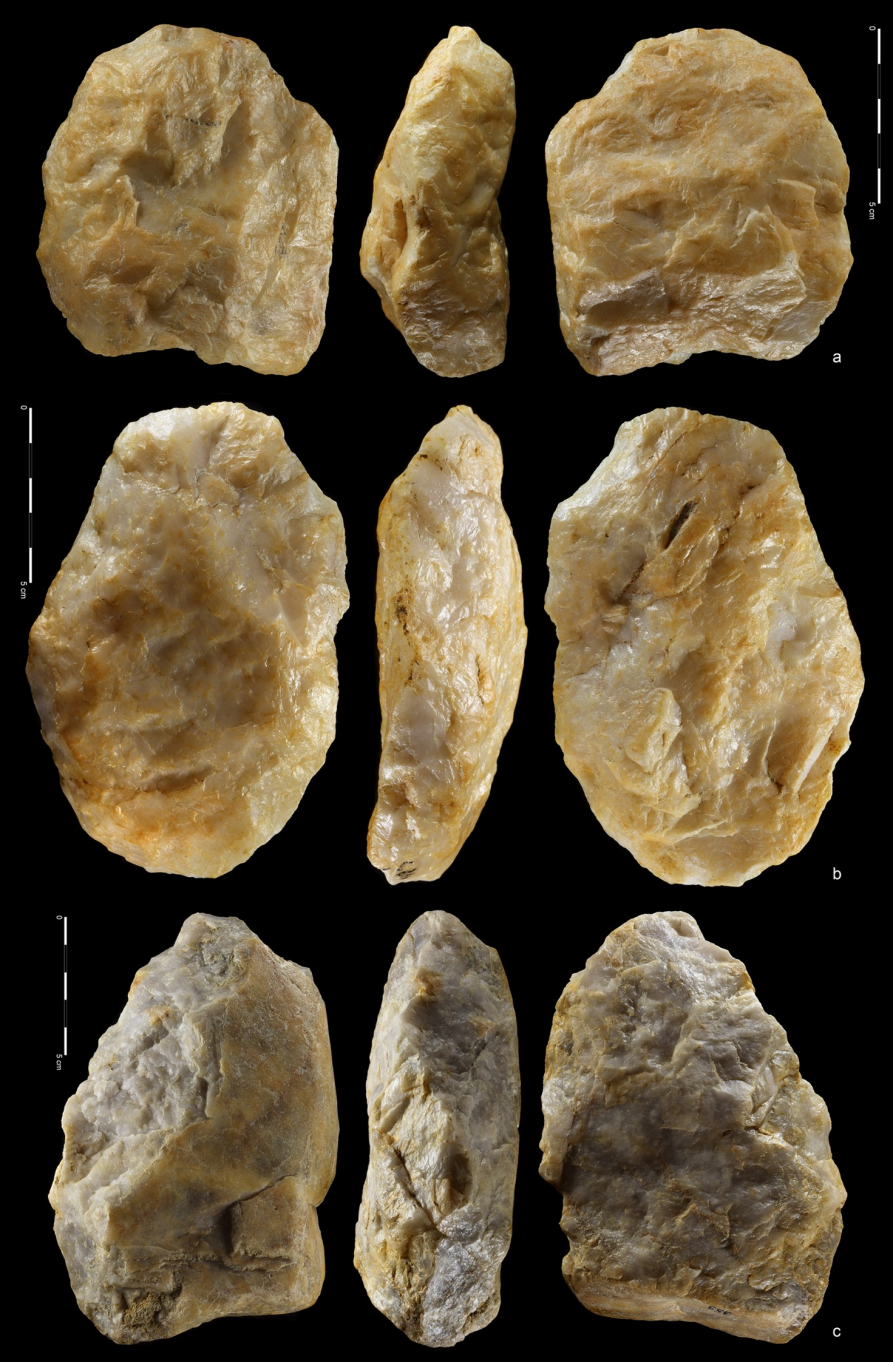
Hand-axes. Quartzite. (a) art. no. 24, (b) art. no. 163, (c) art. no. 353.

**Fig 15:**
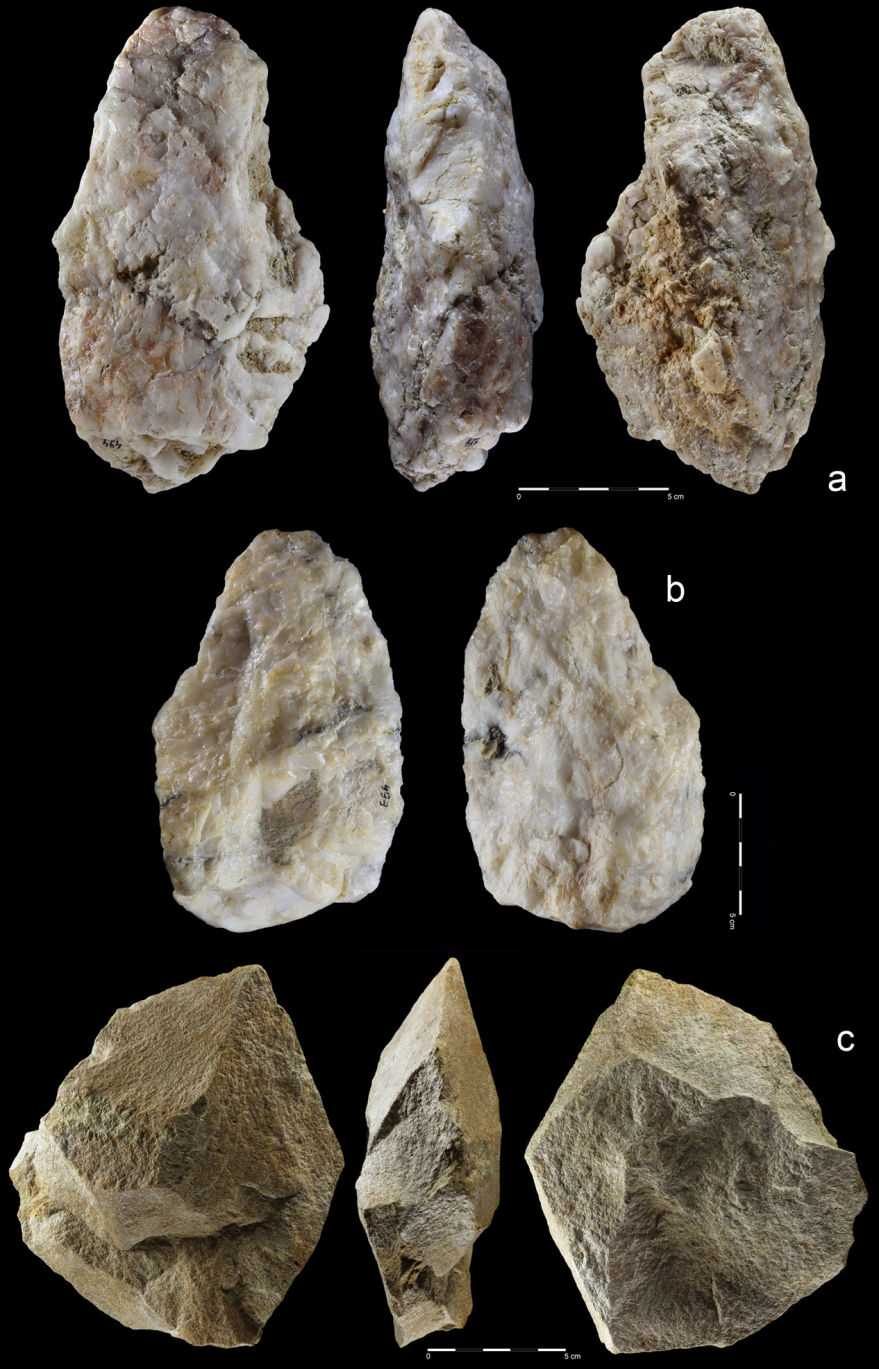
Hand-axes (a, b) and cleaver (c). Quartzite. (a) art. no. 494, (b) art. no. 393. Rhyolite. (c) art. no. 416.

**Fig 16:**
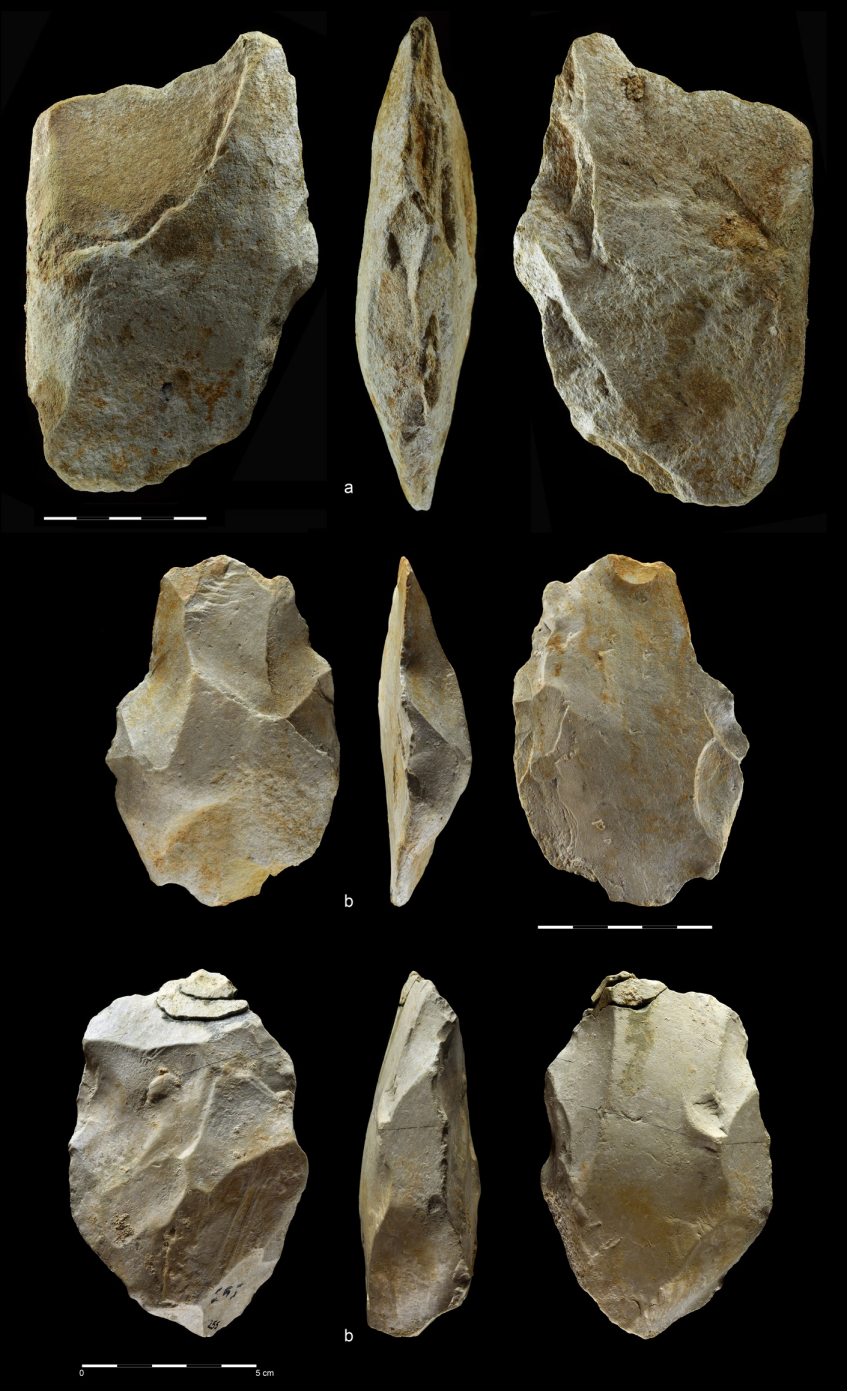
Cleavers on flakes. Rhyolite. (a) art. no. 18, (b) art. no. 324, (c) art. no. 255.

**Fig 17:**
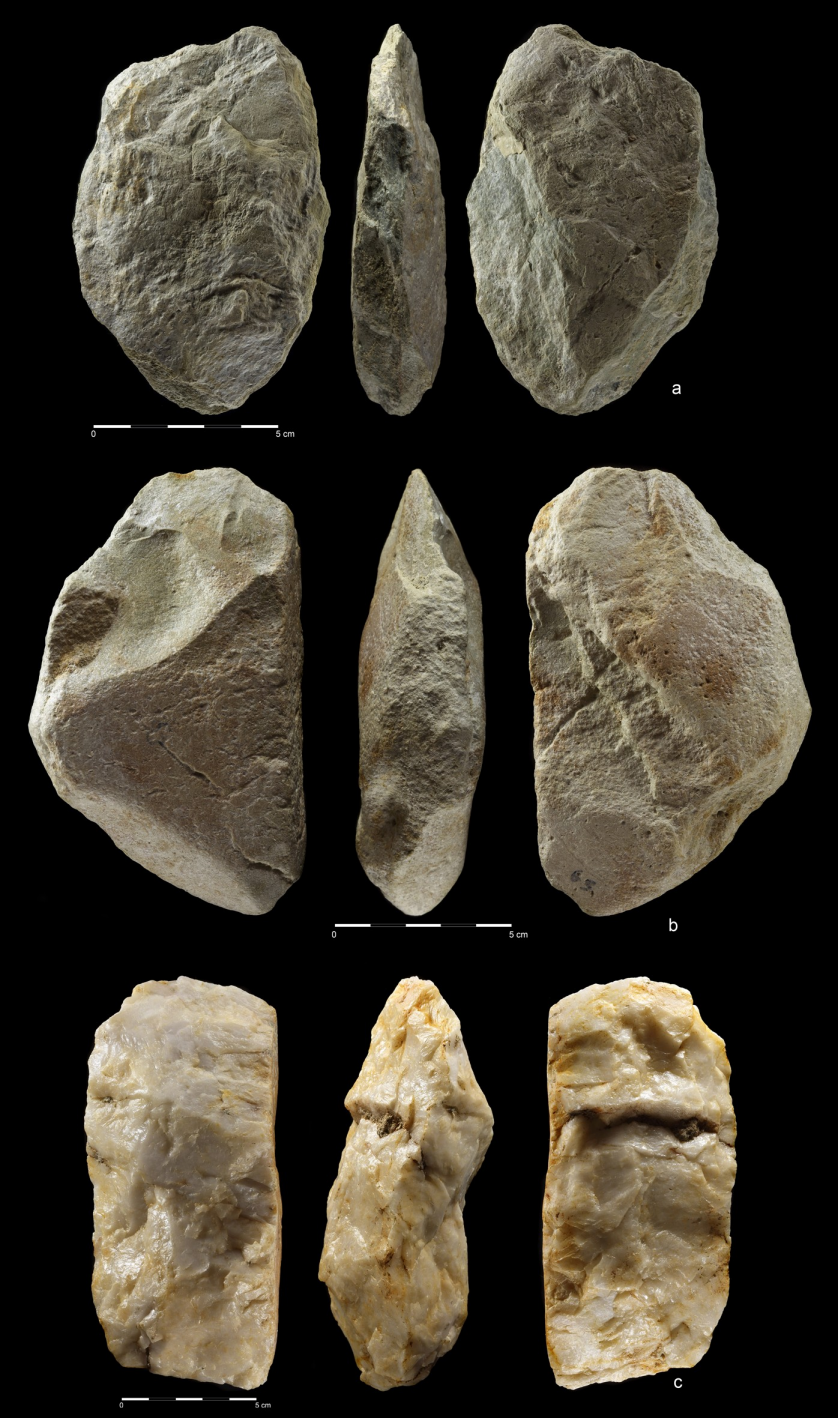
Cleavers. Rhyolite (a,b), quartzite (c). (a) art. no. 460, (b) art. no. 63, (c) art. no. 114.

**Fig 18:**
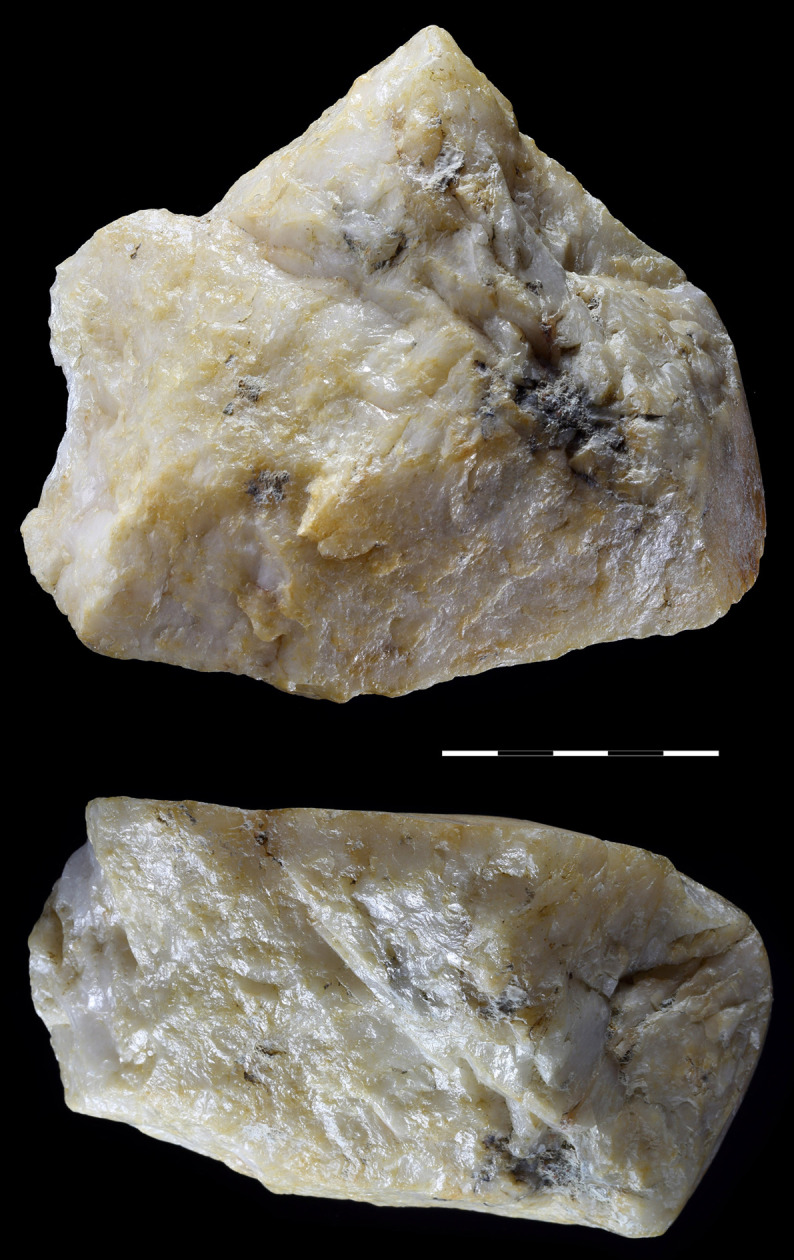
Unifacial pointed chopper. Quartzite. art. no. 100.

**Fig 19:**
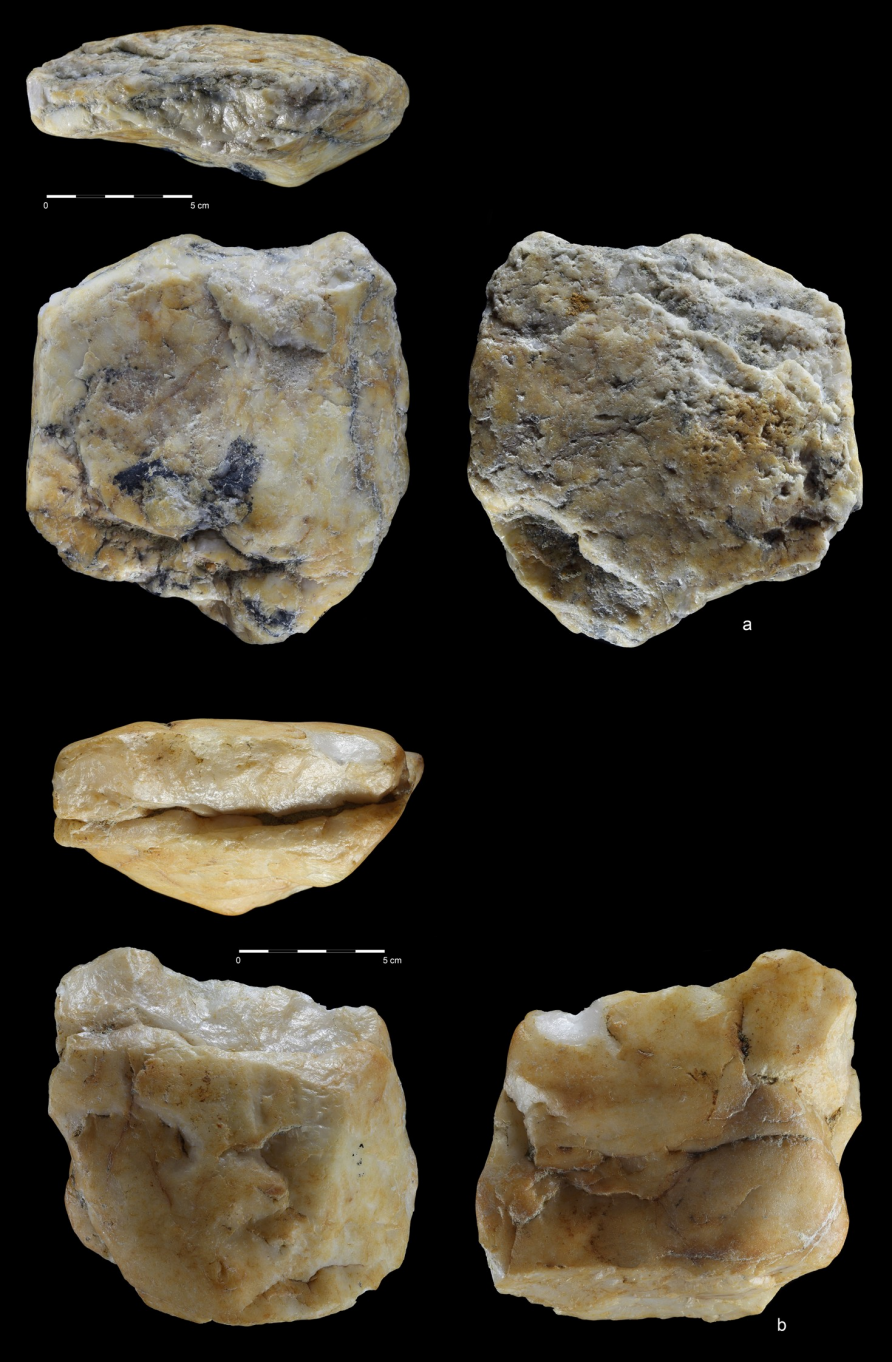
Choppers. Quartzite. (a) art. no. 521, (b) art. no. 522a.

**Fig 20:**
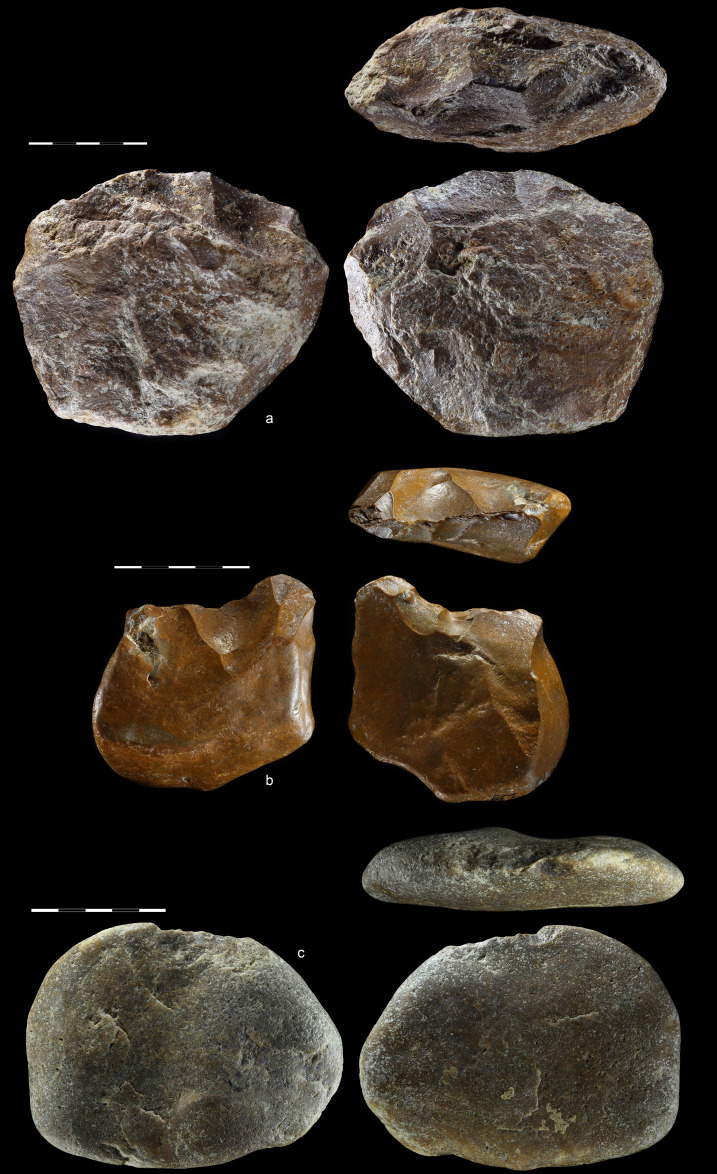
Chopping tools. Quartzite (a,c), chert (b); **(**a) art. no. 29, (b) art. no. 239, (c) art. no. 511.

The assemblage of bifacial tools from EDAR 7 numbers 37 artefacts, including 27 hand-axes (Figs 10–14, 15A and 15B), 9 cleavers (Figs 15C, 16 and 17)). The group of products defined as Large Cutting Tools (LCT) is complemented by pebble tools, i.e. 3 choppers (Figs 18 and 19) and 5 chopping tools (Fig 20) (Table 4). Some of the hand-axes could possibly be also included into a pebble tool category, i.e. specimen in Figs 11A and 13C could be classified as lateral-distal bifacial-choppers known from much older inventories i.e. from the Melka Kunture site in Ethiopia [82]. Most of the hand-axes and cleavers and all the choppers were preserved intact or with only a small breakage of the tip (S10 Table). Only four tools display a small degree of abrasion (2 choppers and 2 hand-axes), while for 40 out of 44 were determined as abraded.

**Table 4:** Large cutting tools.

LCT type / Raw material	Quartzite	Rhyolite	Chert	n Total	%	Weight (g)	%
**Biface preform**	4	-	-	4	9,09	1778	6,66
**Hand-axe**	13	6	-	19	43,18	10557	39,56
**Hand-axe with "cleaver like" edge**	2	2	-	4	9,09	2341	8,77
**Cleaver**	3	6	-	9	20,45	4343	16,28
**Chopper**	3	-	-	3	6,83	3725	13,96
**Chopping tool**	3	1	1	5	11,36	3940	14,77
**Total**	28	15	1	44	100,00	26684	100,00

Frequencies, weights and raw materials.

In terms of raw material, the frequency of rhyolite is conspicuously larger among LCTs (over 18%) than in the remaining categories of the assemblage. Preference for this raw material is especially marked among cleavers: 66% of them were made of rhyolite, as well as two forms defined as hand-axes with “cleaver-like” edge (the crosswise edge smaller than half of the product’s width) [72]. One of the choppers was made from chert—outcrops of this raw material are absent from the immediate surroundings of the site.

The most frequently used material to produce nearly 65% of bifacial tools were big pebbles and cobbles of naturally flat shape. Specimens based on giant flakes constitute an important contribution in the assemblage. One of them has two ventral sides, which testifies to the use of Kombewa flakes (Fig 13B). Such behaviour might be underrepresented due to later modifications and surface deterioration obscuring the original blanks. Use of the Kombewa method was also noticed at EDAR 6, an Acheulean site located ~2 km from EDAR 7.

The cross-sections of the bifaces indicate a strong tendency to maintain flat-convex, trapezoidal or lenticular forms. The final shape may have resulted from either the selection of raw material blocks of preferred shapes and the exploitation of natural surfaces, or the use of flake blanks.

The sizes of hand-axes and cleavers are presented in S11 Table. The width is the most variable dimension, ranging from 51 to 179 mm. Some variation is also visible in length. Cleavers are slightly smaller than hand-axes, which may result from the fact that some of the former were based on flakes, while the latter on quartzite blocks of considerable sizes. Considerable variation of sizes is evident among choppers and chopping tools: minimum and maximum values of all four features are spread over a wide range. Medians and mean values reach approximate results, which indicates the absence of outliers.

Differences are seen in the general picture of use of both surfaces in the reduction of the bifacial forms from EDAR 7. The mean negative count for face one was 1.71 greater than for face two (S9 Fig); this is furthermore reflected by the higher maximum value and median for face one. As far as the frequency of occurrence of negatives on surfaces is concerned, there is a clear tendency for reduction of the distal part and medial lateral part on face one, while face two is characterised by a great number of negatives in the distal part. Overall, EDAR 7 bifaces display considerable surface modification during production. Nineteen have less than 50% of natural surface on face one; 17 on face two. To some extent, this results from the use of flake blanks in LCT manufacture.

Thinning retouch and bifacial edge retouch were rarely implemented during the production and repair of LCTs. The former was observed on face one of five artefacts and on face two in three artefacts. The latter was infrequent as well and occurred on four tools (S9 Fig).

#### Bifacial tool production methods

Three significant production methods of bifacial tools are present in the EDAR 7 assemblage (S10 Fig). The first one focused on making hand-axes and cleavers out of cobbles and chunks. In the case of cleavers, production started with a flat cobble and was focused on bifacial shaping with blows perpendicular to the main axis; these blows tended to be invasive and removed most of natural surface (Fig 17B and 17C). One example of treating the natural surface as a striking platform in early shaping was observed; this was somewhat similar to unidirectional core reduction. Preparation of a working edge by either detaching one or two perpendicular flakes or using a series of bifacial parallel blows was the next stage in that method.

In the case of hand-axes, the strategy was somewhat different and aimed at preparing and shaping the tip and both sides without (or with minimal) modification of the base (Figs 10B, 11B, 12A, 14C and 15A). In this case, the blow scars have a bifacial and semi-circular characters. Most of the hand-axes made using this method have a high proportion of natural surface on both faces, probably a result of using suitable, flat cobbles and chunks with plano-convex, lenticular and trapezoid cross section.

The second method of producing hand-axes from cobble can be described as a classical, circular bifacial approach with three consecutive stages of reduction (Figs 10A,10C, 11A,11D, 12B,12C, 13A, 14A and 14B and 15B). The first stage is associated with general block shaping; the second was aimed at roughing-out and shaping the tip and sides. Small scars of thinning and bifacial retouch resulting from the last stage of *faconnage* are located near tool edges. Such hand-axes have almost no natural surfaces on both faces, are symmetrical and have a regular, cordiform, limande or oval shape. The last two stages of *faconnage* of hand-axes with cleaver-like edge were slightly different. The working edge was created by either two notch negatives or tip reduction using small perpendicular flakes.

The third method was based on using giant flake blanks (Figs 11C, 13C, 16A–16C). It is confirmed not only by flake LCTs, but also by two giant cores (Fig 6) and a few large flakes (Fig 9A–9C). Blank roughing-out is similar for both cleavers and hand-axes and it was focused mostly on dorsal side reduction. Most ventral side scars can be linked with butt thinning or with bifacial edge shaping. Upper face scar directions were the same as in the case of the cobble/chunk-based methods: perpendicular on cleavers and circular on hand-axes. The cutting edge of cleavers and cleaver-like hand-axes was created by detaching notch-like blows located on both sides of the product. This method is also known from early Acheulean assemblages of Eastern Africa [83].

One hand-axe was made on a Kombewa flake (Fig 13B). Faces and edges of this artefact were reshaped only slightly; natural convexities and sharp edges of the blank were used instead. Thus, the reduction was mostly focused on removing the bulbs. As mentioned before, the Kombewa method could have been used more extensively, but it was later obscured by heavy modification and abrasion of artefact surface.

Two main factors had a significant impact on using different strategies of production as a well differently shaped degree of LCT’s in the production process. The first concerns the transmission of knowledge within a group and also a skill level of individuals. Mastering “knowledge” (*connaissance*) as well as obtaining an appropriate level of “know-how” (*savoir-faire*) distinguished experts from novice knappers [84]. Expert knappers were better at planning and controlling the reduction process, including the application of appropriate solutions when mistakes were made or they were faced with raw material limitations [85]. The hypothesis of different skill levels could be confirmed by the presence of bifacial forms made of fine-grained rhyolite (see Figs 11C, 12A and 12C, 13A and 13C and 16) and quartzite (see Figs 10A, 11A, 12B, 14B, 14C, 15A and 15B) with strong control of both faces and regular and deep removals, which could be a result of a high skill of knappers. On the other hand the forms displaying a small degree of processing the face and the presence of numerous hinges point to an inefficient skill level.

The second factor is connected with the adaptation of production methods to raw material. To verify the possibility of using direct percussion technique with a hard hammer in the production of LCT’s and reduction of giant cores, we conducted a short experiment with local raw materials which we gathered from the area close to the location of EDAR sites (S11 Fig). Two knappers with advanced skill level were involved in the knapping activity (M.E and G.M) (S11A and S11B Fig). Experiments showed that three kinds of raw material (fine-grained rhyolite, coarse grained rhyolite and quartzite) have varied fracture properties, which is indicative of different degrees of control in the detaching process (S11D–S11F Fig). Fine-grained rhyolite has the best fracture properties, which allowed better control of flaking angle and surface shape at each stage of shaping (S11E Fig). The quartzite raw material is characterised by good fracture but it required grater control skills—use of a hammerstone which was too heavy caused severe chipping and microcracks in the structure of raw material, which prevented continuation of shaping (S11A–S11D Fig). The lowest fracture properties are characterised by coarse-grained rhyolite, which causes presence of numerous hinges and difficulty in decortications and detaching overshot flakes (S11F Fig). A large number of hinged scares on faces and asymmetrical shape of LCT’s is visible in the assemblage from EDAR7—bifacial tools made from coarse-grained rhyolite (Figs 11B, 15C and 16A).

#### Retouched tools

The retouched tools, made on flakes and sometimes chunks, constitute 67,4% of the tool category; the remainder are LCTs and one hammer stone (Table 5 and Figs 21–24 and S12–S20). Such ubiquity of flake tools, some of which are very small, suggests differentiation of activities that took place in EDAR 7 area, and a functional complexity of the assemblage.

**Fig 21:**
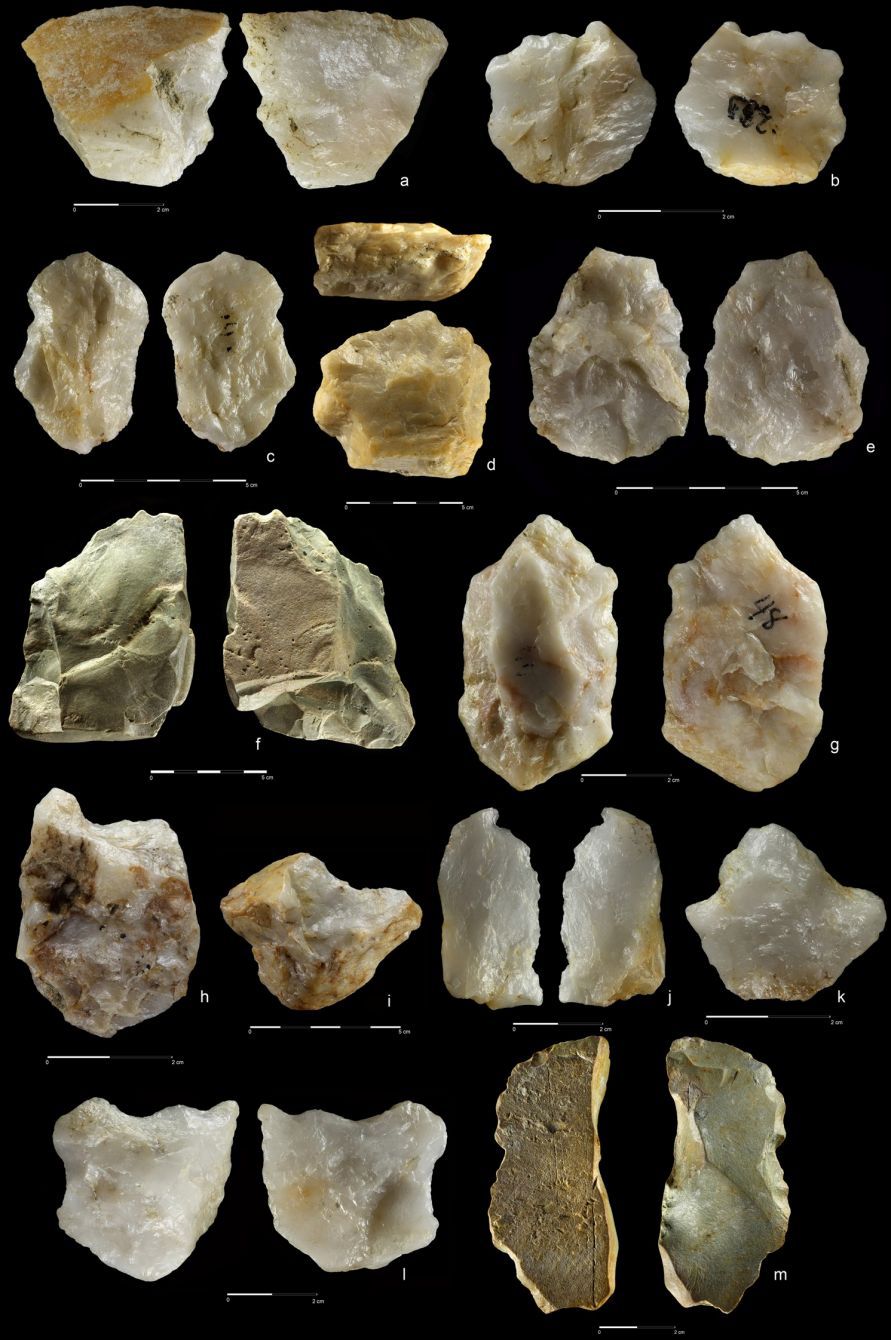
Denticulate and notches. Quartzite (a-e, g-l), rhyolite (f, m). Denticulate: (a) art. no. 418, (b) art. no. 237, (c) art. no. 447, (d) art. no. 139, (e) art. no. 462, (f) art. no. 13 and notches: (g) art. no. 48, (h) art. no. 374, (i) art. no. 23, (j) art. no. 69, (k) art. no. 242, (l) art. no. 340, (m) art. no. 213.

**Fig 22:**
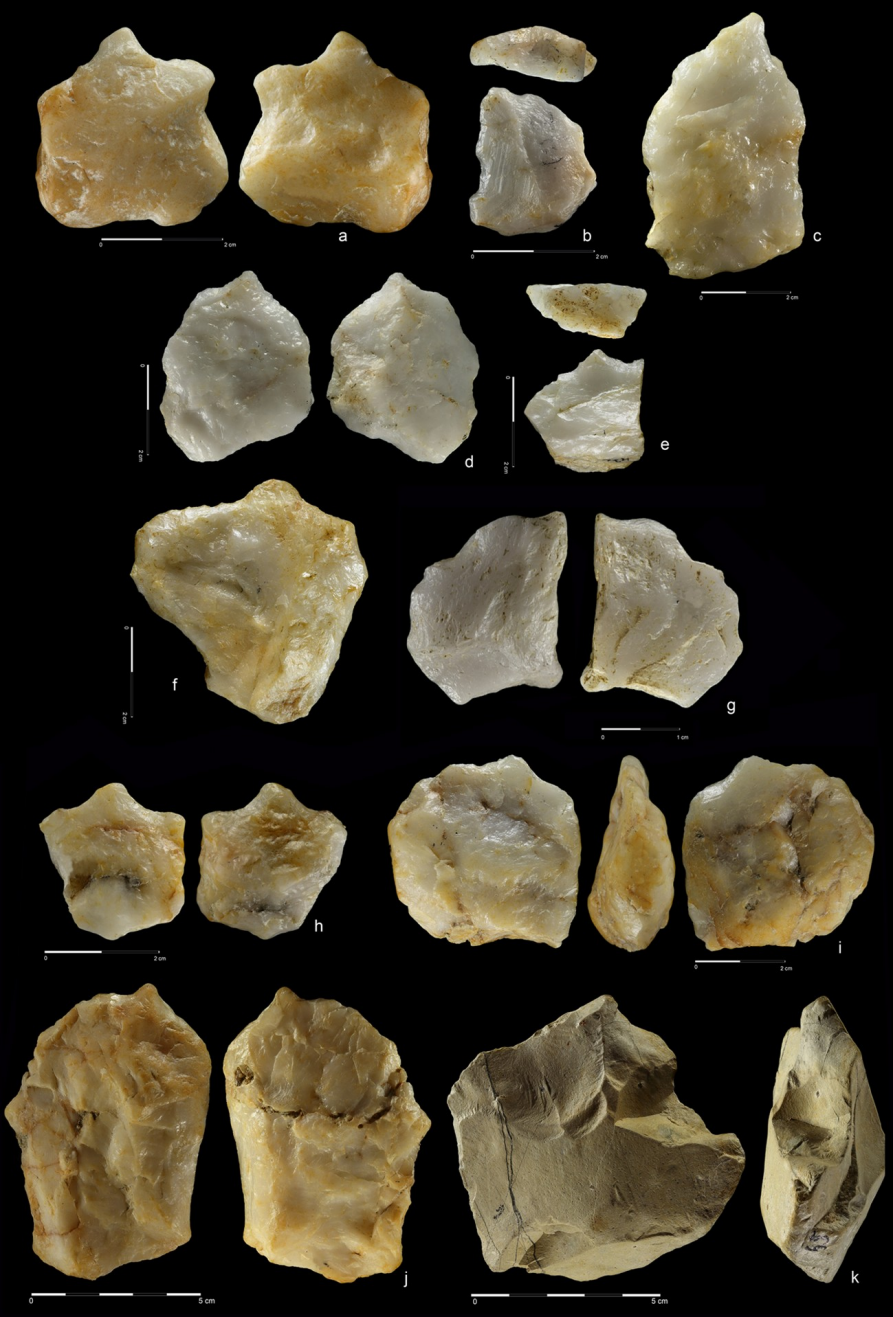
Perforators. Quartzite (a-k), and rhyolite (i); (a) art. no. 85, (b) art. no. 142, (c) art. no. 292, (d) art. no. 327, (f) art. no. 472, (g) no number, (h) art. no. S8, (i) art. no. S29, (j) art. no. 459, (k) art. no. S52, (l) art. no. 65.

**Fig 23:**
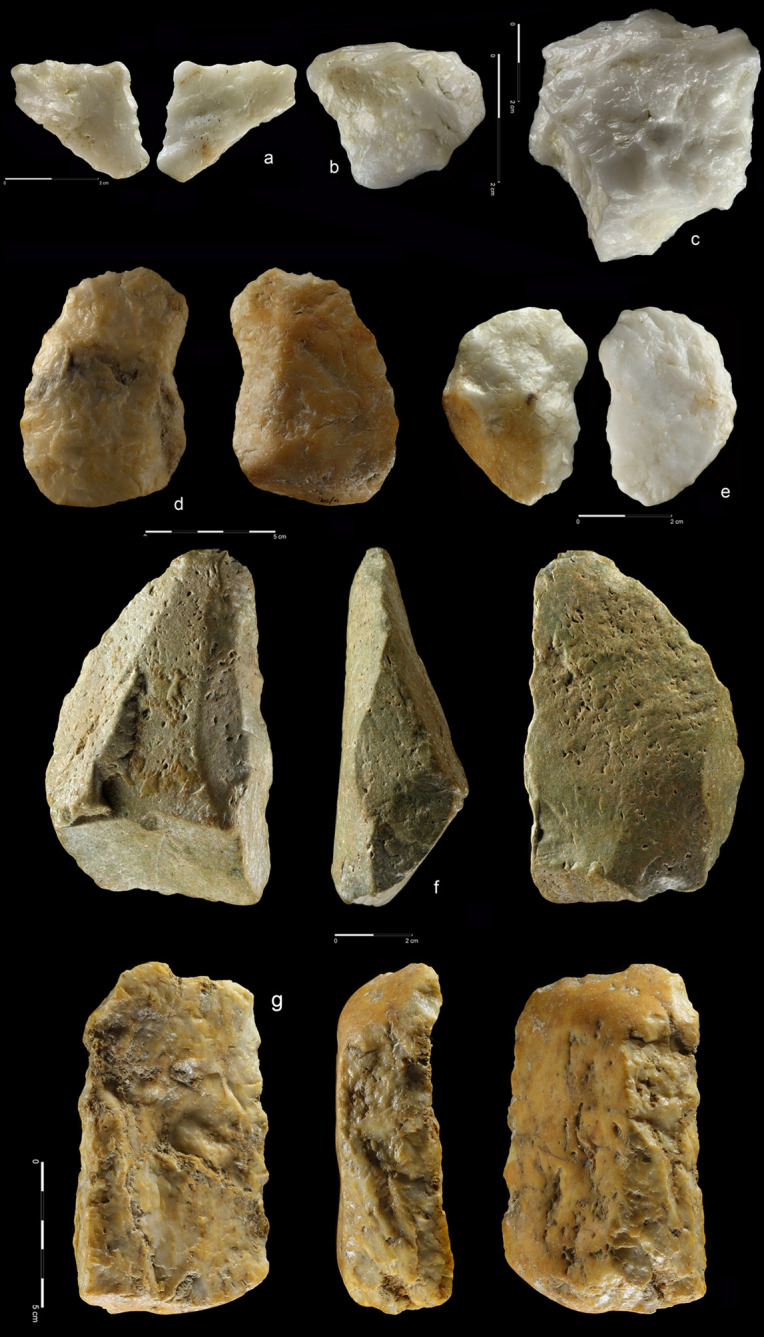
Endscrapers and sidescrapers. Quartzite (a–e, g) and rhyolite (f). Endscrapers: (a) art. no. 357, (b) art. no. 42, (c) art. no. 67 and sidescrapers: (d) art. no. 217, (e) art. no. 40, (f) art. no. 247, (g) art. no. 525.

**Fig 24:**
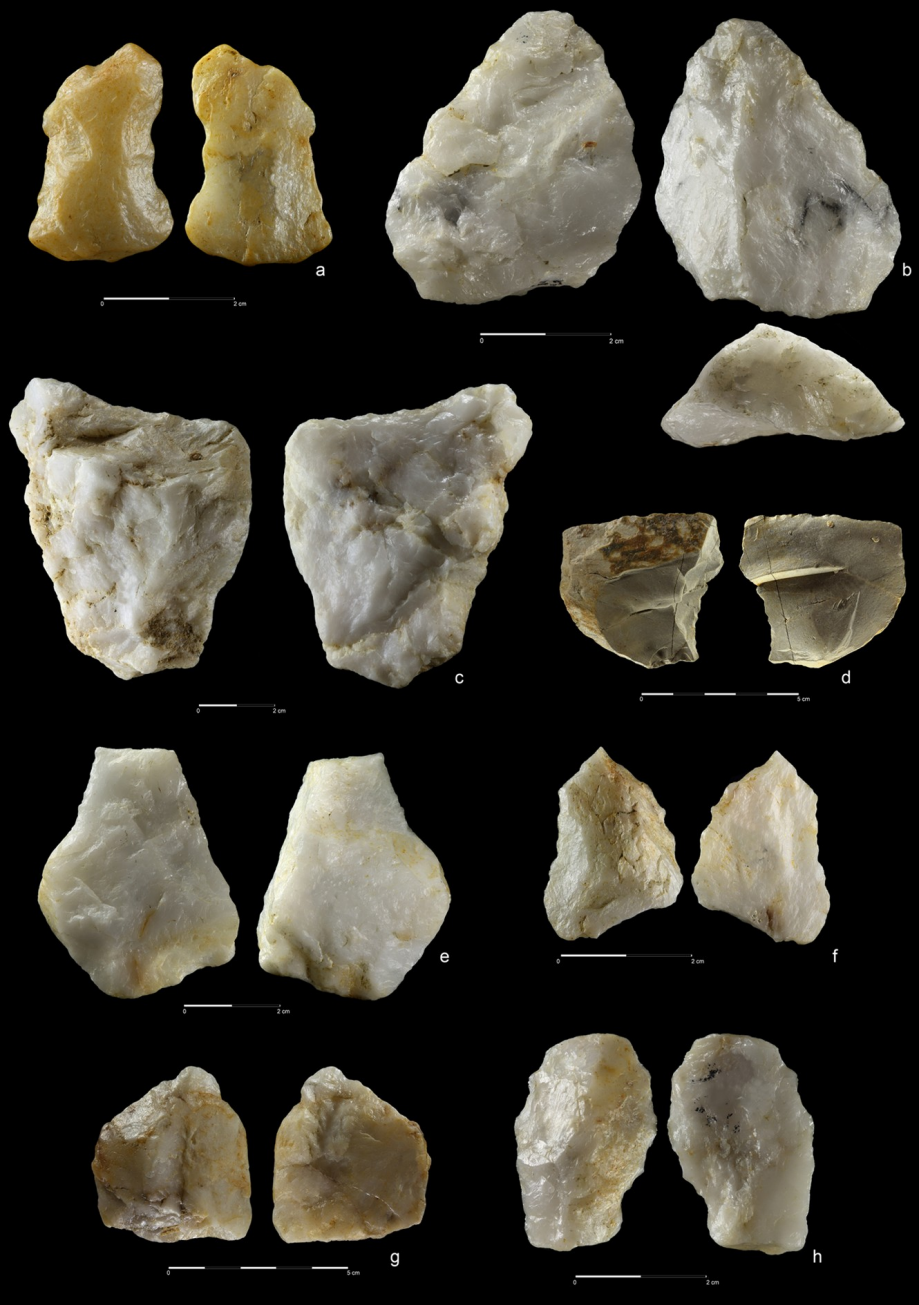
Composite tools and retouched flakes. Quartzite (a–c, e-h) and rhyolite (d). Composite tools: (a) art. no. 59, (b) art. no. 523; and retouched flakes: (c) art. no. 36, (d) art. no. 125, (e) art. no. 21, (f) art. no. 300, (g) art. no. 272, (h) art. no. 226.

**Table 5:** Retouched flake tools type frequencies.

Type of tool	Raw material			
	Quartzite		Rhyolite	
	n	%	n	%
**Denticulate**	13	13,98	4	4,30
**Notch**	13	13,98	1	1,08
**Perforator**	10	10,75	1	1,08
**Sidescraper**	11	11,83	1	1,08
**Endscraper**	7	7,53	-	-
**Composite tool**	4	4,30	-	-
**Retouched flake**	19	20,43	9	9,66
**Total**	77	82,8	16	17,2

The state of preservation in this group shows that fewer than 10% of artefacts are fragmented. Core reduction stage was not a crucial factor for blank selection: non-cortical (52%) and cortical ones (47%) were used. Butts of more than 40% of the blanks were natural; another 41% were plain with only marginal participation of dihedral, linear, and punctiform types. Some of the tools, especially perforators (Figs 22 and S15) or endscrapers (Figs 23 and S17) were rather diminutive, with lengths ~ 20 mm and width not exceeding 10 mm (S12 Table). Such small size could be a premise on which the possibility of hafting can be considered.

Both raw materials were used for the manufacture of flake tools. As in the entire assemblage, quartzite dominates over rhyolite. With the exception of quartzite-only endscrapers, all tool categories were made of both raw materials. Retouched flakes were the most common tool type. Denticulate, notches, perforators and sidescrapers were found in equal proportions, each type making up more than 10% of the tool group.

The most frequent retouch type is notch/denticulate, usually on the dorsal side of the flake. Straight delineation predominates, while concave type occurs less frequently. The distal part and the right edge are the most commonly retouched flake zones. The mean length of retouched edge is ~40 mm (S13 Table).

#### Stone slab

**Fig 25:**
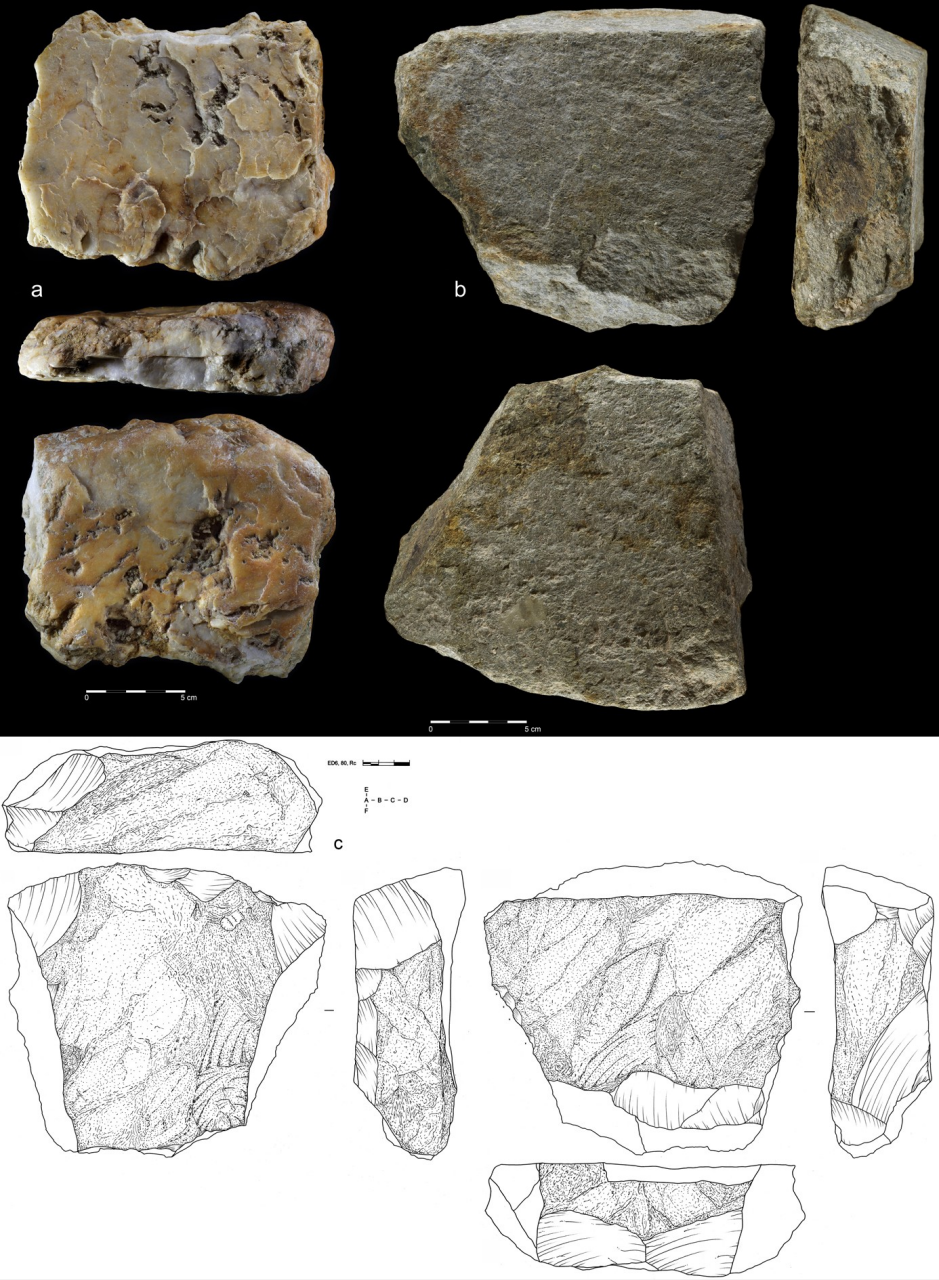
Worked stone slabs. EDAR 7 (a) and EDAR 6 (b, c). Quartzite (a) and rhyolite (b, c). (a) art. no. 513), (b, c) art. no. II/8.

The Acheulean artefacts were accompanied by a large, flat, rectangular quartzite block (151×140×44 mm, weight 1,57 kg) trimmed by several blows to the edges (Fig 25A). A similar block, but made of rhyolite and much bigger (220×180×75 mm, weight 4,25 kg) was also found at the nearby Acheulean site EDAR 6 (Fig 25B and 25C) [32]. Such blocks are known among others from the Sangoan levels at the Sai Island site and are interpreted as grinding stones [86]. Basalt blocks of this kind have elsewhere been used primarily as an anvil, a passive percussion tool e.g. at Gesher Benot Ya’aqov [72, 87].

### Geometric morphometric analyses of hand-axes

Results of the principal component analysis (PCA) (S14 Table) indicate that the first 10 components describe 96.1 of the variance of the whole population, with the first two components accounting for 46.8% and 29.3% respectively. PCA results in the form of the scatter plot were presented on the basis of the first two components due to the fact that when combined they account for over 75% of variance.

**Fig 26:**
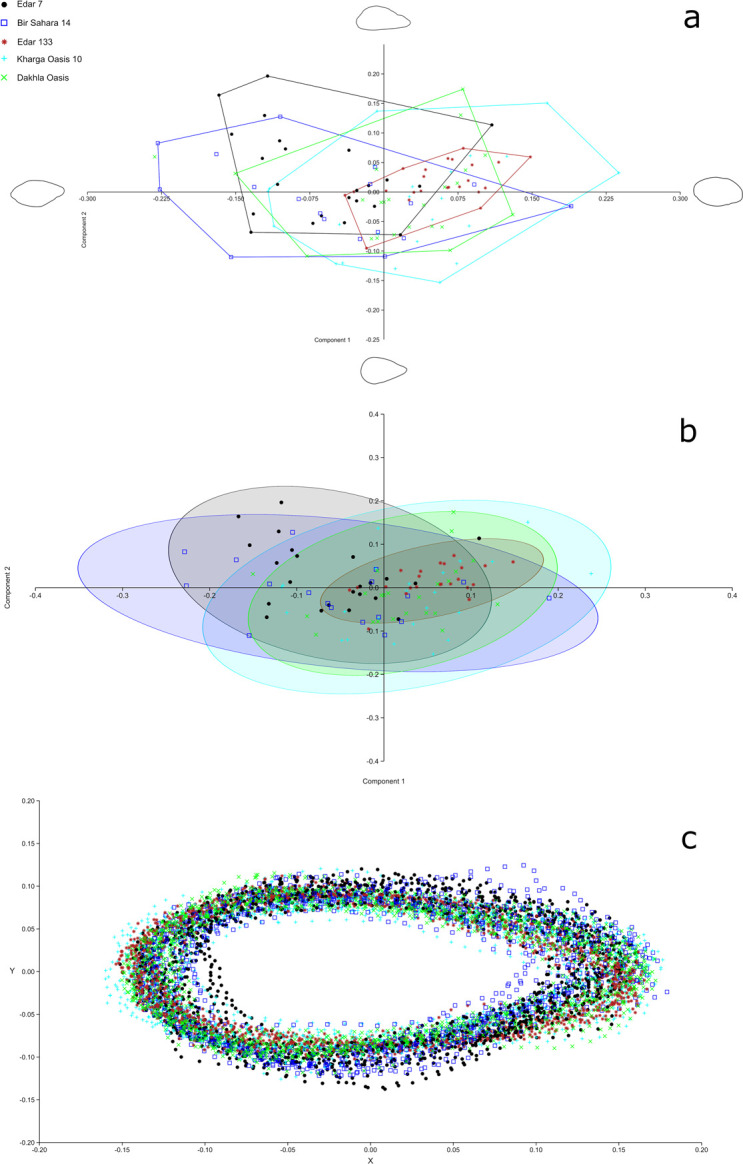
Geometric morphometric analysis results. (a) PCA results for 1 and 2 components; (b) PCA results with outlined ellipses of 95% confidence interval for each group of artefacts; (c) procrustes superimposition of hand-axes outlines.

The Thin-Plate Spline Deformations analysis showed diversification of artefacts’ shapes situated in relation to the axes of two main components. To determine the shapes of products, we resorted to the classification proposed by F. Bordes [88]. Two irregular subcordiform (up) and cordiform (down) LCTs are visible on the axis of the first component, while the second component is connected with the elongated (left) limandes shape similar to the cleaver-like edge and the shape approximating an oval (right). Location of the objects from assemblage EDAR 7 displays three tendencies in acquiring particular shapes: approximating elongated limandes and irregular subcordiform and regular limandes. A tendency similar to EDAR 7 is seen in assemblage Bir Sahara 14, yet in this case it differs in the location of the observed extreme objects and increased frequency of observed objects of the limandes shape. Samples from Dakhla Oasis and Kharga Oasis show a great similarity in the dispersion of data, with a strong tendency to assume limandes and elongated limandes shapes. The PCA analysis yielded totally different results for assemblage EDAR 133. There the assemblage is characterised by strong standardisation of product shapes, which is seen in very small spread of observations (Fig 26A). The LCT’s found at EDAR 133 mainly display irregular subcordiform shape and the shape approximating an oval.

Mardia’s kurtosis test showed absence of normality of distribution for all variables (p = 2.414E-125). Because the assumption of normality of distribution was not met, we used the MANOVA test with Pillai’s trace. The results of the MANOVA and PERMANOVA tests (S15 Table) did not reach the required value, which unequivocally indicates the rejection of the zero hypothesis about absence of diversification between the assemblages subject to the analysis. The results of similarity in pairs implemented in the PERMANOVA test indicate a similarity between assemblage EDAR 7 and Bir Sahara and a considerable similarity between Dakhla Oasis and Kharga Oasis (S16 Table). Results exceeding the assumed significance (p>0.05) are also visible when comparing EDAR 133 and Dakhla Oasis; yet this result should be treated with extreme caution, due to a low value exceeding significance (p = 0.0699).

### Spatial arrangement of the assemblage

**Fig 27:**
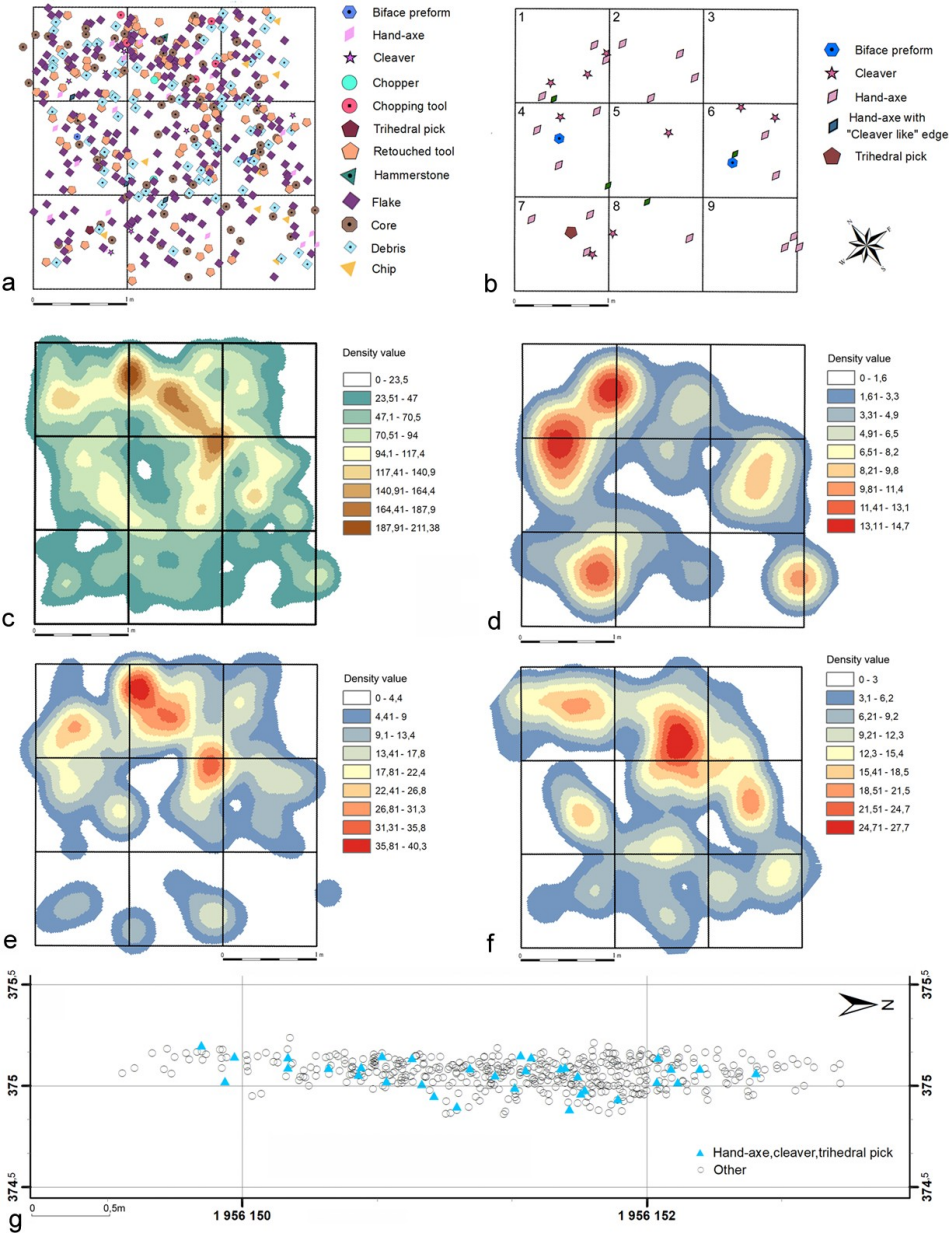
EDAR 7. Spatial artefact distribution and density maps. (a) general distribution, (b) distribution of the bifacial component, (c) density map of artefacts, (d) density map of bifacial component, (e) density map of retouched tools, (f) density map of cores, (g) vertical distribution of artefacts. Metres numbering is given in (b).

The artefact positioning indicates that the excavated area represents remains of an occupational surface with stone knapping activity. Analysis of the spatial distribution of lithics does not allow the identification of separate functional zones within the trench, although several clusters of stone artefact categories could be observed (Fig 27). This contrasts with the other Acheulean encampment remains from Sudan–Arkin 8, where traces of some of the earliest known domestic structures in Nubia have been documented. Arkin 8 consists of a main concentration of finds, several subconcentrations, cairns and possibly wind-shelters [28]. It was interpreted as a “prehistoric camp, with its living floor”. The area documented at EDAR 7 is much smaller than at Arkin 8 (9 m^2^ and 64 m^2^ respectively) and it is clear that only a small part of the site has been excavated.

The majority of artefacts are located in the northern zone of the trench, especially within metre no. 2, which contained 20% of the whole assemblage. However, the density maps show that the individual categories of stone artefacts concentrate in slightly different areas of the excavation. The bifacial component was most frequently recorded in the northern part within metres no. 1 and 4. Smaller clusters were also identified near the western and eastern margins of the trench. Retouched tools are located most frequently in the north-eastern part. In the case of cores, several clusters can be seen. The highest density of this type was recorded within metres no. 2 and 5. Smaller core clusters formed within metres no. 1, 4 and 6. The pebble component (5 chopping tools and 3 choppers) constituted a set too small to form any conceivable clusters.

### Use wear analysis

The study of tools from EDAR 7 yielded results that are interesting both from the point of view of preservation and interpretation of traces. Macro traces suggesting possible wear were detected on the edges of all 15 artefacts. They included heavy rounding and numerous scars with step and feather endings. In 11 cases, more than one edge had macro traces suggesting use (Table 6). The remaining 4 had natural surfaces which could have been used as a back for handling the tool.

**Table 6:** Summary of use-wear traces interpretation of analysed artefacts.

Art. Number	Movement			Material					
	Scraping	Cutting	Perforating	Soft	Hard	Hide	Wood	Bone	Not identified
**S8**	+	-	-	+	-	-	-	-	-
**S9**	-	+	-	-	-	-	-	+	-
**S29**	-	-	+?	-	-	-	-	-	+
**69**	-	+	-	-	-	-	-	-	+
**142**	-	-	+	-	+	-	-	-	-
**340**	-	+	+	-	-	+	-	-	-
**357**	+	+	+?	-	-	-	+	-	-

Direction of tool movement was identified based on linear features [49, 57]. Three distinct types of movements could be recognised on the tools: cutting, scraping and perforating. In most cases, the type of movement was consistent with the formal type of the tool. Each of the analysed artefacts tells a slightly different story of use and thus deserves a short, separate description.

**Fig 28:**
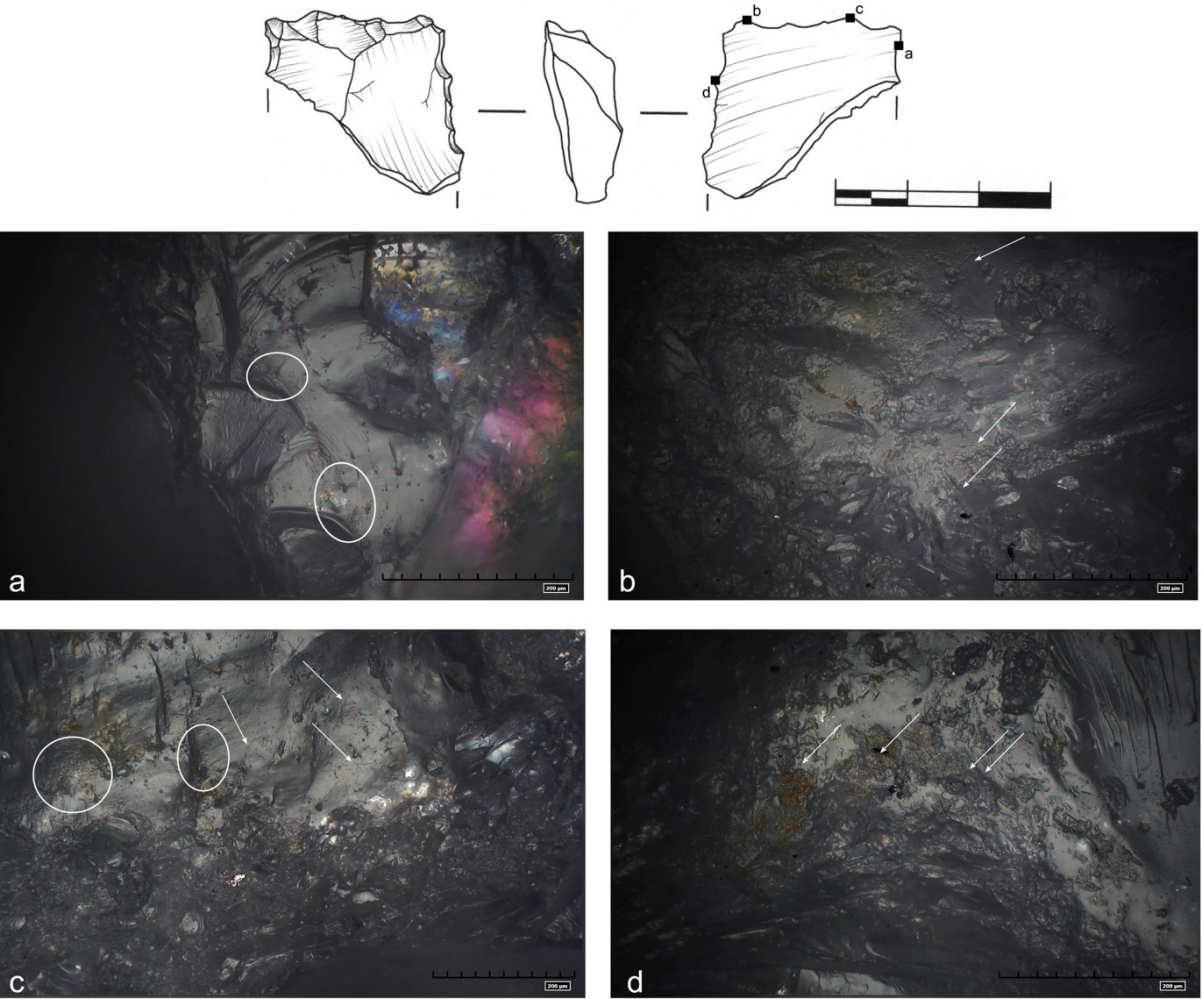
Use wear interpreted as traces of wood working. Rough and domed polish. Numerous impact pits (ellipses), grooves and striations (marked with arrows). Two directions of movement can be distinguished: regular straight-sided striations parallel and perpendicular to the edge (art. no 357).

A fragment of a tool (art. 357) with retouched edges that was used for working wood is an interesting case. All edges of the artefact were used and display different stages of development of the same type of traces. Analysis revealed a smoothed surface covered with rough, domed and matt polish. A series of small, overlapping scars are apparent at low magnification. Wear of the left edge was least developed–irregular rounding and fractures were first to appear together with small concentrations of impact pits (marked with ellipse) (Fig 28A). Stronger traces were noticed on the proximal part of the tool. Linear features are crisscrossing behind the edge. The direction of grooves (marked with arrows) suggests more than one mode of movement of the tool: cutting and scraping (Fig 28B and 28C). In the distal part of the left edge, a well-flattened surface with large impact pits, grooves and striations perpendicular to the edge of the tool was located. This part of the tool was used for scraping (Fig 28D).

**Fig 29:**
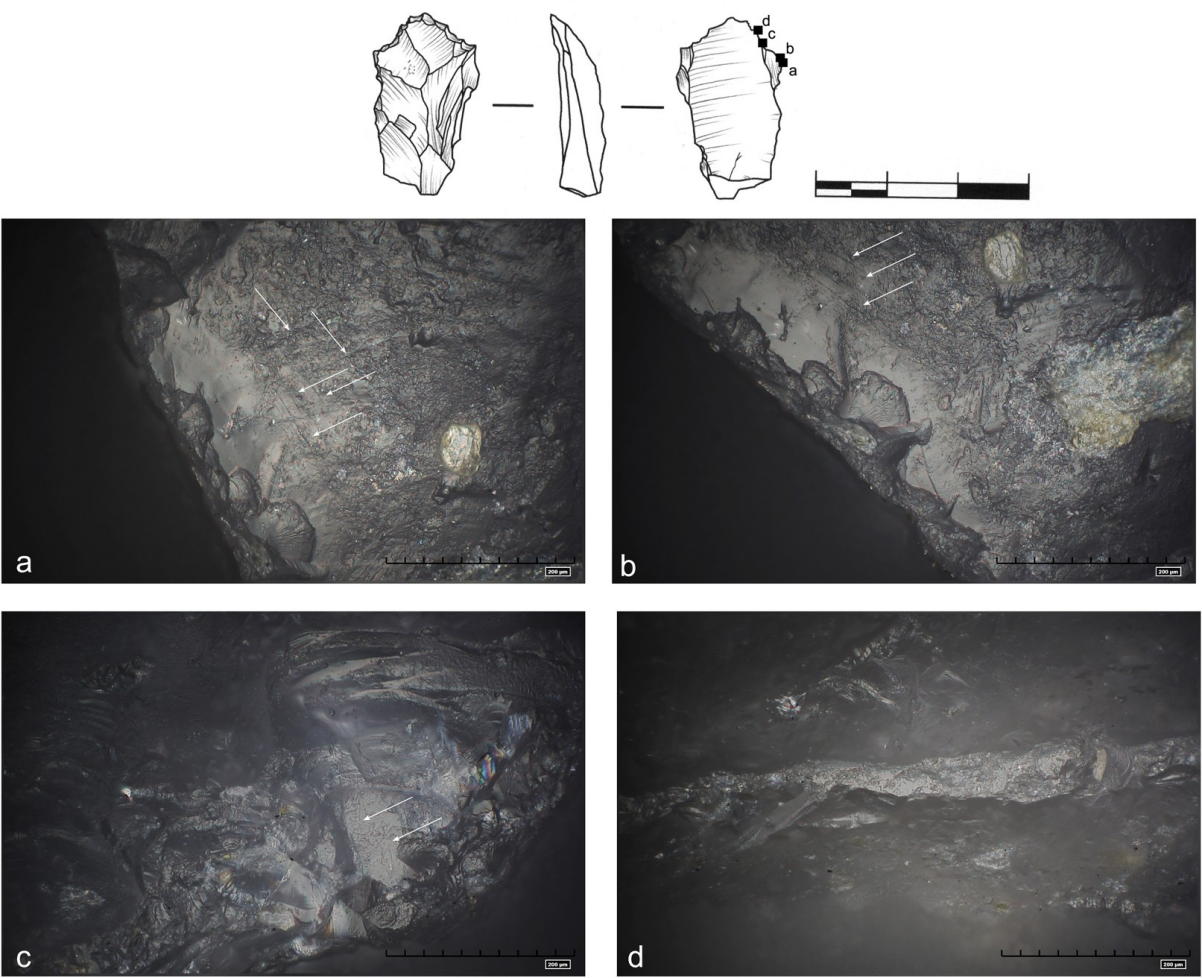
Use wear on a tool used for bone cutting/engraving. Well developed, bright polish. Regular sleeks parallel to the edge with some crisscrossing striations (marked with arrows). A series of small cracks in the upper left part of the photo a (art. no S9).

Another example of distinctive polish was discovered on the surface of a small endscraper (art. no S9). Surprisingly, the tool was not used for scraping, but cutting and perhaps engraving bones. Heavy traces were discovered along the left edge of the tool (Fig 29). Very little rounding could be observed with the naked eye, although series of visible, uneven scars were apparent. Well developed, highly visible, well flattened and shiny bright polish concentrated on the very edge of the tool, but this pattern did not continue onto the surface of the endscraper. Regular sleeks were found, going parallel to the edge (Fig 29A, 29B and 29D), as well as several perpendicular striations (Fig 29A and 29C).

**Fig 30:**
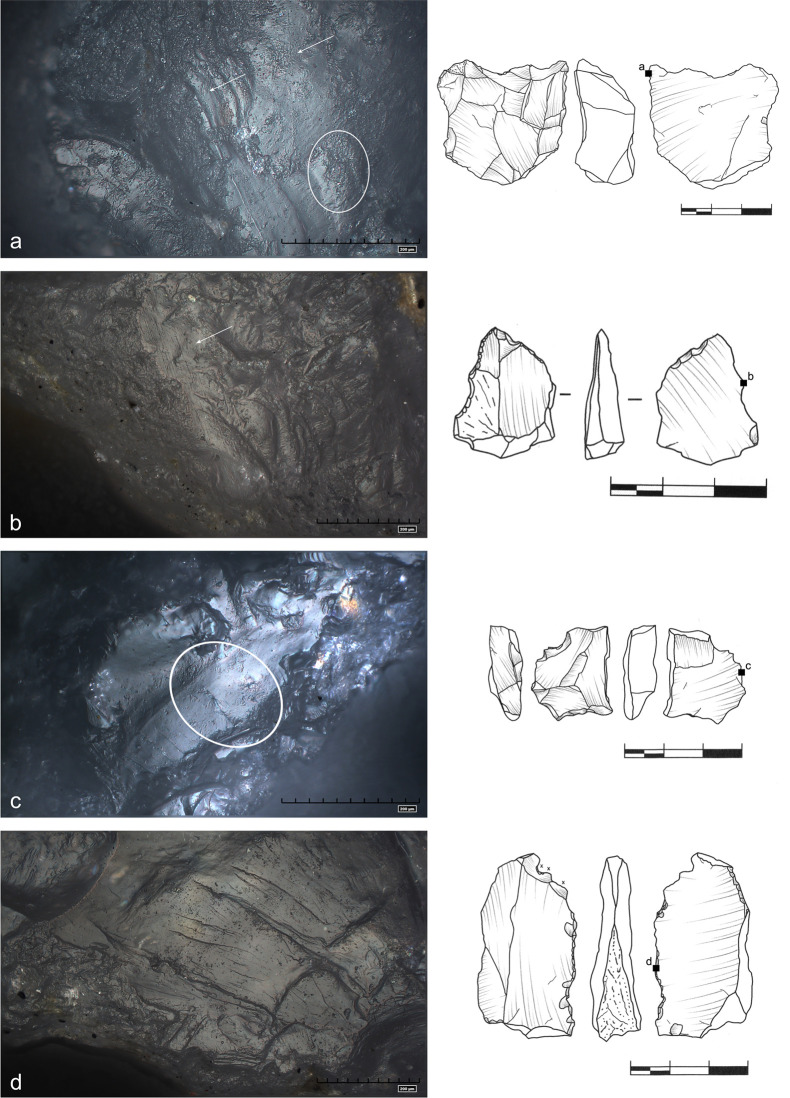
Examples of other observed traces, more difficult to interpret. **(**a) irregular, discontinuous striations perpendicular to the edge (marked with arrows), numerous impact pits (marked with ellipse) and cracks of the ridge (art. no 340); (b) deep sleeks running regularly perpendicular to the edge; slightly shiny polish on the protruding part of the tool (art. no 142); (c) weakly developed use wear, some shallow sleeks going perpendicular to the edge (art. no S8); (d) Traces partially destroyed by post depositional wear–numerous scattered impact pits. Visible crushing of the edge and micro scars on some ridges (art. no 69).

Two artefacts bore traces of both perforating and cutting (art. 340, 142). Both had a small, rather blunt tip, shaped with series of strikes to the edge. Extreme rounding of the edges and all protruding parts, especially the tips was visible to the naked eye Unevenly distributed small scars with step and feather ending could be also noticed. At the microscale, some differences between the two artefacts could be easily detected. On one (Fig 30A), polish appears in irregular, matt spots. There are also numerous cracks and impact pits forming linear features running perpendicular, around the perforator, and parallel along one of the edges of the tool. The other revealed regular, deep sleeks perpendicular to the edge (Fig 30B). The material worked was impossible to determine precisely due to heavy post depositional abrasion (Fig 30C). It is possible that the first of the artefacts was used on softer material, whereas the second on something harder.

Directions of traces, but not the specific material worked, were identified for two more artefacts. On one, some clues could be deducted from macro traces (art. S8). The left edge of the tool was visibly rounded, with many overlapping chips of step and feather ending. The remaining edges appeared somewhat fresher. Further traces were observable in greater magnification only: spots of rough polish on the protruding part of the tool; several scattered impact pits and some irregular striations perpendicular to the edge (Fig 30C). Such traces could imply use on some kind of soft material, maybe hide or meat [89, 90], but post-depositional traces make this interpretation difficult. A small knife showed similar wear (art. no 69). One of the edges, heavily crushed, bore all the traces. This edge was opposed by a natural surface that could be used for handling the artefact. Most of the micro traces were obscured by post depositional wear. It was only established that some patches of more regular, bright polish sleeks going parallel to the edge could signify that this tool was used for cutting (Fig 30D).

**Fig 31:**
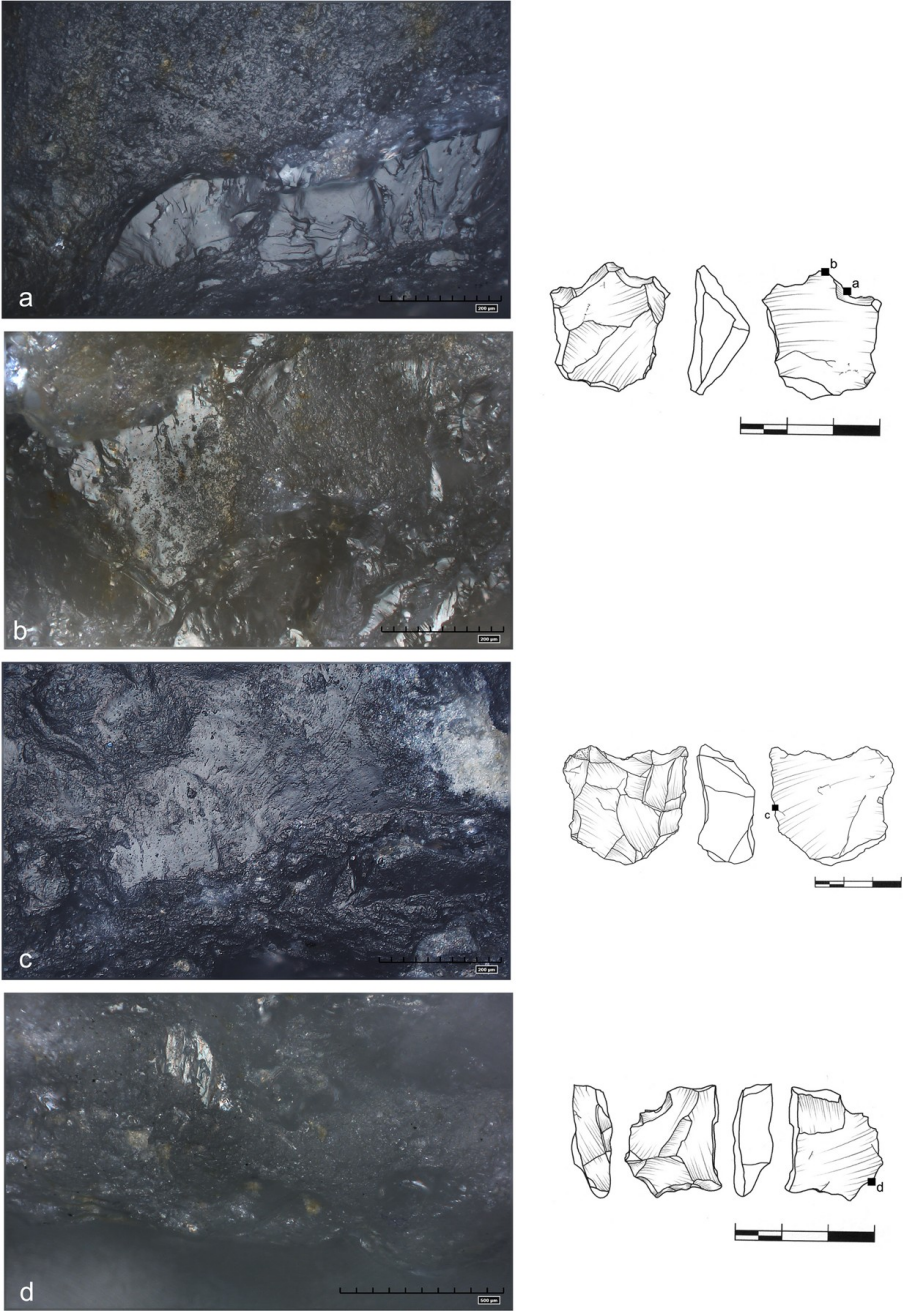
Examples of post depositional wear. Heavy abrasion of tool surfaces. Artefact no S29 (a,b) appeared dull even to the naked eye. Numerous irregular micro fractures, impact pits and furrows on art. no 340 (c), S8 (d); features include crushing and rounding of elevated surface parts.

Heavy post-depositional modification made the interpretation of function of half of the observed artefacts impossible. It can be distinguished from other types of wear by several features. It appeared in the form of scattered polish and chaotic abrasion of surface of tools. In extreme cases, almost the entire surface appeared dull (Fig 31A). Fresh scars visible on the heavily abraded surface are evidence of damage during or after excavation (Fig 31A). Numerous impact pits and irregular striations are distributed randomly and do not form any pattern. Impact fractures and cracks are found mostly on flat fracture or cleavage surfaces of the tools (Fig 31B).

## Discussion

Results of the EDAR (Eastern Desert Atbara River) area study indicate the presence of Acheulean occupation remains situated in the paleo-river system. EDAR 7 is mainly typified by fluvial sedimentary environment, characterised by two sedimentary facies. During pluvial periods channels bars (UNIT IA and IIA) and floodplains associated with grasslands and abandoned channels prevailed in the braided river (Unit IB and IIB). The advent of interpluvial periods interrupted the braided river system to activate aeolian and overland-flow process, typified by either relict stones as pavement, or silty sands possibly derived from wind-blown deposits under the arid- to semi-arid conditions.

Fluvial environmental context in the Eastern Sahara is basically characteristic of most of the Acheulean locations besides those from Saharan oases or artesian wells. Sai Island [5], Nag Ahmed el-Khalifa [29], Arkin 8 [28], Khor Abu Anga [30], al-Jamrab [31] and others [38, 91, 92] are Acheulean sites located either on the Nilotic terraces, its tributary banks or in smaller, dry watercourse systems. The buried channel system of EDAR area is not connected with any present-day active river valley and represents an ancient river course. Especially the fluvial system of the Atbara River considerably contributed to the northward dispersion of Acheulean tradition in the Middle Pleistocene. Acheulean sites from the region of Khashm el-Girba in the upper reaches of the river were already mentioned by Arkell [27]. Their stratigraphic context of Acheulean artefacts within the fluvial context was presented by Chmielewski [93] and later by Abbate [71]. Abbate’s research shows that a 50 m thick Pleistocene fluvial succession is extensively exposed in the area along the Atbara River from Khashm el-Girba to Halfa el-Jadida.

Outside Sahara, some of the main Eastern African Acheulean sites are similarly located in active fluvial environments, such as channel beds, with the occurrence of lithics accumulation [94]. One of such sites is Garba IV from Melka Kunture Formation along the upper Awash river in Ethiopia [95]. The sedimentary environment of EDAR 7 has certain similarity to that site. Morpho-sedimentary environments, however, differ from each other. The fluvial regime of EDAR 7 is predominantly controlled by paleoclimate change between semi-arid to arid and savanna climate, while that of Melka Kunture is mainly controlled by both paleoclimate and occasional volcanic activities in the highland area approximately above 2000 m (a.s.l.). In EDAR 7 sedimentary deposits are mostly composed of gravels, gravely sands and silty sands derived from the braided stream, while that of Melka Kunture is characterised by relatively fine sediments, i.e. silts and sandy silts in association with volcaniclastics or pyroclastics [95].

Unlike many Acheulean sites, which primarily contain bifaces with occasional debitage, the EDAR 7 assemblage consists of a full knapping sequence, from raw-material and initial cores, through debitage, to tools bearing evidence of usage. Consequently, EDAR 7 is interpreted as the remains of a buried Acheulean inventory, *Homo erectus* stone knapping leftovers. Furthermore, the assemblage contains not only hand-axes but also other large cutting tools. Our results highlight the co-occurrence of two reduction sequences in the same assemblage, one geared towards production of LCTs and the other based on the flaking of small debitage and production of flake tools, sometimes of microlithic proportions (endscrapers, perforators etc.). Amongst the LCTs, a considerable number of hand-axes and cleavers were produced from flake debitage. EDAR 7 is also one of the very few sites from Eastern Sahara where the Kombewa method was used in the production of LCTs. Assemblages produced with the use of giant flakes and the Kombewa method were previously only known in this region from a small Egyptian assemblage (site KAS-1) located near Bir Kiseiba in the Darb el Arba’in Desert, the area west of the Nile Valley between Dakhla Oasis in Egypt and Wadi Howar in central Sudan [96]. Outside the Eastern Saharan Africa, the Kombewa method has been documented from Eastern Africa [83, 97, 98], the Middle East [72] and lately also from the Arabian Peninsula [73, 99]. A striking feature of the EDAR 7 assemblage is the abundance of cleavers and “cleaver-like” hand-axes, most of which display features testifying to flake production. Asymmetrical and not well-shaped cleavers along with proto-hand-axes and hand-axes have also been lately reported from Gebel Karaiweb in the Red Sea Mountains (Sudan). According to Kobusiewicz and colleagues, this surface inventory could be attributed, although judging only by the stylistic and morphological features of the artefacts, to the turn of the Late Early and Middle Pleistocene [91].

The morphometric analysis showed similarities between the EDAR 7 assemblage and Site 14 in the Bir Sahara depression; the latter has a large number of hand-axes in cordiform and irregular amygdaloid forms. Morphological variation between Acheulean assemblages in NE Africa appears to be the result of adoption of production methods to the locally available raw material.

Use wear analysis revealed numerous post-depositional alterations on the surfaces of observed artefacts (Fig 30). Analogous micro-traces and edge deformations were discovered on much younger (9500–5700 cal. BP) tools from Sai Island in Northern Sudan [59]. Experiments show that such patterns can be generated by water transport. Abrasion extent seems to depend on artefact stabilisation. Without it artefact roll downstream freely and abrasion, in extreme cases heavy and readily visible, could cover its entire surface. Stabilisation, e.g. by sand or pebbles, limits the changes only to the exposed parts of the artefacts [100]. Aeolian abrasion, on the other hand, creates numerous impact pits, flat fractures and cracks [60]. Both cases were apparent on the EDAR 7 sample, which made the analysis more challenging.

Use wear traces were observed on 7 out of 15 of the analysed tools (Table 6). Most artefacts combined more than one type of movement but were probably only used for one material, either wood or bone. Similar use wear was replicated during numerous experiments *e*.*g*. by I. Clemente Conte and J.F. Gibaja Bao [52], or by Knutsson’s team [58, 101] and has analogies in archaeological record. Several artefacts with wood use wear were found at the Oldowan sites of Konjera South (cutting and scraping) [53] and Koobi Fora in Kenya [102]. Early use of wooden tools is further supported by residue analyses, which revealed remains of phytoliths, for example on Acheulean stone tools from Paninj in Tanzania [103].

On three artefacts, the traces were less developed but could still be interpreted as resulting from cutting of undetermined soft material (Fig 28A, 28C and 28D). Similar forms are sometimes connected with butchering activities [90, 104].

In the Eastern Saharan Africa the Acheulean appears to be stylistically diversified, judging by individual excavated sites all belonging to the late phases of the industry (~0,6–0,3 Ma), and despite a paucity of data [17, 105]. Among those late assemblages, there are: sets of asymmetrical hand-axes from Egyptian oases at site E-72-1 [11]; a wide range of ovates (20% of the assemblage) and chopping tools from the Nubian Arkin 8 site in the area of Wadi Halfa [28], and the EDAR 7 assemblage described above, consisting of numerous cleavers and hand-axes prepared on flakes. The archaeological sequence at site 8-B-11 on Sai Island, which contains a Late Acheulean assemblage with fresh, large lanceolate hand-axes interstratified with horizons containing Sangoan hand-axes from the Middle Stone Age [5], makes this picture even more complex.

Wendorf, Close and Schild [106] pointed out that enormous geomorphic changes have taken place since the Acheulean. Writing in 1987 they stated that that no Acheulean living floors had ever been found and that the only geologically *in situ* artefacts recovered had come from the lower portions of almost completely deflated spring vents; none had ever been discovered “associated with ancient river deposits”. By *in situ*, they surely meant the BS-14 site (trench 3), where a small assemblage of 237 artefacts embedded in spring sands below a calcium carbonate cap was excavated. These artefacts, which were made of brownish quarzitic sandstone, were not eroded but fresh and semi-fresh [12, 107]. From 1987, with the exception of the Sai Island site commented below, not much had changed in the Eastern Saharan African Acheulean, until the discovery of the EDAR sites, where Acheulean assemblages have been found buried within a paleo-fluvial context.

Few Acheulean assemblages from NE Africa have been reliably dated. Besides EDAR 7 and EDAR 135 in the Eastern Desert, there is only one Sudanese site, 8-B-11 on Sai Island in the Nile, where a layer of aeolian sand separates the Acheulean bed from the lowest Middle Stone Age deposits of Sangoan affinity [86, 108, 109]. OSL dating of this aeolian sand yielded an age of 223 ± 19 ka [5], providing a minimum age for the Acheulean on Sai Island. The Egyptian oases have yielded much more information regarding the chronology of the Acheulean [107, 110]. In the Bir Sahara–Bir Tarfawi depression, episodes of lake expansion and regression suggest that episodically habitable landscapes existed in the region from approximately 400 ka (possibly as early as MIS 11) until the end of the Acheulean and into the MSA (up to MIS 3) [111–113]. An assemblage from Bir Sahara, BS-14 (E99C14), partly located in a primary context (trench 3), yielded an OSL age of 210 ± 18 ka which is considered to be too young (Romuald Schild, pers. com.). The Acheulean from Bir Sahara, although currently undated, is presumed to be older than 300 ka [112, 114]. Several small Acheulean assemblages or singular bifaces were found in a stratigraphic contexts near Bir Safsaf, Bir Kiseiba and Wadi Arid [96, 106]. Luminescence dating of the sediment just below the Acheulean horizon from site E-85-2 at Dag Dag Safsaf, Bir Safsaf, yielded ages of 308 ± 28 ka (GdTL-644) and 268 ± 26 ka (GdTL-645) [114].

The published ages for the oldest Acheulean from Eastern Sahara indicate human occupations during MIS 9, or possibly during MIS 11 as suggested by the depositional contexts of the Acheulean artefacts. The OSL dating of EDAR confirms both these assumptions. The OSL age from Unit IB (280 ± 27 ka), possibly indicates deposition of the underlying Acheulean levels during a warmer episode in MIS9 [115]. However, a sample from the same stratigraphic context, just above the top of the UNIT IA, from the site EDAR 135 (western wall) located ~ 100 m from EDAR 7, gave an OSL age of 391 ± 30 ka, indicating that the Acheulean artefacts from UNIT IA could be older, i.e. MIS 11, MIS13 (500–400 ka) or earlier. This possibility is supported by the morphology of the artefacts, suggesting that they have been reworked into younger deposits, and the abundance of cleavers and the use of the Kombewa method in EDAR 7. Such developed Acheulean technologies of large flake production and high values of standardised made-on-large-flake cleavers and hand-axes first appear in Africa around 1.0 Ma continuing up to the Middle Pleistocene and are labelled as ‘Large Flake Acheulean’ stage [116–119]. This is not a characteristic feature of the other Acheulean Nubian sites dated to the ~300–200 ka period, including some other Acheulean sites from EDAR–e.g. EDAR 133 [38] and EDAR 135 (lower cultural horizon) [32], therefore a wide possible chronology for EDAR 7 inventory could fall into the ‘Large Flake Acheulean’ phase. This phase could possibly be also represented by the Acheulean inventory from another EDAR neighbouring site—EDAR 6, where made-on-large-flake cleavers are common (S21 Fig) [120]. Corresponding assumptions were proposed by Haynes and colleagues [96], based on the technological features of a few Acheulean artefacts from KAS-1, whose subsurface inventory–however small–appears to be similar to EDAR 7 (presence of cleavers and Kombewa technology). This chronology would suggest that the stone tools found at EDAR 7 are older than those found at BS-14 [12], BT-A [121], KAS-1 [96], Site 047 [71], and KO10 [122]), making it the oldest Acheulean assemblage from the Eastern Saharan Africa dated to the early Middle Pleistocene or even to the turn of the Late Early and Middle Pleistocene. Chronological analogies to the EDAR area among Acheulean sites from the Arabian Peninsula are worth mentioning here as they demonstrate Acheulean presence there during MIS 7 [123, 124], which is chronologically similar to the Acheulean horizon from EDAR 135 [32] but younger than site EDAR 7 discussed here.

## Conclusions

This paper analyses Acheulean site EDAR 7 registered lately in the Sudanese Eastern Desert, where geoarchaeological methods identified this archaeological assemblage. Summing up, EDAR 7 has yielded a rare Eastern Saharan Acheulean assemblage, which has been dated and compared to other Acheulean materials from the region. The site has yielded new information on several aspects of Saharan Acheulean, including its chronology, environmental setting as well as behavioural and technological specificity of the lithic inventory. The chronology established for EDAR 7 points out that this is so far the oldest recognised Acheulean assemblage from the Eastern Sahara indicating its age as MIS 11–13 or earlier.

The EDAR 7 archaeological horizon is associated with the environment of the ephemeral braided stream and floodplain (UNIT IA). The buried channel system of the EDAR area is not connected with any present-day active river valley and represents an ancient river course not studied before. The advents of aridity during the interpluvial periods frequently caused drying up of the stream channels, bars or floodplains. It makes sense to presume that *Homo erectus* hominins occasionally occupied and exploited the gravel-beds of the dried stream or overbank areas of the stream, possibly for the purpose of procuring gravel stones. It is common to find Acheulean stone artefacts, made mainly from cobble-stones derived from the fluvial process contiguous to EDAR 7 in particular.

The archaeological record in the EDAR area, which is under constant threat from gold mining, is of considerable importance for understanding the earliest prehistory of the Eastern Sahara. Its scientific potential, highlighting the role of ancient Saharan watercourses has only been outlined. Further research in this region has the potential to shed more light on early migration routes out of Africa.

## Supporting information

S1 File EDAR 7 lithics database. (ACCDB)

S1 Fig Example of landmarks’ location on the hand-axe outline. The red dot marks the fixed landmark located on the tip and the black dots marks equally located semi-landmarks.
(TIF)

S2 Fig Results of procrustes superimposition for each site. a (EDAR 7), b (EDAR 133), c (Kharga Oasis 10), d (Dakhla Oasis, site E-72-1), e (Bir Sahara 14).
(TIF)

S3 Fig Preheat plateau tests and dose recovery for sample EDAR7-3. Recovered dose values show good agreement with the given dose (within ±10%: red dotted line) for preheat temperatures between 180 and 260˚C. A preheat temperature of 220˚C and a cut-heat of 160˚C were selected for equivalent dose determinations on the EDAR7 site, and preheats of 260°C followed by a 220°C cut-heat was used on the sample EDAR-135-S6.
(TIF)

S4 Fig Dose response curve and signal from an aliquot of the sample EDAR7-3. The OSL characteristics of the quartz from Sudan show a rapidly decaying signal and continuously growing dose response curve, which makes it well suited for application of the SAR protocol used in this study.
(TIF)

S5 Fig Radial plots of equivalent doses for single aliquots of quartz from samples EDAR7-1, EDAR7-2, EDAR7-3, EDAR7-4, EDAR7-5 and EDAR-135-S6. The grey bars are centred around the calculated CAM dose. Data points within the 2 standard errors of CAM are black filled. N is the number of accepted aliquots, and OD the overdispersion of the sample.
(TIF)

S6 Fig Quartzite cores from EDAR 7. Unpatterned, multiple platform cores: a (art. no. 295), b (art. no. 308); discoidal core: c (art.no. S 53).
(TIF)

S7 Fig Microlithic quartzite cores from EDAR 7. a: discoidal (art. no. 75), b: unidirectional (art. no. 248).
(TIF)

S8 Fig Selection of cores from EDAR 7. a: quartzite unpatterned, multiple platform core (art. no. 295), b: quartzite unpatterned, multiple platform core (art. no. 308), c: quartzite unidirectional core (art. no. 146).
(TIF)

S9 Fig Biface attributes. Number of scars, directions of negatives, natural surfaces and retouch. The drawing shows eight locations of negatives directions.
(TIF)

S10 Fig Methods of LCT production. 1 –cleaver made on cobble; 2 –hand-axe made on cobble; 3 –cleaver made on flake; 4 –hand-axe made on flake; 5 –hand-axe made on Kombewa flake.
(TIF)

S11 Fig Experiment carried out to check the possibility of detaching large flakes from giant core and producing LCT’s from local raw materials. a (G.M. using direct percussion with hard hammer in production of large flakes), b (M.E. using direct percussion with hard hammer in production of large flakes), c (large flakes produced during experiment), d (cleaver made form quartzite large flake), e (hand-axe made from fine-grained rhyolite), f (hand-axe made from coarse-grained rhyolite).
(TIF)

S12 Fig Denticulate. Quartzite; a (art. no. 356), b (art. no. 447), c (art. no. 462). X signs denote recent damage.
(TIF)

S13 Fig Notches. Rhyolite (a, b) and quartzite (c, d); a (art. no. 49), b (art. no. 104), c (art. no. 106), d (art. no. 340).
(TIF)

S14 Fig Notches. Quartzite; a (art. no. 48), b (art. no. 266), c (art. no. 337), d (art. no. 452), e (art. no. 339), f (art. no. 73), g (art. no. 265), h (art. no. 69).
(TIF)

S15 Fig Perforators. Quartzite (a–e, g) and rhyolite (f); a (art. no. 472), b (art. no. 85), c (art. no. 142), d (art. no. S9), e (art. no. S29), f (art. no. 65), g (art. no. S52).
(TIF)

S16 Fig Perforators. Quartzite; a (art. no. 46), b (art. no. 381), c (art. no. S8), d (art. no. 357), e (art. no. 292), f (art. no. 549).
(TIF)

S17 Fig Endscrapers and sidescrapers. Quartzite (a–f) and rhyolite (g). Endscrapers: a (art. no. 260), b (art. no. 42), c (art. no. 538) and sidescrapers: d (art. no. 375), e (art. no. 40), f (art. no. 26), g (art. no. 247).
(TIF)

S18 Fig Sidescrapers. Quartzite; a (art. no. 134), b (art. no. 536), c (art. no. 451), d (art. no. S51).
(TIF)

S19 Fig Composite tools and retouched flakes. Quartzite (a–g) and rhyolite (h). Composite tools: a (perforator/sidescraper, art. no. 327), b (denticulate/sidescraper, art. no. 59), c (perforator/sidescraper, art. no. 421), d (denticulate/sidescraper, art. no. 523) and retouched flakes: e (art. no. 473), f (art. no. 25), g (art. no. 226), h (art. no. 89).
(TIF)

S20 Fig Retouched flakes. Quartzite (a, c-e) and rhyolite (b). a (art. no. 25), b (art. no. 125), c (art. no. 162), d (art. no. 489), e (art. no. 150).
(TIF)

S21 Fig EDAR 6. Cleavers made on large flakes. a: rhyolite cleaver on Kombewa flake from the surface of the site; b: trench I/2017—rhyolite cleaver on Kombewa flake; c: trench I/2017—rhyolite cleaver made on flake.
(TIF)

S1 Table The single-aliquot regenerative-dose procedure used in this study. (DOCX)

S2 Table Frequencies of state of preservation of EDAR 7 inventory (chips and debris excluded). (DOCX)

S3 Table Core types from EDAR 7 with their frequencies according to raw materials. (DOCX)

S4 Table Exploitation stage of cores. (DOCX)

S5 Table Dimensions (mm) and weight (g) of complete cores (n = 68). (DOCX)

S6 Table Dimensions (mm) and weight (g) of complete flakes (n = 197). (DOCX)

S7 Table Dorsal face blow directions of flakes. (DOCX)

S8 Table Flake butt types. (DOCX)

S9 Table Large flakes from EDAR 7 (mm and g). (DOCX)

S10 Table Blank type and preservation state of bifaces. (DOCX)

S11 Table Dimensions of LCT (mm). (DOCX)

S12 Table Retouched tools. Dimensions of complete flake tools (n = 84) (mm and g).
(DOCX)

S13 Table Edge modification and delineation type, location of retouch; retouched edge length (complete tools only). (DOCX)

S14 Table Percentage value of variance of selected main principal components. (DOCX)

S15 Table Results of the MANOVA and PERMANOVA tests. (DOCX)

S16 Table Results of similarity in pairs implemented in the PERMANOVA test. KO–Kharga Oasis, site nr 10; BS- Bir Sahara site nr 14, DO–Dakhla Oasis site nr E-72-1.
(DOCX)
