# Supplementary figures and images for: The oldest Homo erectus buried lithic horizon from the Eastern Saharan Africa. EDAR 7 - an Acheulean assemblage with Kombewa method from the Eastern Desert, Sudan
Source: PLoS One. 2021 Mar 23;16(3):e0248279. doi: 10.1371/journal.pone.0248279 (PMC7989774; doi:10.1371/journal.pone.0248279)

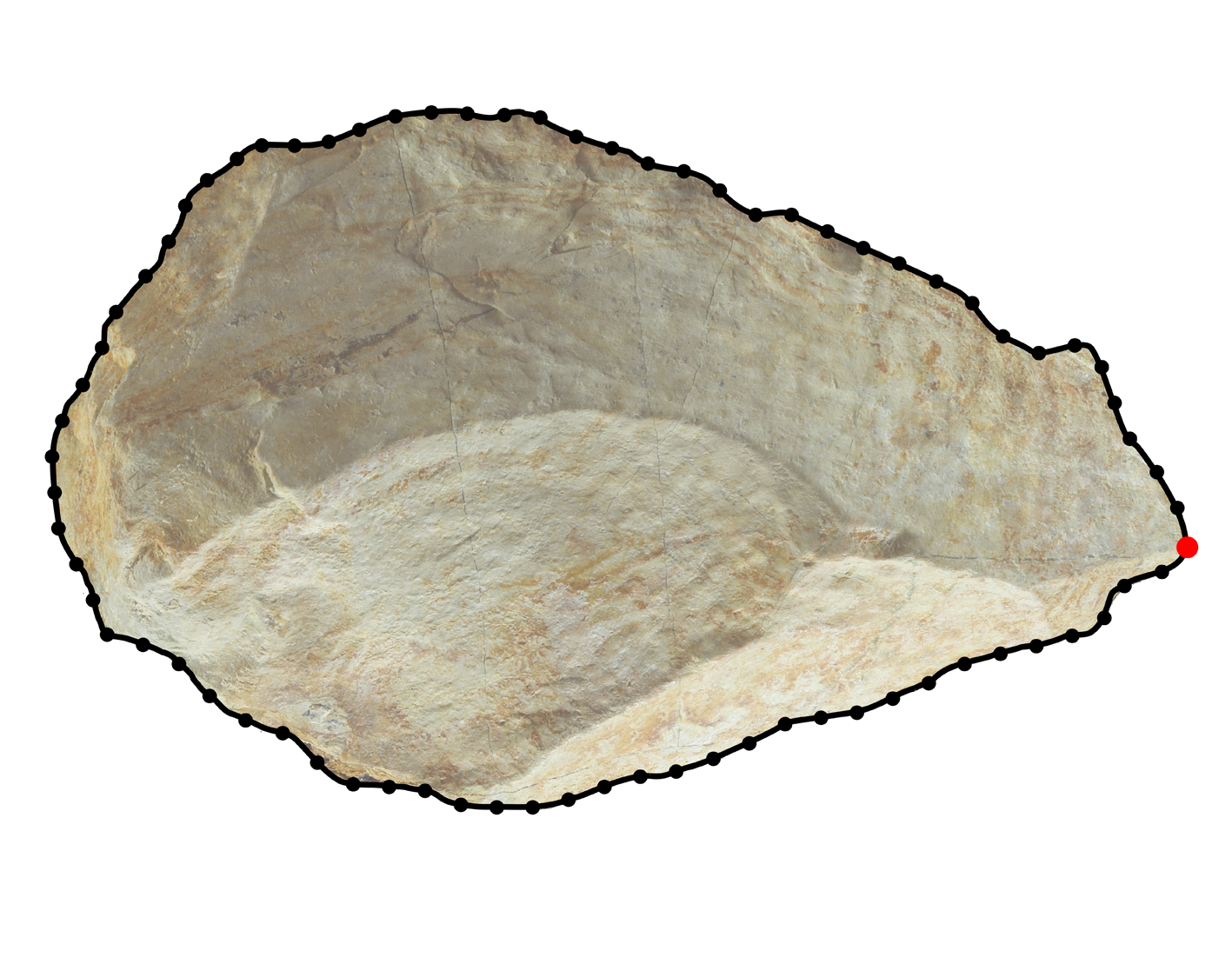

Supplement: S1 Fig — The red dot marks the fixed landmark located on the tip and the black dots marks equally located semi-landmarks. (TIF) [file pone.0248279.s002.tif]

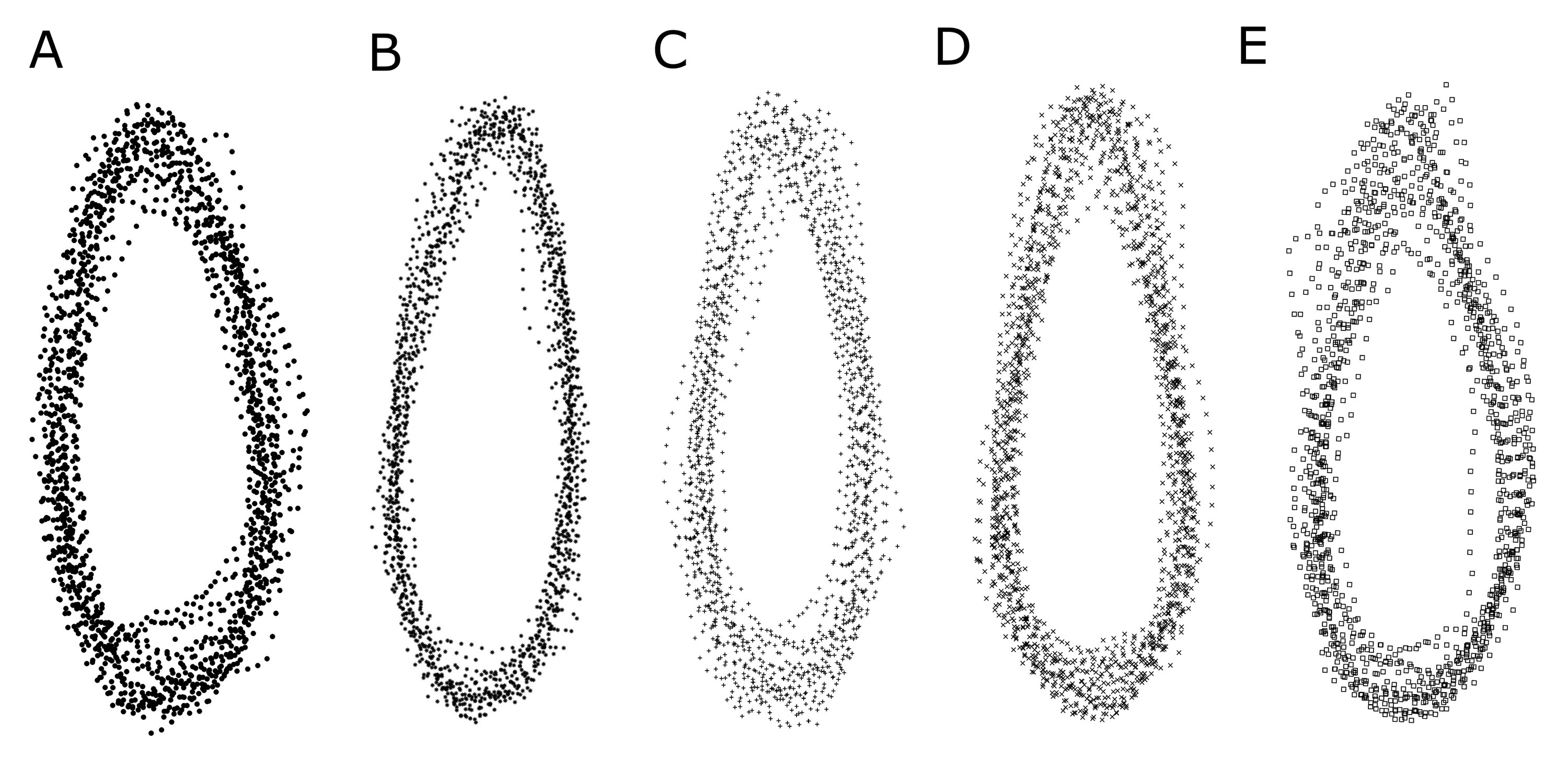

Supplement: S2 Fig — a (EDAR 7), b (EDAR 133), c (Kharga Oasis 10), d (Dakhla Oasis, site E-72-1), e (Bir Sahara 14). (TIF) [file pone.0248279.s003.tif]

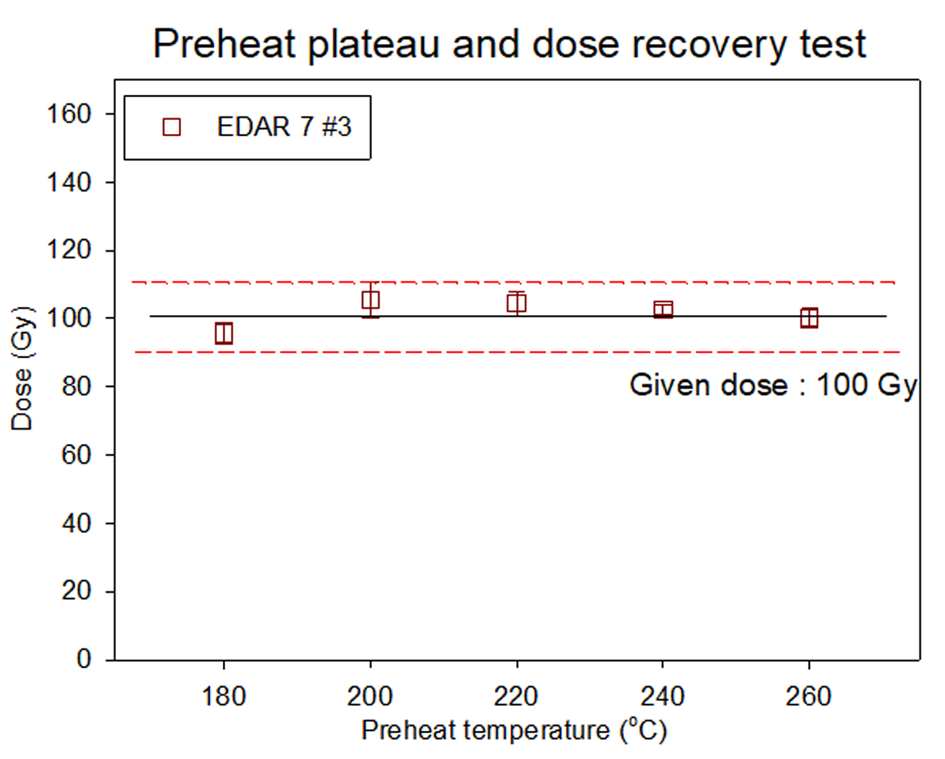

Supplement: S3 Fig — Recovered dose values show good agreement with the given dose (within ±10%: red dotted line) for preheat temperatures between 180 and 260˚C. A preheat temperature of 220˚C and a cut-heat of 160˚C were selected for equivalent dose determinations on the EDAR7 site, and preheats of 260°C followed by a 220°C cut-heat was used on the sample EDAR-135-S6. (TIF) [file pone.0248279.s004.tif]

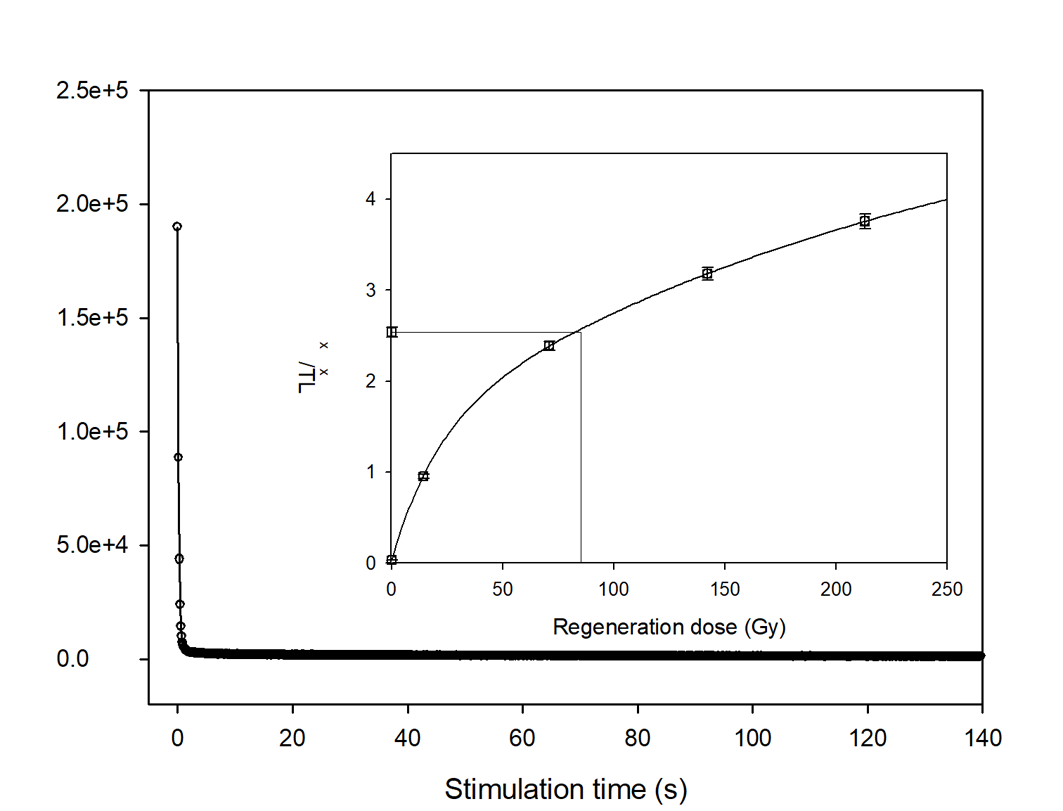

Supplement: S4 Fig — The OSL characteristics of the quartz from Sudan show a rapidly decaying signal and continuously growing dose response curve, which makes it well suited for application of the SAR protocol used in this study. (TIF) [file pone.0248279.s005.tif]

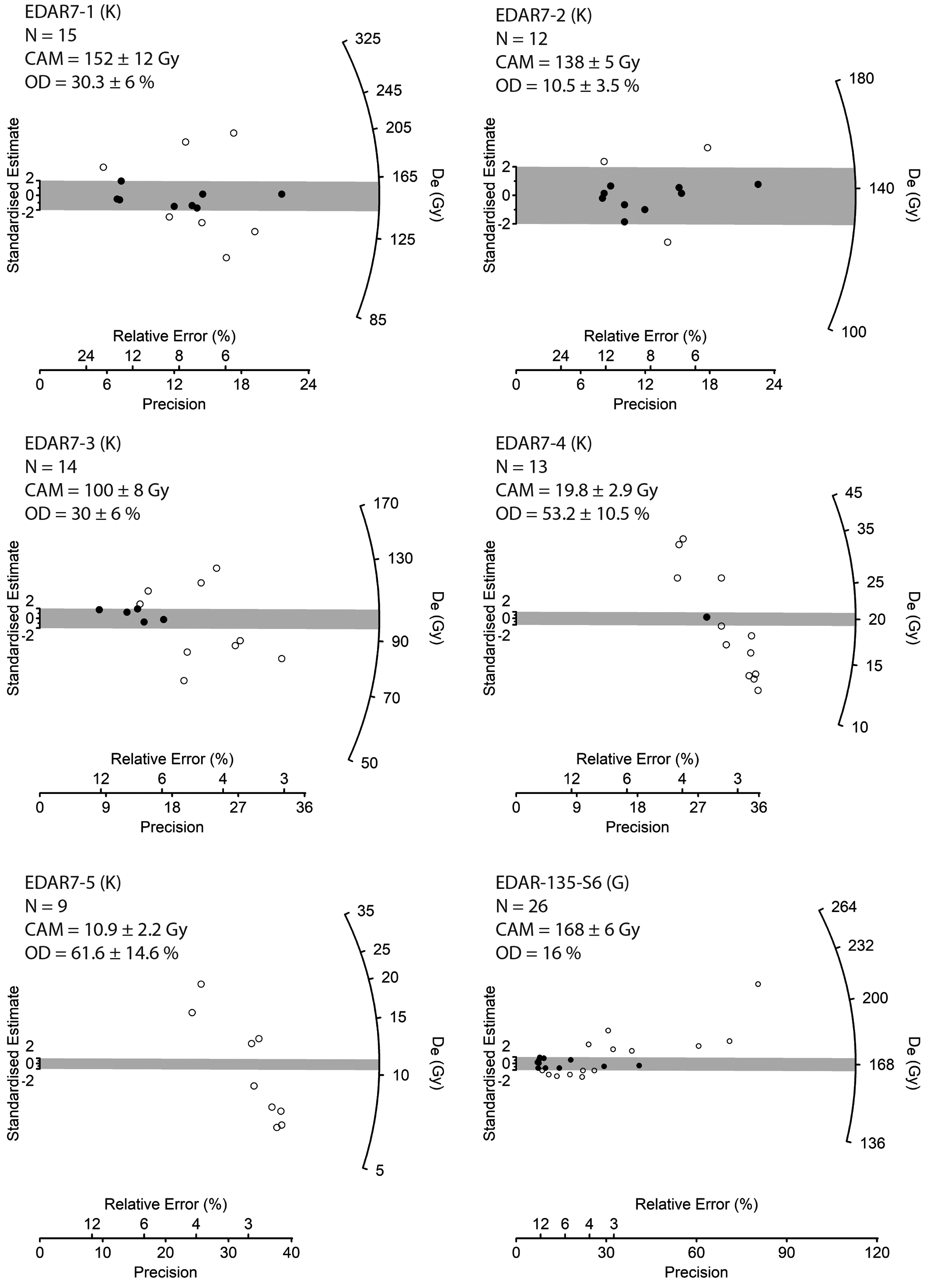

Supplement: S5 Fig — Data points within the 2 standard errors of CAM are black filled. N is the number of accepted aliquots, and OD the overdispersion of the sample. (TIF) [file pone.0248279.s006.tif]

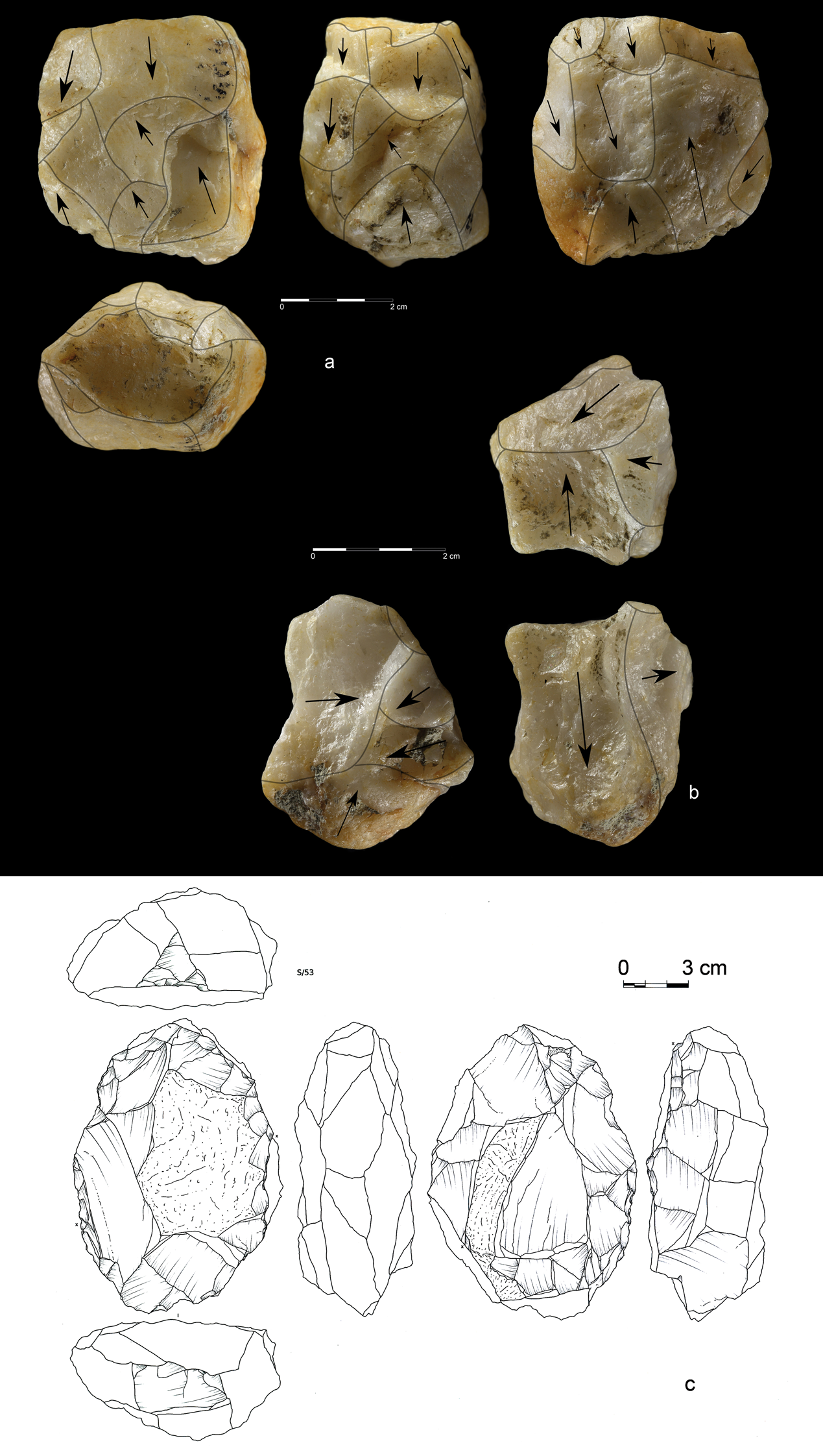

Supplement: S6 Fig — Unpatterned, multiple platform cores: a (art. no. 295), b (art. no. 308); discoidal core: c (art.no. S 53). (TIF) [file pone.0248279.s007.tif]

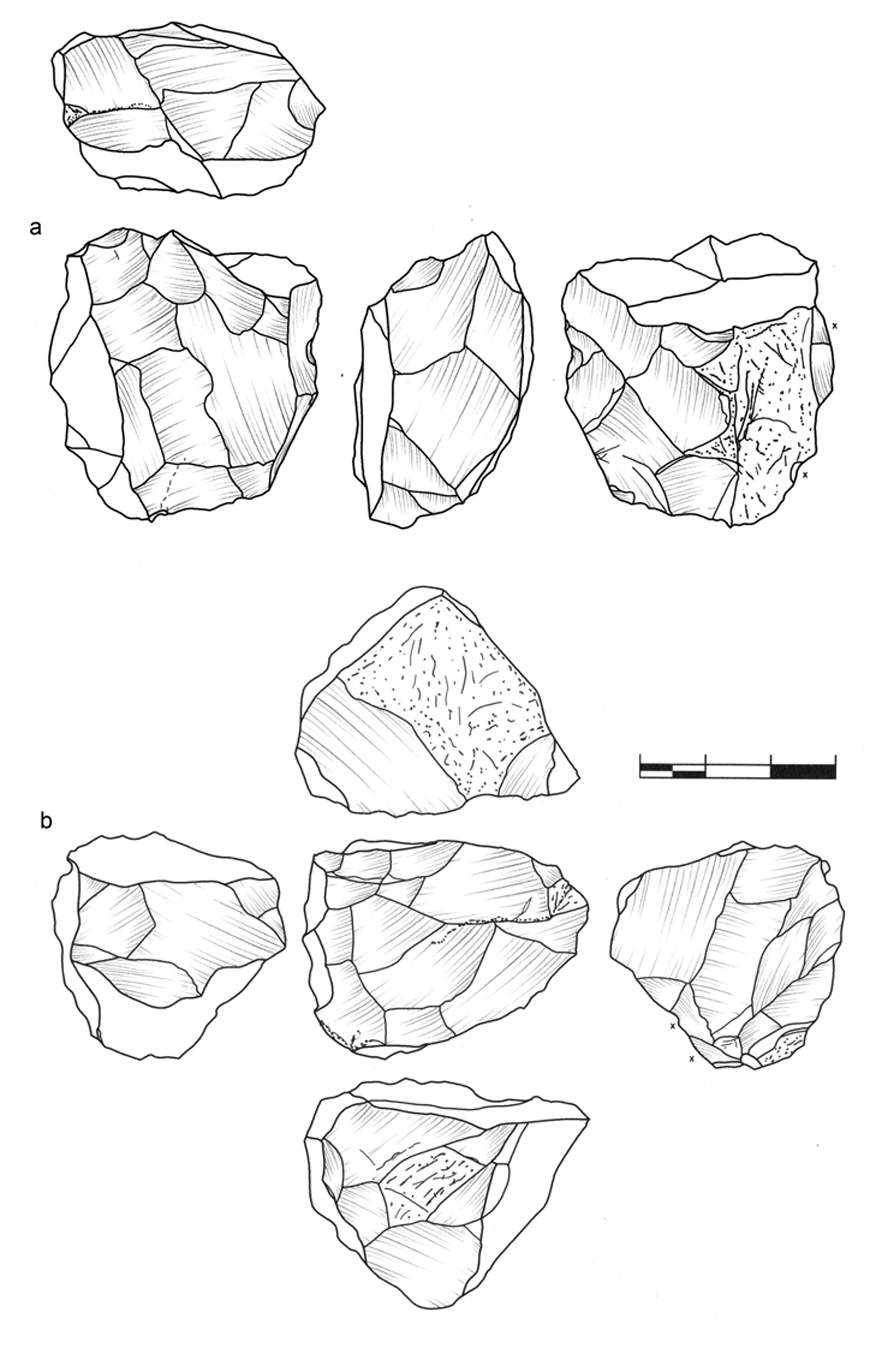

Supplement: S7 Fig — a: discoidal (art. no. 75), b: unidirectional (art. no. 248). (TIF) [file pone.0248279.s008.tif]

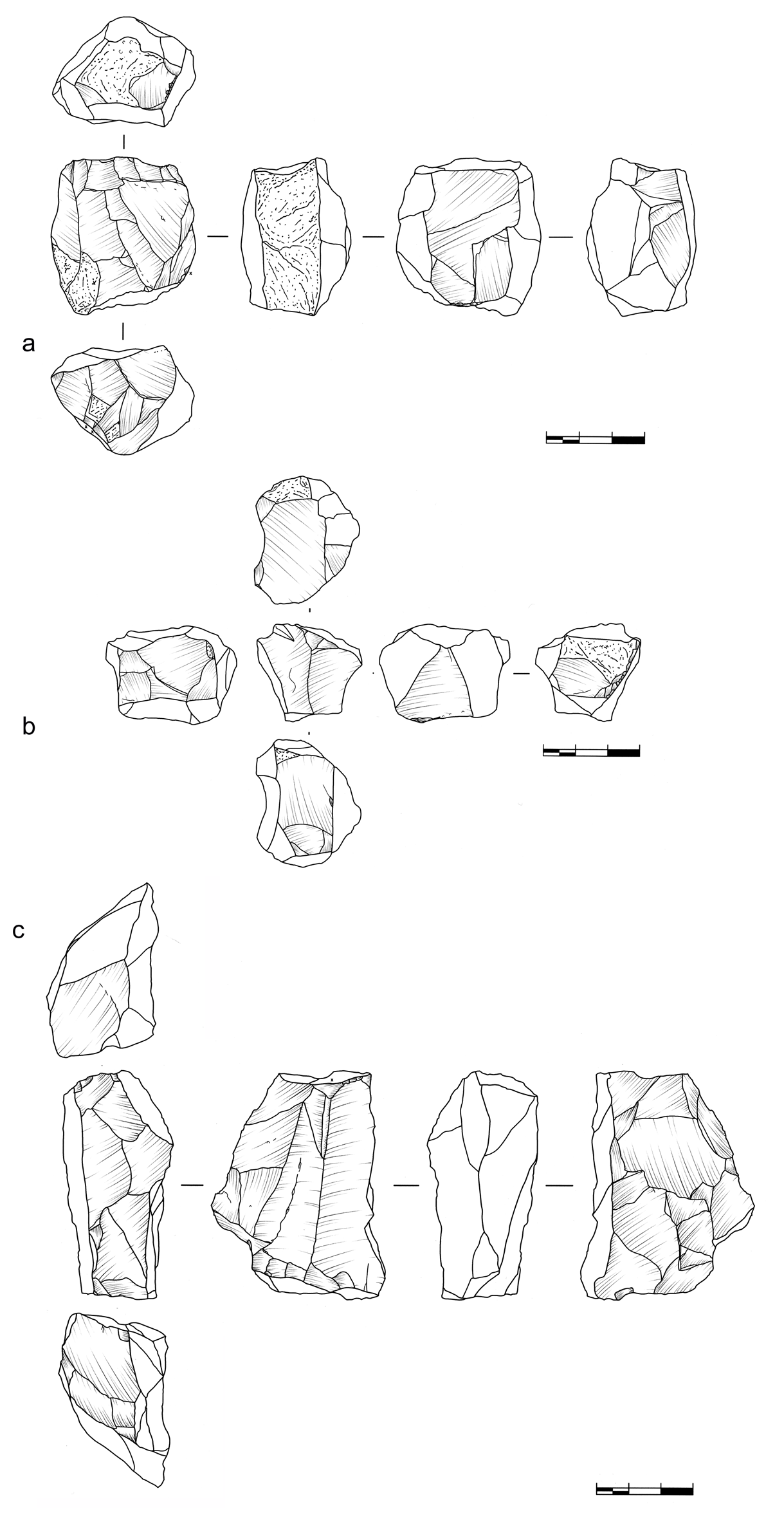

Supplement: S8 Fig — a: quartzite unpatterned, multiple platform core (art. no. 295), b: quartzite unpatterned, multiple platform core (art. no. 308), c: quartzite unidirectional core (art. no. 146). (TIF) [file pone.0248279.s009.tif]

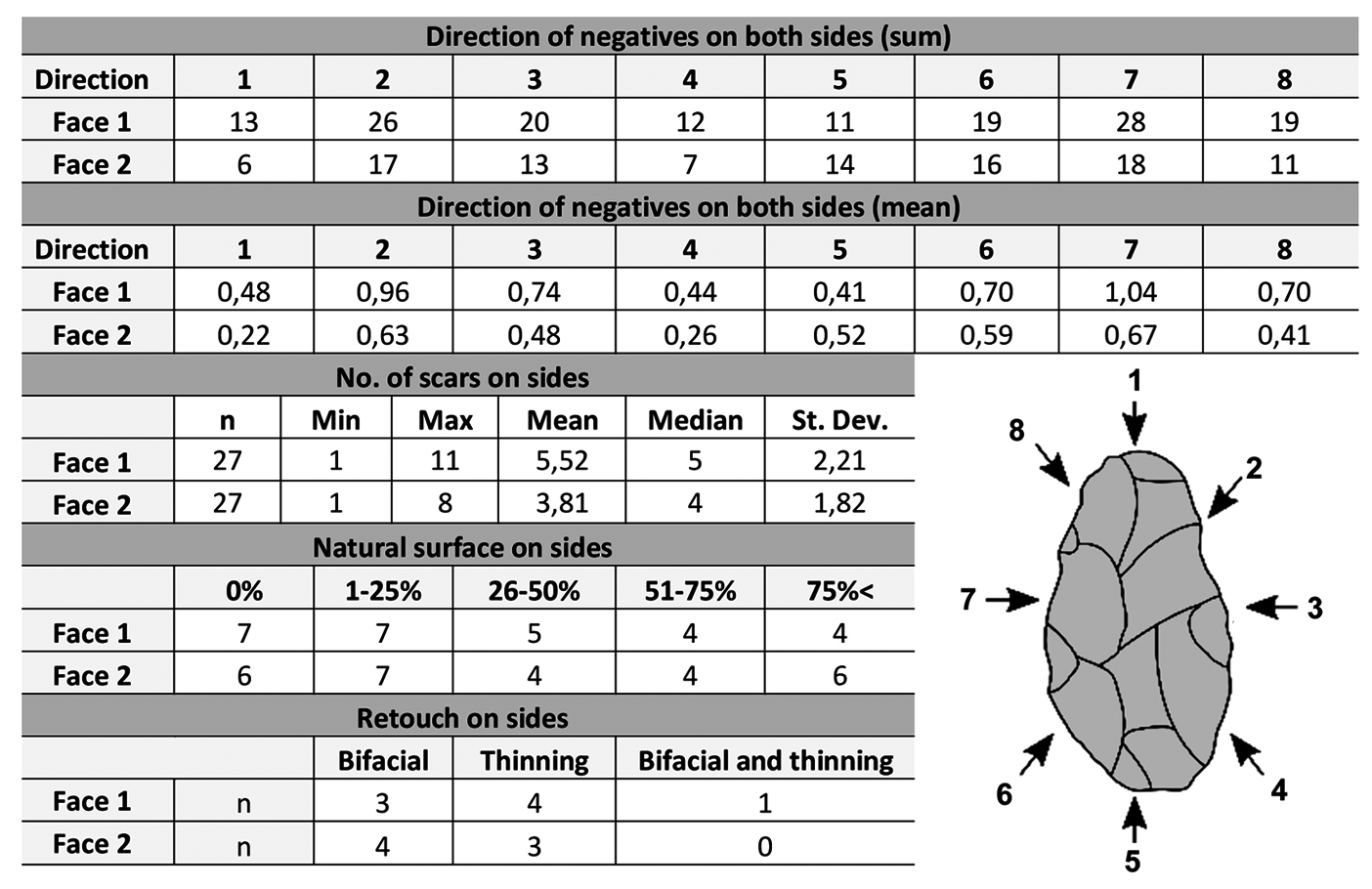

Supplement: S9 Fig — Number of scars, directions of negatives, natural surfaces and retouch. The drawing shows eight locations of negatives directions. (TIF) [file pone.0248279.s010.tif]

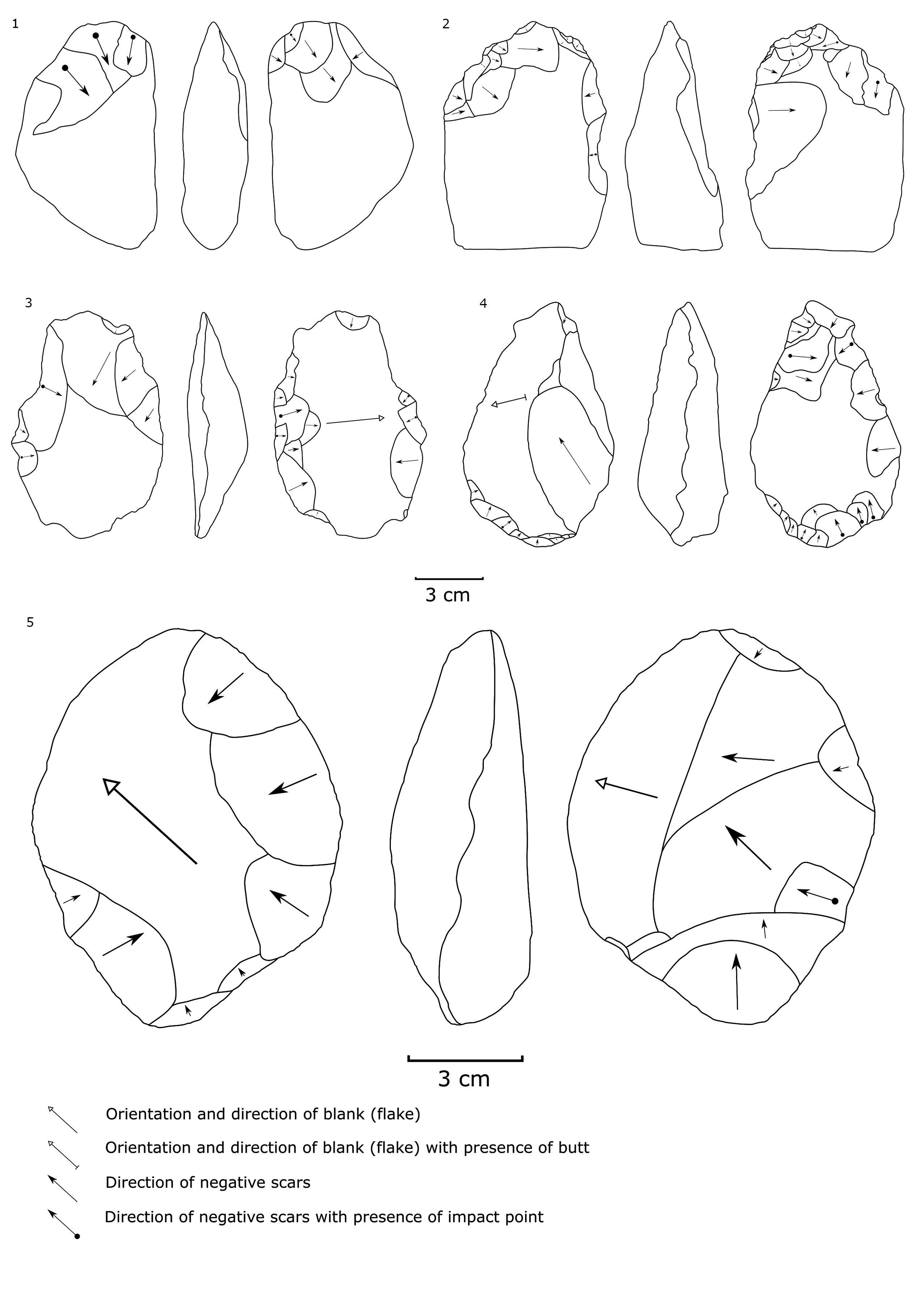

Supplement: S10 Fig — 1 –cleaver made on cobble; 2 –hand-axe made on cobble; 3 –cleaver made on flake; 4 –hand-axe made on flake; 5 –hand-axe made on Kombewa flake. (TIF) [file pone.0248279.s011.tif]

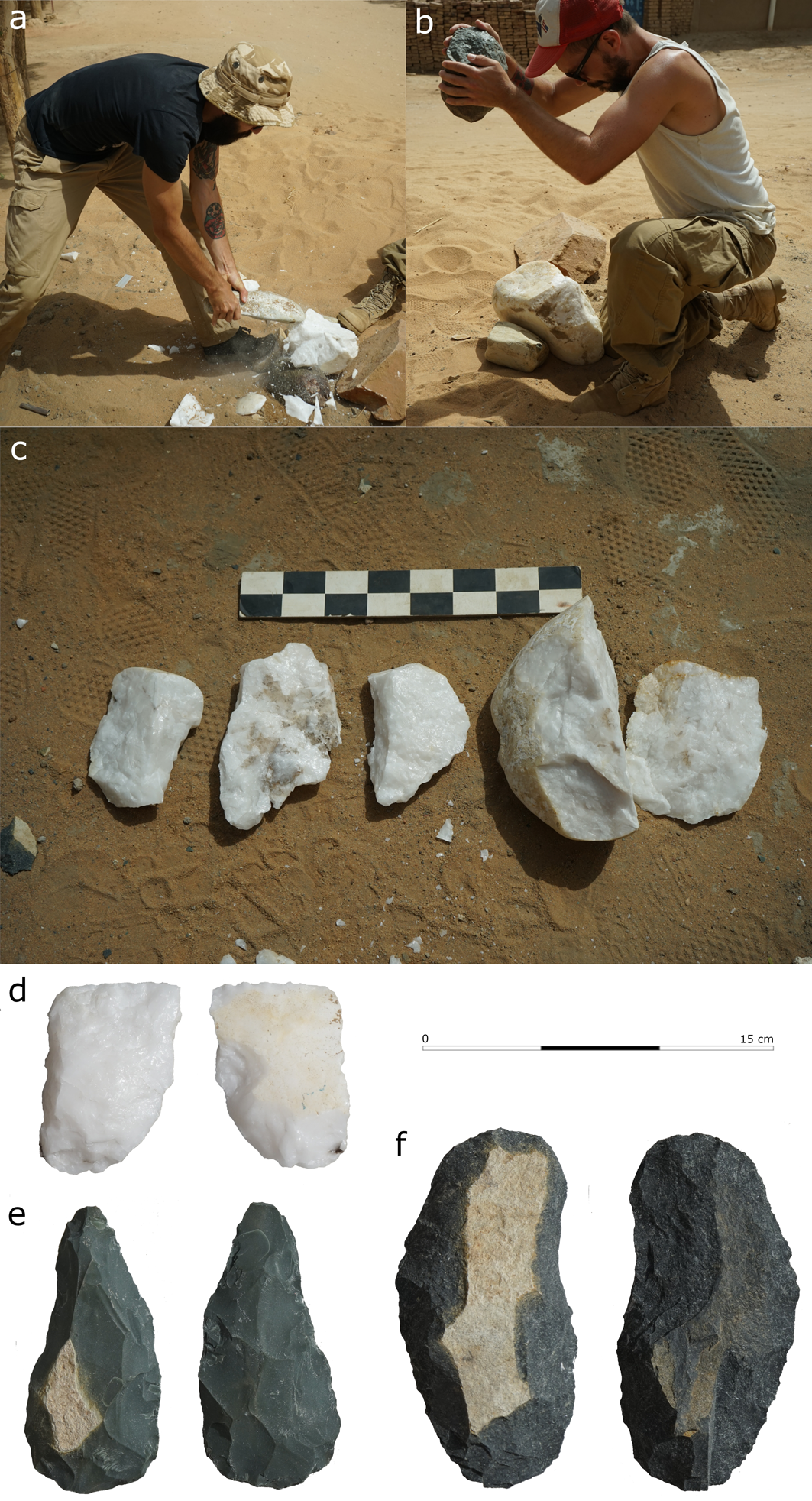

Supplement: S11 Fig — a (G.M. using direct percussion with hard hammer in production of large flakes), b (M.E. using direct percussion with hard hammer in production of large flakes), c (large flakes produced during experiment), d (cleaver made form quartzite large flake), e (hand-axe made from fine-grained rhyolite), f (hand-axe made from coarse-grained rhyolite). (TIF) [file pone.0248279.s012.tif]

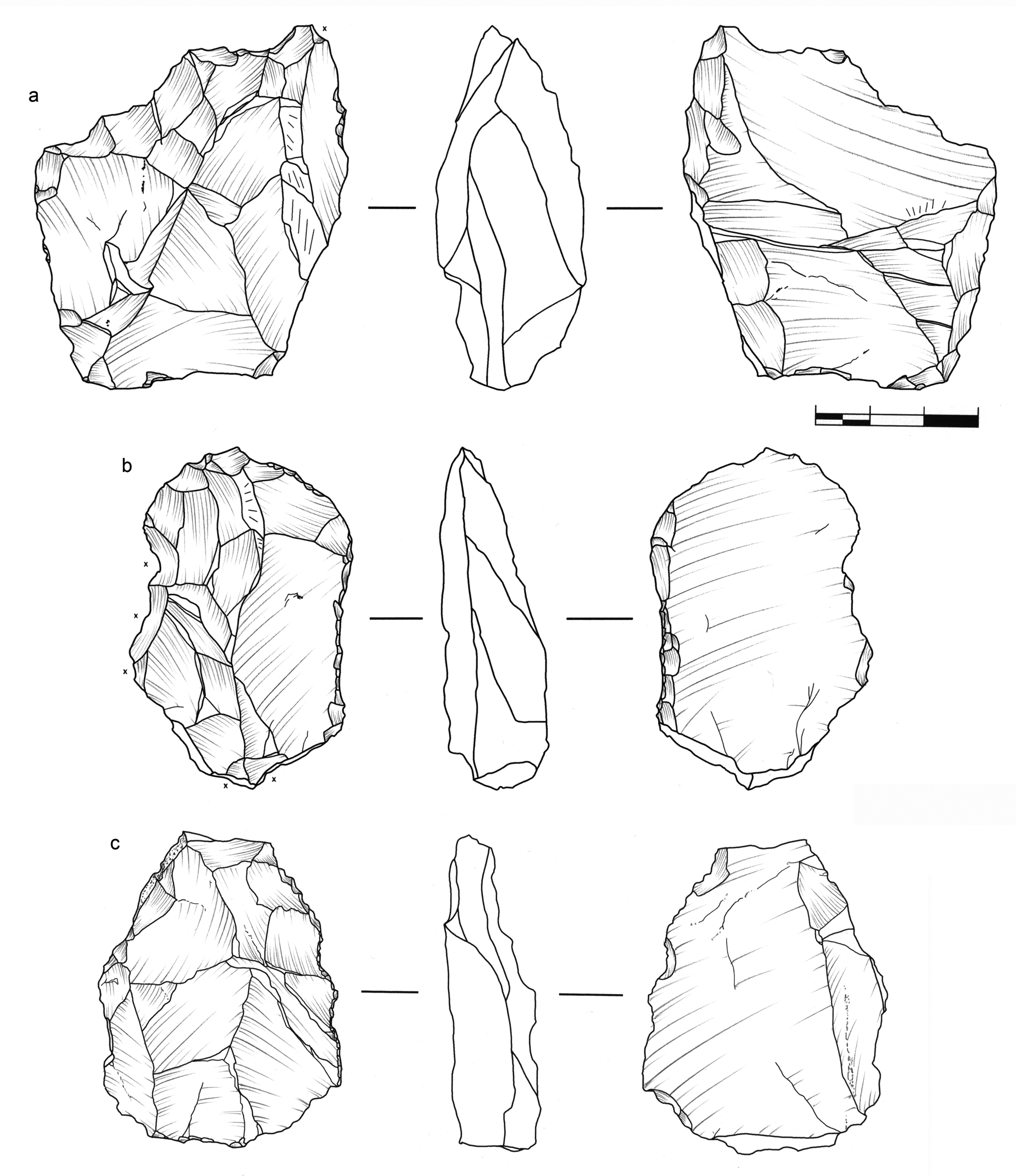

Supplement: S12 Fig — Quartzite; a (art. no. 356), b (art. no. 447), c (art. no. 462). X signs denote recent damage. (TIF) [file pone.0248279.s013.tif]

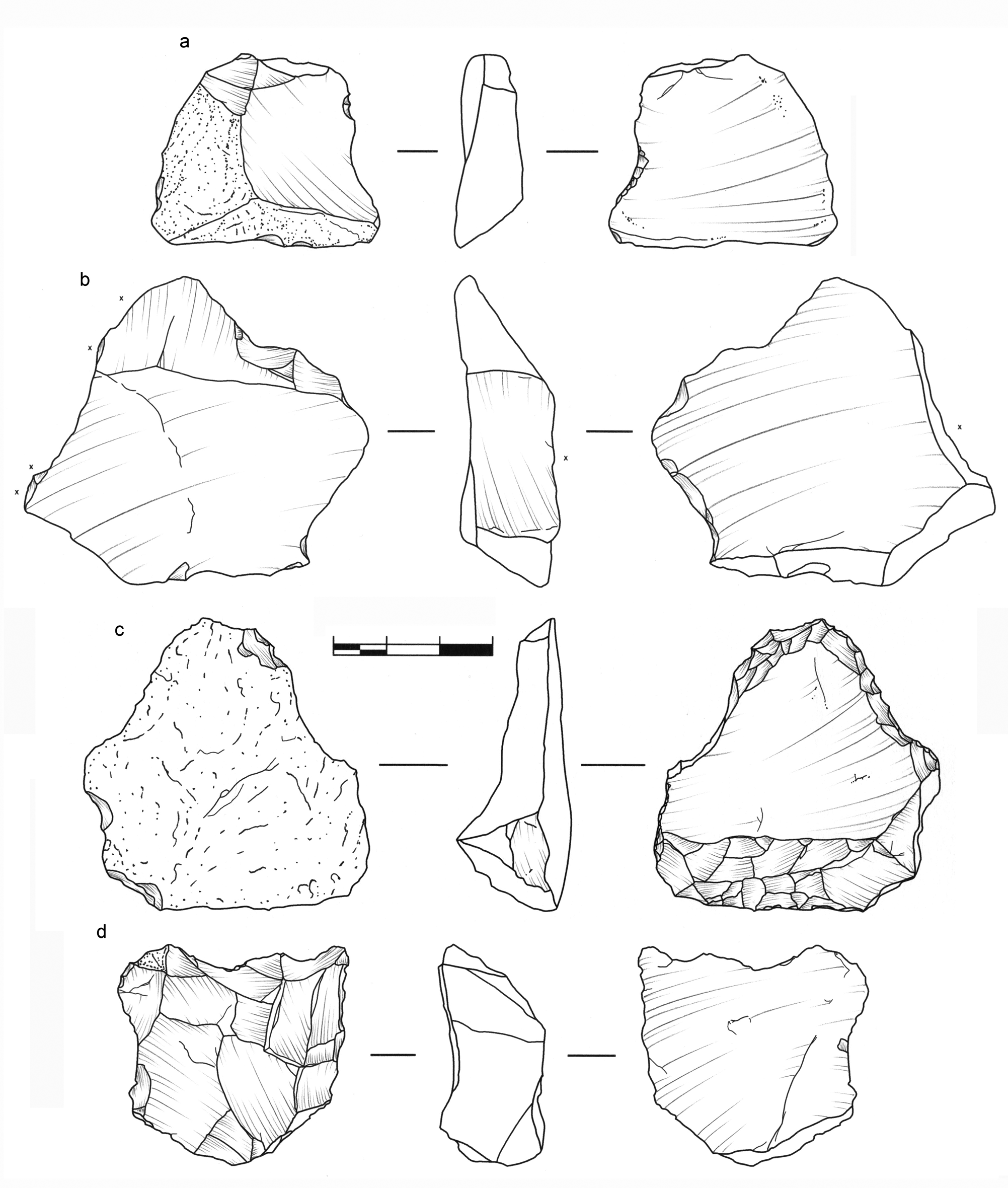

Supplement: S13 Fig — Rhyolite (a, b) and quartzite (c, d); a (art. no. 49), b (art. no. 104), c (art. no. 106), d (art. no. 340). (TIF) [file pone.0248279.s014.tif]

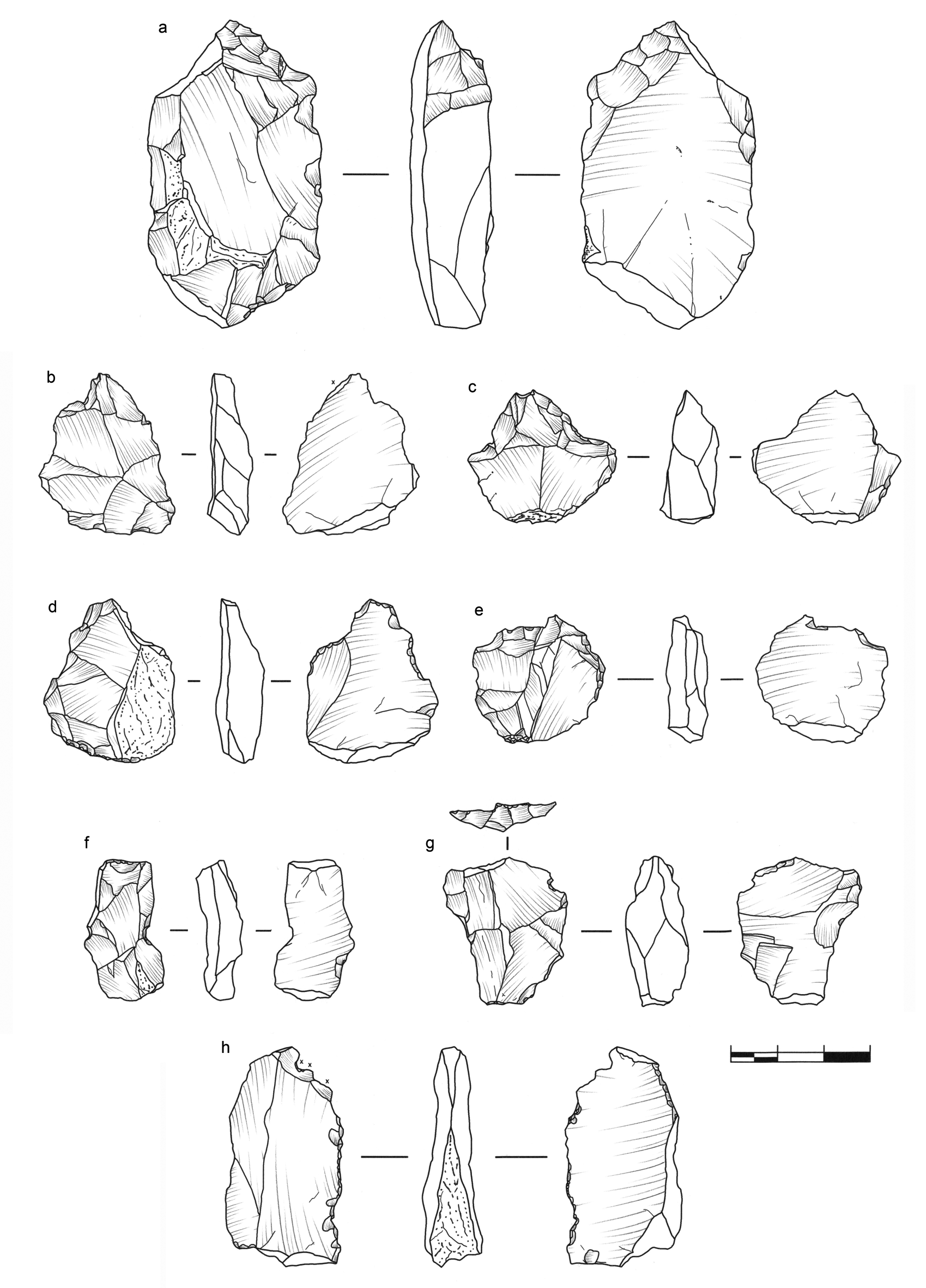

Supplement: S14 Fig — Quartzite; a (art. no. 48), b (art. no. 266), c (art. no. 337), d (art. no. 452), e (art. no. 339), f (art. no. 73), g (art. no. 265), h (art. no. 69). (TIF) [file pone.0248279.s015.tif]

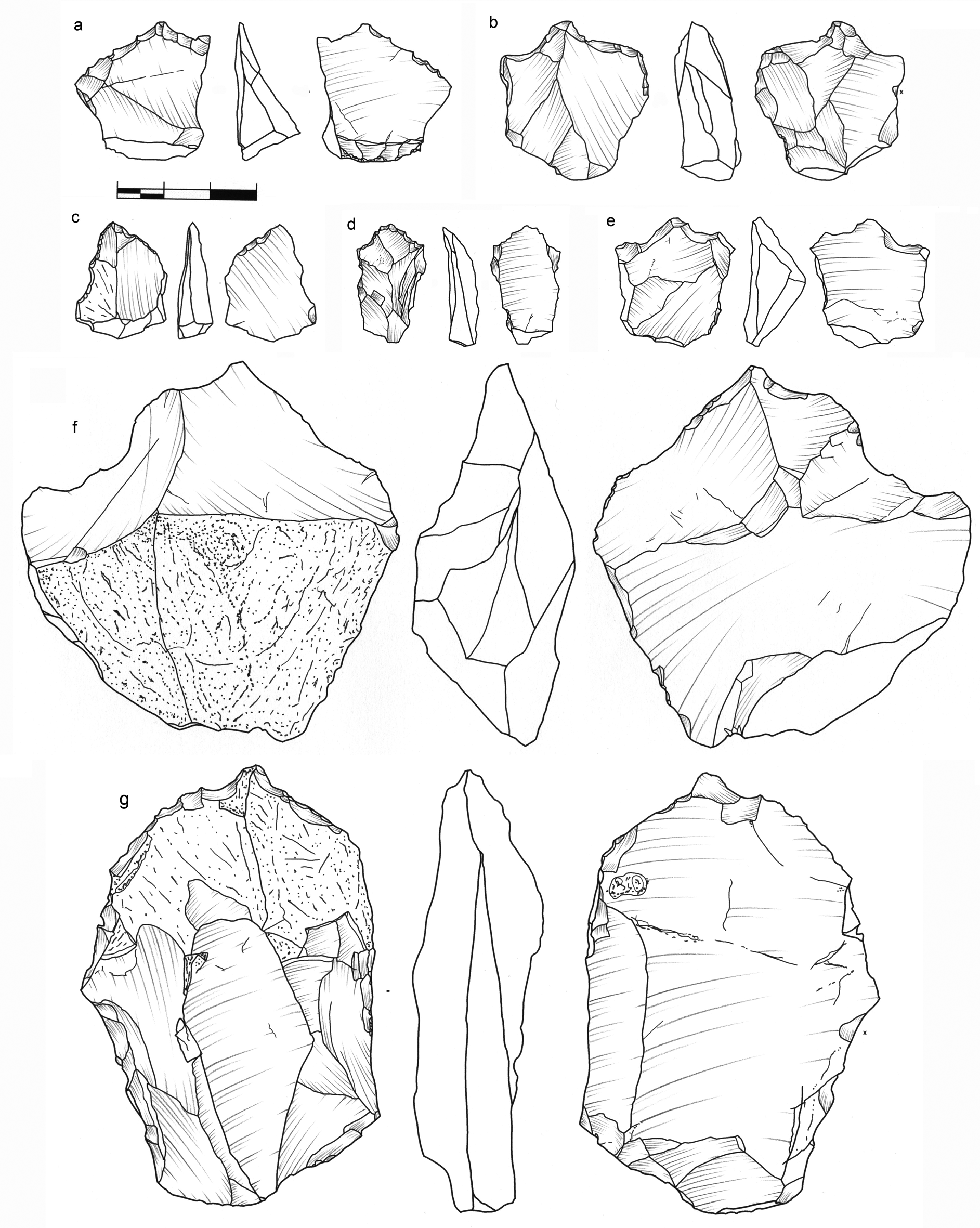

Supplement: S15 Fig — Quartzite (a–e, g) and rhyolite (f); a (art. no. 472), b (art. no. 85), c (art. no. 142), d (art. no. S9), e (art. no. S29), f (art. no. 65), g (art. no. S52). (TIF) [file pone.0248279.s016.tif]

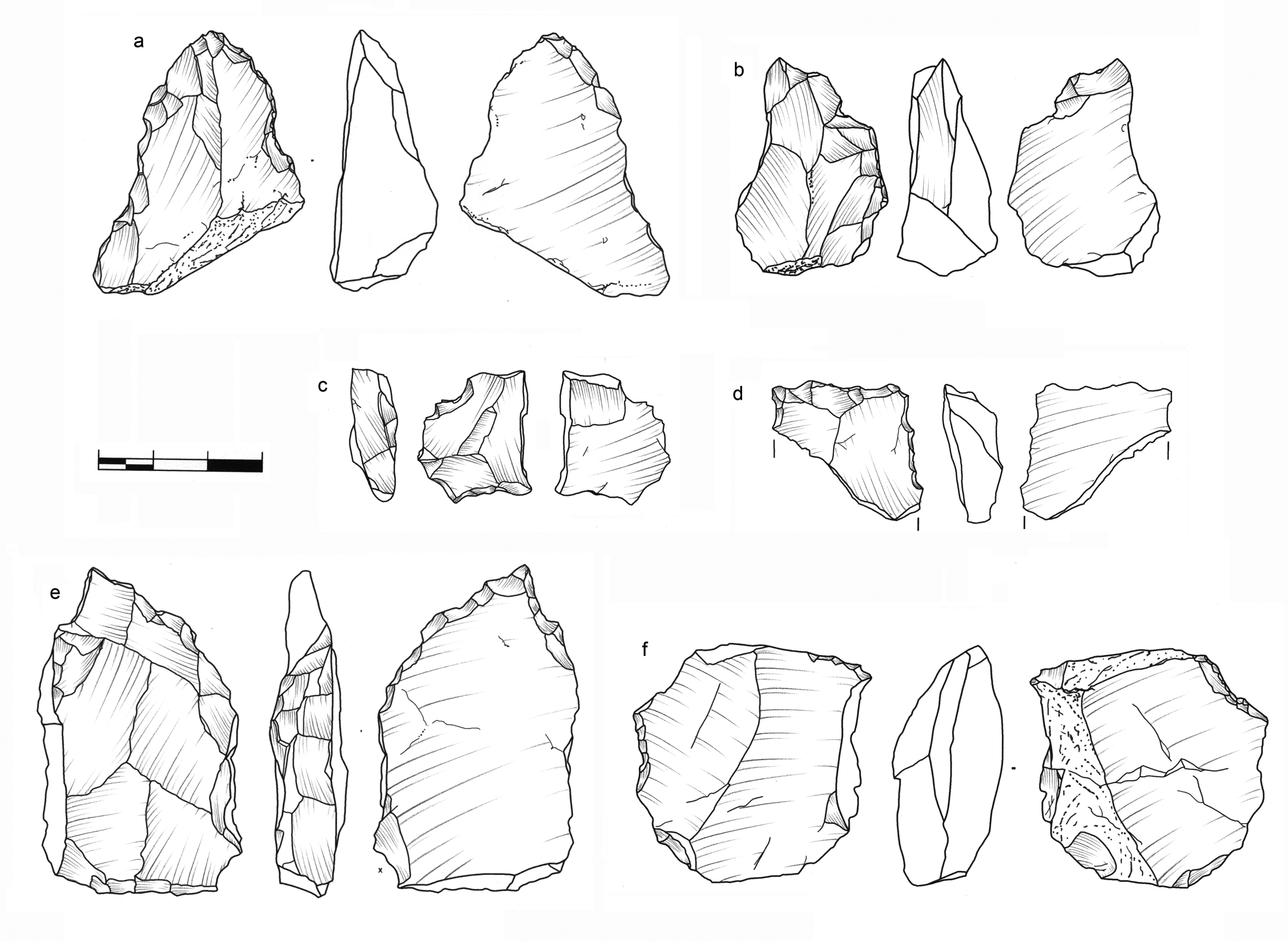

Supplement: S16 Fig — Quartzite; a (art. no. 46), b (art. no. 381), c (art. no. S8), d (art. no. 357), e (art. no. 292), f (art. no. 549). (TIF) [file pone.0248279.s017.tif]

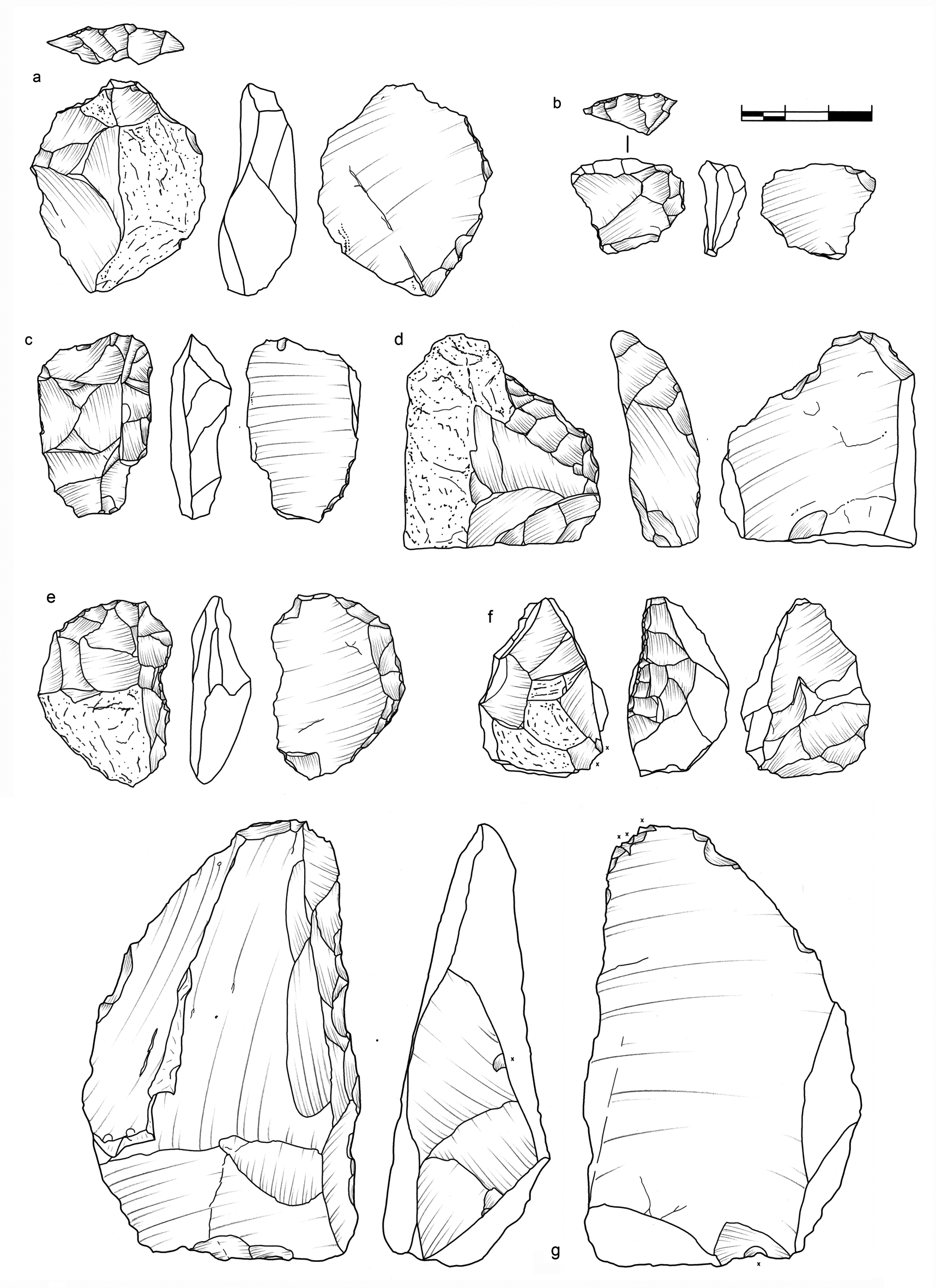

Supplement: S17 Fig — Quartzite (a–f) and rhyolite (g). Endscrapers: a (art. no. 260), b (art. no. 42), c (art. no. 538) and sidescrapers: d (art. no. 375), e (art. no. 40), f (art. no. 26), g (art. no. 247). (TIF) [file pone.0248279.s018.tif]

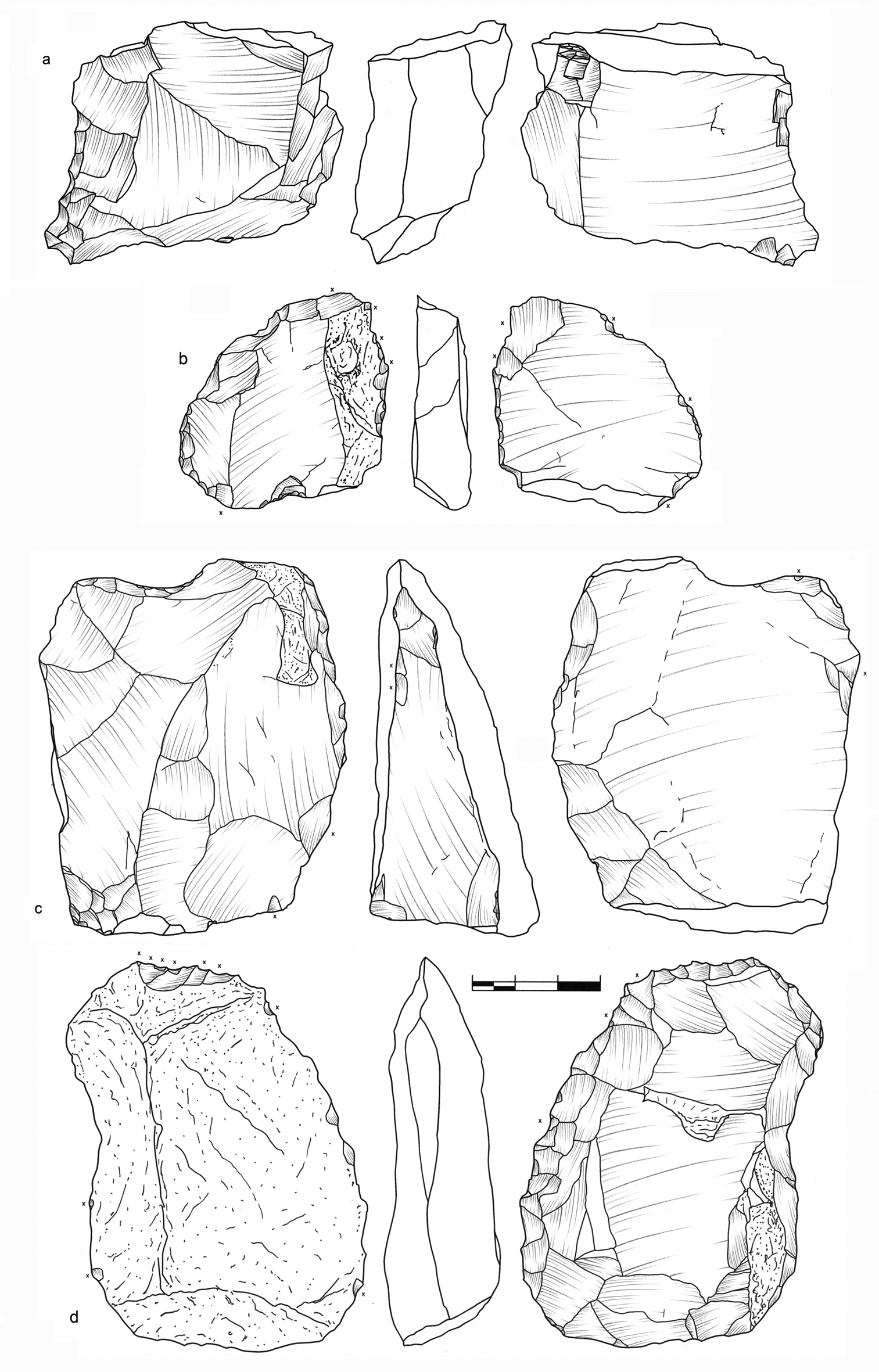

Supplement: S18 Fig — Quartzite; a (art. no. 134), b (art. no. 536), c (art. no. 451), d (art. no. S51). (TIF) [file pone.0248279.s019.tif]

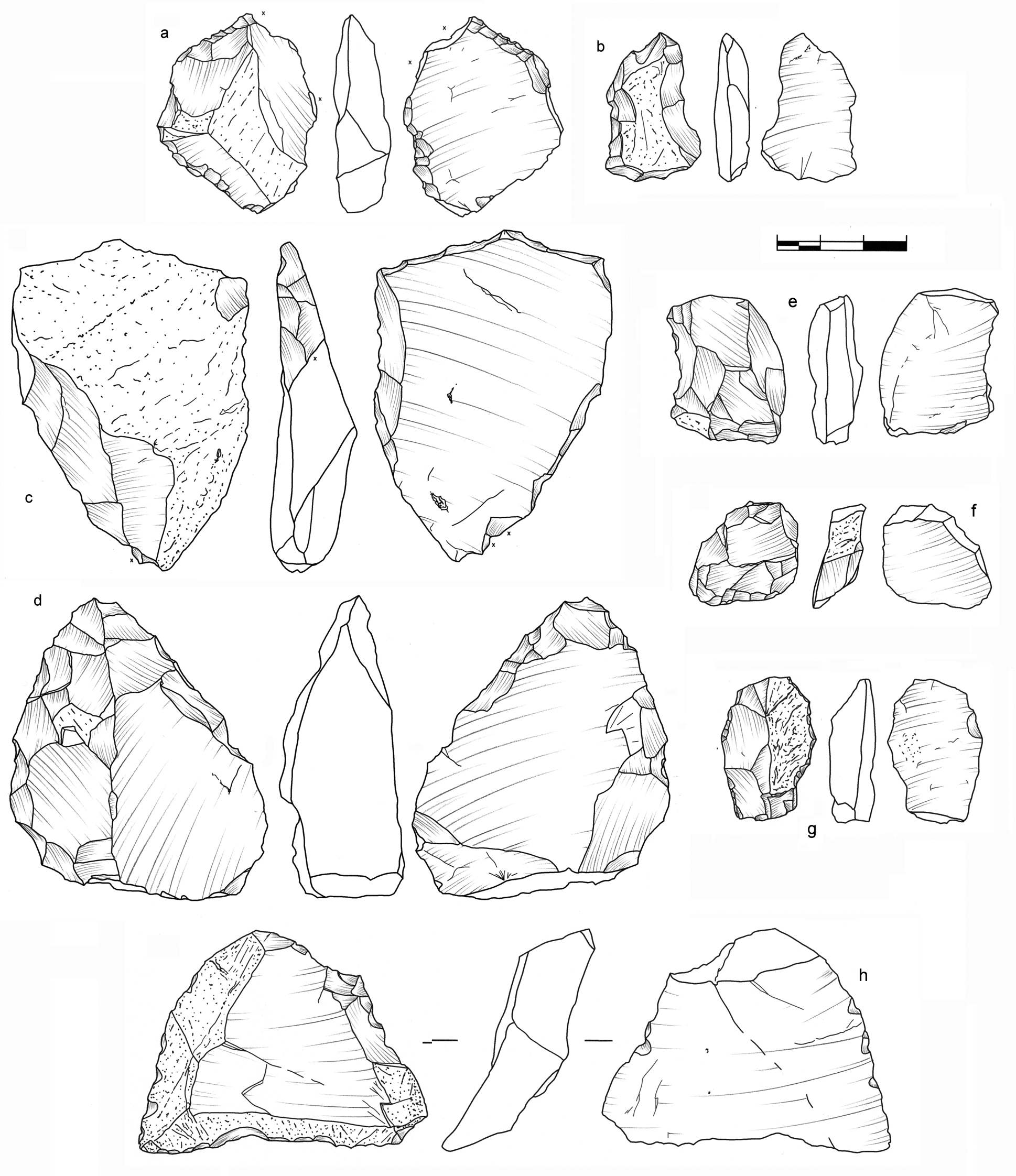

Supplement: S19 Fig — Quartzite (a–g) and rhyolite (h). Composite tools: a (perforator/sidescraper, art. no. 327), b (denticulate/sidescraper, art. no. 59), c (perforator/sidescraper, art. no. 421), d (denticulate/sidescraper, art. no. 523) and retouched flakes: e (art. no. 473), f (art. no. 25), g (art. no. 226), h (art. no. 89). (TIF) [file pone.0248279.s020.tif]

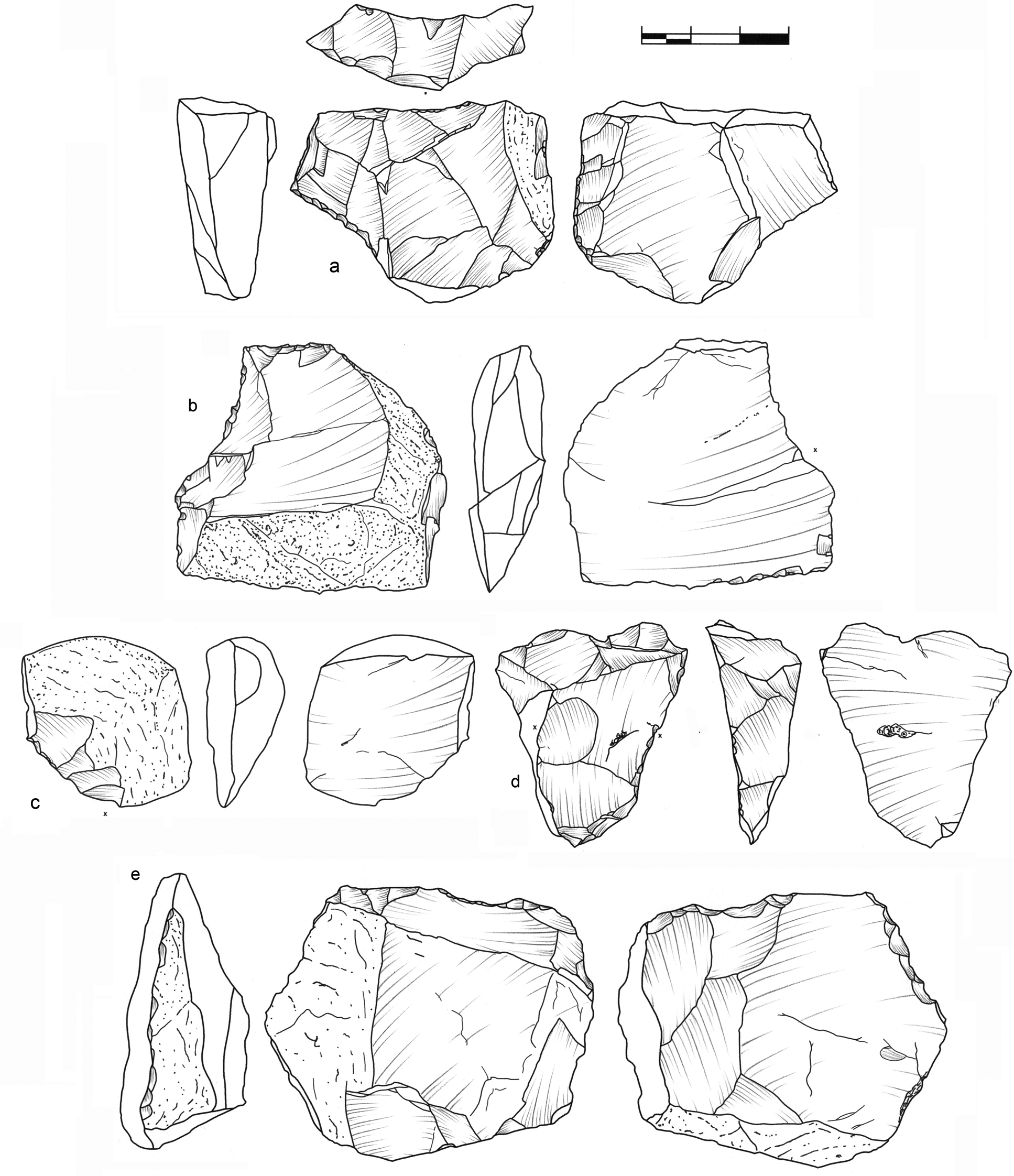

Supplement: S20 Fig — Quartzite (a, c-e) and rhyolite (b). a (art. no. 25), b (art. no. 125), c (art. no. 162), d (art. no. 489), e (art. no. 150). (TIF) [file pone.0248279.s021.tif]

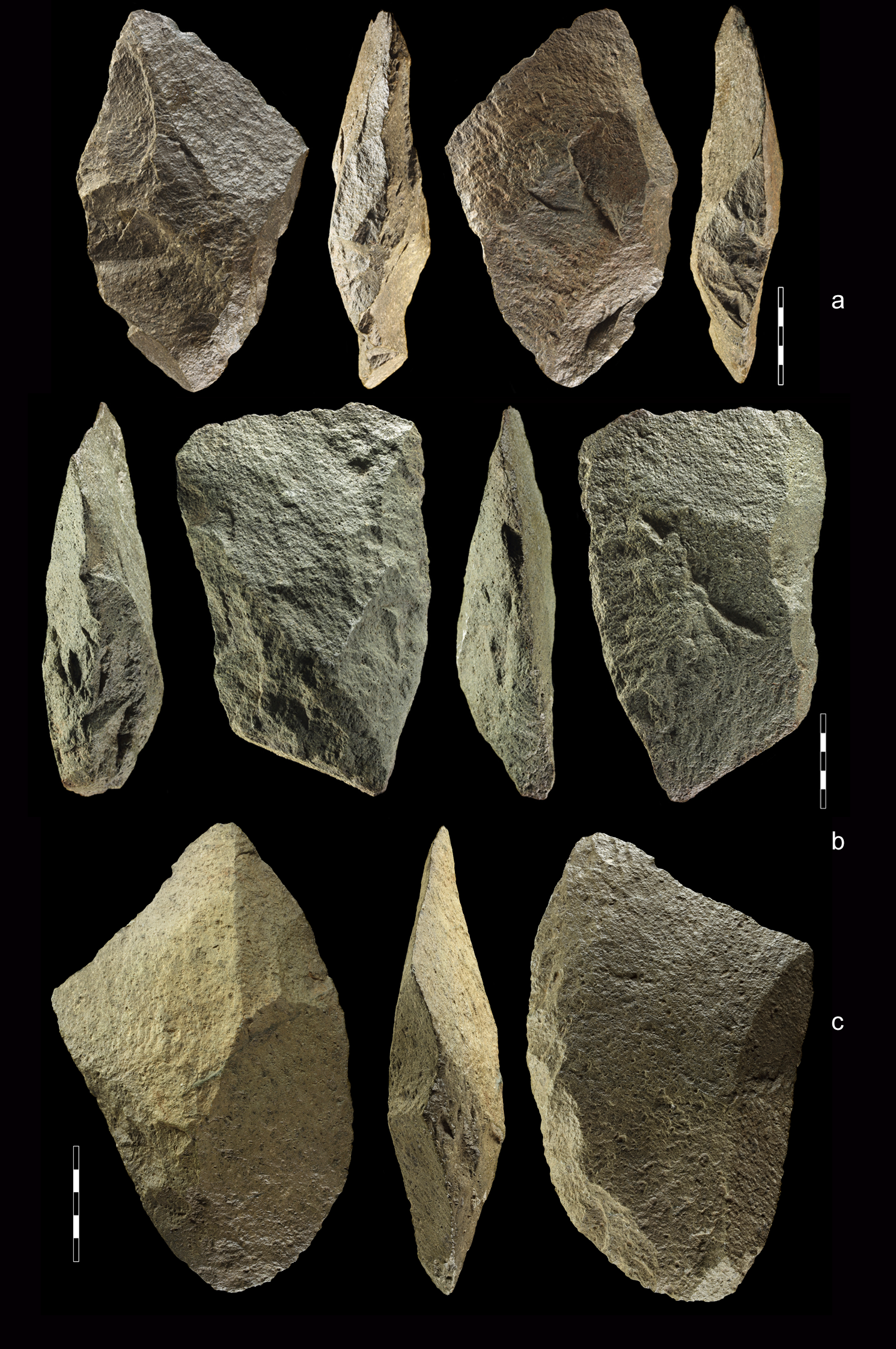

Supplement: S21 Fig — Cleavers made on large flakes. a: rhyolite cleaver on Kombewa flake from the surface of the site; b: trench I/2017—rhyolite cleaver on Kombewa flake; c: trench I/2017—rhyolite cleaver made on flake. (TIF) [file pone.0248279.s022.tif]
