# Supplementary material for: The oldest Homo erectus buried lithic horizon from the Eastern Saharan Africa. EDAR 7 - an Acheulean assemblage with Kombewa method from the Eastern Desert, Sudan
Source: PLoS One. 2021 Mar 23;16(3):e0248279. doi: 10.1371/journal.pone.0248279 (PMC7989774; doi:10.1371/journal.pone.0248279)
Supplement: S1 Table — (DOCX) [file pone.0248279.s023.docx]

**S1 Table. The Single-Aliquot Regenerative-dose procedure used in this study.**

| **Regeneration Step** | **Quartz single-aliquot** |
| --- | --- |
| 1 | Irradiation (regeneration only) |
| 2 | Preheat 220°C or 260°C (ramp 5°C s-1, hold 10 s) |
| 3 | IRSL 50 ⁰C, 100 s stimulation |
| 4 | Blue laser stimulation at 125°C, 200 s stimulation |
| 5 | Irradiation (test dose) |
| 6 | Preheat 160°C or 220°C (ramp 5°C s-1, hold 10 s) |
| 7 | IRSL 50 ⁰C, 100 s stimulation |
| 8 | Blue laser stimulation at 125°C, 200 s stimulation |
| 9 | Return to step 1 |
