# Supplementary material for: The oldest Homo erectus buried lithic horizon from the Eastern Saharan Africa. EDAR 7 - an Acheulean assemblage with Kombewa method from the Eastern Desert, Sudan
Source: PLoS One. 2021 Mar 23;16(3):e0248279. doi: 10.1371/journal.pone.0248279 (PMC7989774; doi:10.1371/journal.pone.0248279)
Supplement: S2 Table — (DOCX) [file pone.0248279.s024.docx]

**S2 Table. Frequencies of state of preservation of EDAR 7 inventory (chips and debris excluded).**

| **State**  **of preservation** | **Metamorphic** | | | | **Sedimentary** | | | | **Igneous** | | **Total** | |
| --- | --- | --- | --- | --- | --- | --- | --- | --- | --- | --- | --- | --- |
|  | **Quartzite** | | **Quartzitic sandstone** | | **Chert** | | **Hudi Chert** | | **Rhyolite** | |  |  |
|  | **n** | **%** | **n** | **%** | **n** | **%** | **n** | **%** | **n** | **%** | **n** | **%** |
| **fresh** | 1 | 0,3 | - | - | - | - | - | - | 1 | 1,6 | 2 | 0,4 |
| **slightly abraded** | 203 | 49,9 | - | - | 1 | 100 | 1 | 100 | 19 | 30,6 | 224 | 47,4 |
| **abraded** | 203 | 49,8 | 1 | 100 | - | - | - | - | 42 | 67,8 | 246 | 52,2 |
| **Total** | 407 | 100 | 1 | 100 | 1 | 100 | 1 | 100 | 62 | 100 | 472 | 100 |
