# Supplementary material for: The oldest Homo erectus buried lithic horizon from the Eastern Saharan Africa. EDAR 7 - an Acheulean assemblage with Kombewa method from the Eastern Desert, Sudan
Source: PLoS One. 2021 Mar 23;16(3):e0248279. doi: 10.1371/journal.pone.0248279 (PMC7989774; doi:10.1371/journal.pone.0248279)
Supplement: S3 Table — (DOCX) [file pone.0248279.s025.docx]

**S3 Table. Core types from EDAR 7 with their frequencies according to raw materials.**

| **Core type** | **Quartzite** | | **Rhyolite** | | **Total** | |
| --- | --- | --- | --- | --- | --- | --- |
|  | **n** | **%** | **n** | **%** | **n** | **%** |
| **Amorphic** | 19 | 27,54 | - | - | 19 | 27,54 |
| **Bidirectional** | 1 | 1,45 | - | - | 1 | 1,45 |
| **Unidirectional** | 21 | 30,43 | - | - | 21 | 30,43 |
| **Discoidal** | 4 | 5,80 | - | - | 4 | 5,80 |
| **Ninety-degree** | 1 | 1,45 | - | - | 1 | 1,45 |
| **Giant** | 2 | 2,90 | - | - | 2 | 2,90 |
| **Initially struck** | 8 | 11,59 | 2 | 2,90 | 10 | 14,49 |
| **Patterned, multiple platform** | 1 | 1,45 | - | - | 1 | 1,45 |
| **Unpatterned, multiple platform** | 9 | 13,04 | - | - | 9 | 13,04 |
| **Unclassifiable or fragmentary** | 1 | 1,45 | - | - | 1 | 1,45 |
| **Total** | 67 | 97,10 | 2 | 2,90 | **69** | **100,00** |
