# Supplementary material for: The oldest Homo erectus buried lithic horizon from the Eastern Saharan Africa. EDAR 7 - an Acheulean assemblage with Kombewa method from the Eastern Desert, Sudan
Source: PLoS One. 2021 Mar 23;16(3):e0248279. doi: 10.1371/journal.pone.0248279 (PMC7989774; doi:10.1371/journal.pone.0248279)
Supplement: S4 Table — (DOCX) [file pone.0248279.s026.docx]

**S4 Table. Exploitation stage of cores.**

| **Stage** | **n** | **%** |
| --- | --- | --- |
| **Tested nodule** | 4 | 5,80 |
| **Precore** | 14 | 20,29 |
| **Advanced exploitation** | 50 | 72,46 |
| **Unidentifiable** | 1 | 1,45 |
| **Total** | 69 | 100 |
