# Supplementary material for: The oldest Homo erectus buried lithic horizon from the Eastern Saharan Africa. EDAR 7 - an Acheulean assemblage with Kombewa method from the Eastern Desert, Sudan
Source: PLoS One. 2021 Mar 23;16(3):e0248279. doi: 10.1371/journal.pone.0248279 (PMC7989774; doi:10.1371/journal.pone.0248279)
Supplement: S5 Table — (DOCX) [file pone.0248279.s027.docx]

**S5 Table. Dimensions (mm) and weight (g) of complete cores (n=68).**

| **Dimension** | **Max** | **Min** | **Mean** | **Median** | **St. Dev.** |
| --- | --- | --- | --- | --- | --- |
| **Length** | 340 | 17,8 | 71,99 | 62 | 46,13 |
| **Width** | 412 | 11,5 | 111 | 62 | 53,78 |
| **Thickness** | 190 | 15,6 | 61,93 | 55,4 | 35,57 |
| **Weight** | 9250 | 11,5 | 713,41 | 287 | 1412,63 |
