# Supplementary material for: The oldest Homo erectus buried lithic horizon from the Eastern Saharan Africa. EDAR 7 - an Acheulean assemblage with Kombewa method from the Eastern Desert, Sudan
Source: PLoS One. 2021 Mar 23;16(3):e0248279. doi: 10.1371/journal.pone.0248279 (PMC7989774; doi:10.1371/journal.pone.0248279)
Supplement: S6 Table — (DOCX) [file pone.0248279.s028.docx]

**S6 Table. Dimensions (mm) and weight (g) of complete flakes (n=197).**

| **Dimension** | **Min** | **Max** | **Mean** | **Median** | **St. Dev.** |
| --- | --- | --- | --- | --- | --- |
| **Length** | 16,4 | 171 | 49,64 | 41,7 | 27,46 |
| **Width** | 12,8 | 165 | 46,52 | 37,5 | 27,84 |
| **Thickness** | 1,6 | 81,5 | 20,78 | 16,7 | 14,02 |
| **Weight** | 2,2 | 1611 | 107,93 | 27 | 220,20 |
