# Supplementary material for: The oldest Homo erectus buried lithic horizon from the Eastern Saharan Africa. EDAR 7 - an Acheulean assemblage with Kombewa method from the Eastern Desert, Sudan
Source: PLoS One. 2021 Mar 23;16(3):e0248279. doi: 10.1371/journal.pone.0248279 (PMC7989774; doi:10.1371/journal.pone.0248279)
Supplement: S7 Table — (DOCX) [file pone.0248279.s029.docx]

**S7 Table. Dorsal face blow directions of flakes.**

| **Blank type** | **n** | **%** |
| --- | --- | --- |
| **Unidirectional** | 72 | 28,1 |
| **Bidirectional** | 36 | 14,2 |
| **Multidirectional** | 71 | 27,7 |
| **Indeterminate** | 77 | 30 |
| **Total** | 256 | 100,0 |
