# Supplementary material for: The oldest Homo erectus buried lithic horizon from the Eastern Saharan Africa. EDAR 7 - an Acheulean assemblage with Kombewa method from the Eastern Desert, Sudan
Source: PLoS One. 2021 Mar 23;16(3):e0248279. doi: 10.1371/journal.pone.0248279 (PMC7989774; doi:10.1371/journal.pone.0248279)
Supplement: S8 Table — (DOCX) [file pone.0248279.s030.docx]

**S8 Table. Flake butt types**

| **Butt type** | **n** | **%** |
| --- | --- | --- |
| **Cortex/raw** | 53 | 20,70 |
| **Dihedral** | 4 | 1,56 |
| **Faceted** | 7 | 2,73 |
| **Linear** | 27 | 10,55 |
| **Plain** | 115 | 44,92 |
| **Punctiform** | 22 | 8,59 |
| **Unidetifiable** | 28 | 10,94 |
| **Total** | 256 | 100,0 |
