# Supplementary material for: The oldest Homo erectus buried lithic horizon from the Eastern Saharan Africa. EDAR 7 - an Acheulean assemblage with Kombewa method from the Eastern Desert, Sudan
Source: PLoS One. 2021 Mar 23;16(3):e0248279. doi: 10.1371/journal.pone.0248279 (PMC7989774; doi:10.1371/journal.pone.0248279)
Supplement: S9 Table — (DOCX) [file pone.0248279.s031.docx]

**S9 Table. Large flakes from EDAR 7 (mm and g).**

| **Art No** | **Blank kind** | **Nat.**  **surface (%)** | **Blank type** | **Butt type** | **Length max** | **Width max** | **Thickness max** | **Butt**  **width** | **Butt length** | **Weight** |
| --- | --- | --- | --- | --- | --- | --- | --- | --- | --- | --- |
| 164 | Flake | 0-25 | multidirectional | cortex | 127,4 | 117,7 | 81,5 | 91,1 | 72,1 | 1611 |
| 207 | Flake | 0-25 | multidirectional | plain | 110,3 | 102,6 | 71,2 | 47 | 21,4 | 715 |
| 517 | Cortex flake | 100 | - | cortex | 171,0 | 121,9 | 60,5 | 74,1 | 42,3 | 1358 |
