# Supplementary material for: The oldest Homo erectus buried lithic horizon from the Eastern Saharan Africa. EDAR 7 - an Acheulean assemblage with Kombewa method from the Eastern Desert, Sudan
Source: PLoS One. 2021 Mar 23;16(3):e0248279. doi: 10.1371/journal.pone.0248279 (PMC7989774; doi:10.1371/journal.pone.0248279)
Supplement: S10 Table — (DOCX) [file pone.0248279.s032.docx]

**S10 Table. Blank type and preservation state of bifaces.**

| **Type of blank** | **n** | **%** |
| --- | --- | --- |
| **Hand-axe on flake** | 1 | 2,70 |
| **Hand-axe on possibly flake** | 4 | 10,81 |
| **Hand-axe on chunk** | 9 | 24,32 |
| **Hand-axe on pebble** | 11 | 29,73 |
| **Hand-axe on Kombewa flake** | 1 | 2,70 |
| **Cleaver on flake** | 2 | 5,41 |
| **Cleaver on possibly flake** | 3 | 8,11 |
| **Cleaver on chunk** | 1 | 2,70 |
| **Cleaver on pebble** | 3 | 8,11 |
| **Undetermined** | 2 | 5,41 |
| **Total** | 37 | 100 |
| **Preservation state** | n | % |
| **Complete** | 31 | 83,78 |
| **Broken in lateral part** | 2 | 5,41 |
| **Broken in distal part** | 1 | 2,70 |
| **Broken in poximal part** | 2 | 5,41 |
| **Fragment** | 1 | 2,70 |
| **Total** | 37 | 100 |
