# Supplementary material for: The oldest Homo erectus buried lithic horizon from the Eastern Saharan Africa. EDAR 7 - an Acheulean assemblage with Kombewa method from the Eastern Desert, Sudan
Source: PLoS One. 2021 Mar 23;16(3):e0248279. doi: 10.1371/journal.pone.0248279 (PMC7989774; doi:10.1371/journal.pone.0248279)
Supplement: S11 Table — (DOCX) [file pone.0248279.s033.docx]

**S11 Table. Dimensions of LCT (mm).**

| **LCT (without bifacial preform)** | | | | | | |
| --- | --- | --- | --- | --- | --- | --- |
|  | **n** | **Min** | **Max** | **Mean** | **Median** | **St. Deviation** |
| **Max. Length** | 35 | 68,2 | 173 | 122,75 | 119,3 | 23,50 |
| **Max. Width** | 35 | 51 | 179 | 93,7 | 88,2 | 27,29 |
| **Max. Thickness** | 35 | 19,1 | 78 | 46,22 | 42,6 | 13,81 |
| **Max. Weight** | 35 | 178 | 1677 | 657,91 | 483 | 392,40 |
| **Cleavers** | | | | | | |
| **Max. Length** | 9 | 90,7 | 164 | 123,13 | 113,3 | 23,7 |
| **Max. Width** | 9 | 65,5 | 111,6 | 80,42 | 76,9 | 14,5 |
| **Max. Thickness** | 9 | 19,1 | 62,5 | 37,93 | 34 | 12,8 |
| **Max. Weight** | 9 | 178 | 955 | 482,56 | 459 | 280,7 |
| **Hand-axes** | | | | | | |
| **Max. Length** | 14 | 100,7 | 173 | 127,41 | 123,1 | 22,15 |
| **Max. Width** | 14 | 51 | 127,3 | 85,94 | 85,4 | 19,37 |
| **Max. Thickness** | 14 | 34,4 | 61,5 | 47,16 | 42,9 | 9,65 |
| **Max. Weight** | 14 | 269 | 1496 | 619,86 | 510,5 | 327,68 |
| **Hand-axes with "cleaver like" edge** | | | | | | |
| **Max. Length** | 4 | 105,9 | 159 | 127,8 | 123,15 | 127,8 |
| **Max. Width** | 4 | 68,6 | 98,6 | 82,15 | 80,7 | 82,15 |
| **Max. Thickness** | 4 | 39,9 | 60,2 | 48,45 | 46,85 | 48,45 |
| **Max. Weight** | 4 | 371 | 1052 | 585,25 | 459 | 585,25 |
| **Choppers and chopping tools** | | | | | | |
| **Max. Length** | 8 | 68,2 | 147,6 | 111,61 | 116,2 | 25,26 |
| **Max. Width** | 8 | 84,8 | 179 | 128,00 | 131,8 | 29,30 |
| **Max. Thickness** | 8 | 26,2 | 78 | 52,78 | 57,25 | 19,43 |
| **Max. Weight** | 8 | 288 | 1677 | 958,13 | 1112,5 | 512,96 |
