# Supplementary material for: The oldest Homo erectus buried lithic horizon from the Eastern Saharan Africa. EDAR 7 - an Acheulean assemblage with Kombewa method from the Eastern Desert, Sudan
Source: PLoS One. 2021 Mar 23;16(3):e0248279. doi: 10.1371/journal.pone.0248279 (PMC7989774; doi:10.1371/journal.pone.0248279)
Supplement: S12 Table — Dimensions of complete flake tools (n = 84) (mm and g). (DOCX) [file pone.0248279.s034.docx]

**S12 Table. Retouched flake tools.** Dimensions of complete flake tools (n=84) (mm and g).

|  | **Max** | **Min** | **Mean** | **Median** | **St. Deviation** |
| --- | --- | --- | --- | --- | --- |
| **Length** | 189 | 21,4 | 54,51 | 45,8 | 36,01 |
| **Width** | 179 | 9,8 | 49,16 | 41,05 | 34,61 |
| **Thickness** | 78 | 5,3 | 20,21 | 16,1 | 15,40 |
| **Weight** | 1677 | 2,3 | 182,07 | 40,05 | 353,36 |
