# Supplementary material for: The oldest Homo erectus buried lithic horizon from the Eastern Saharan Africa. EDAR 7 - an Acheulean assemblage with Kombewa method from the Eastern Desert, Sudan
Source: PLoS One. 2021 Mar 23;16(3):e0248279. doi: 10.1371/journal.pone.0248279 (PMC7989774; doi:10.1371/journal.pone.0248279)
Supplement: S13 Table — (DOCX) [file pone.0248279.s035.docx]

**S13 Table.** **Edge modification and delineation type, location of retouch; retouched edge length (complete flake tools only).**

| **Edge modification type** | **n** | **%** | **Retouch location** | **n** | **%** |
| --- | --- | --- | --- | --- | --- |
| **Bifacial** | 2 | 2,38 | **Right edge** | 26 | 30,95 |
| **Endscraper type**  **(short, abrupt and oblique)** | 8 | 8,33 | **Left edge** | 15 | 17,86 |
| **Invasive** | 1 | 1,19 | **Both edges** | 9 | 10,71 |
| **Irregular** | 2 | 2,38 | **Distal** | 26 | 30,95 |
| **Mixed (on the same edge)** | 1 | 1,19 | **Distal and left edge** | 1 | 1,19 |
| **Notch/denticulate type**  **(short, concave and low)** | 40 | 48,81 | **Distal and right edge** | 2 | 2,38 |
| **Regular** | 11 | 13,10 | **Distal and both edges** | 4 | 4,76 |
| **Sidescraper type**  **(invasive, semi-abrupt and parallel)** | 19 | 22,62 | **Proximal** | 1 | 1,19 |
| **Total** | 84 | 100 | **Total** | 84 | 100 |
| **Edge delineation type** | **n** | **%** | **Face of retouch** | **n** | **%** |
| **Concave** | 22 | 26,19 | **Ventral** | 23 | 27,38 |
| **Convex** | 7 | 8,33 | **Dorsal** | 48 | 57,14 |
| **Denticulated** | 20 | 23,81 | **Ventral and dorsal** | 13 | 15,48 |
| **Oblique** | 1 | 1,19 | **Total** | 84 | 100 |
| **Awl-like** | 5 | 5,95 |  |  |  |
| **Straight** | 29 | 34,52 |  |  |  |
| **Total** | 84 | 100 |  |  |  |
| **Length of retouched edge (mm)** | | | | | |
| **n** | **Max** | **Min** | **Mean** | **Median** | **St. Dev.** |
| **84** | 140 | 3 | 40,04 | 34,65 | 30,94 |
