# Supplementary material for: The oldest Homo erectus buried lithic horizon from the Eastern Saharan Africa. EDAR 7 - an Acheulean assemblage with Kombewa method from the Eastern Desert, Sudan
Source: PLoS One. 2021 Mar 23;16(3):e0248279. doi: 10.1371/journal.pone.0248279 (PMC7989774; doi:10.1371/journal.pone.0248279)
Supplement: S14 Table — (DOCX) [file pone.0248279.s036.docx]

**S14 Table. Percentage value of variance of selected main principal components.**

| **PC** | **Eigenvalue** | **% variance** |
| --- | --- | --- |
| **1** | 0.00776193 | 46.801 |
| **2** | 0.00485629 | 29.281 |
| **3** | 0.00118757 | 7.1605 |
| **4** | 0.000774067 | 4.6673 |
| **5** | 0.000355846 | 2.1456 |
| **6** | 0.000314931 | 1.8989 |
| **7** | 0.000250042 | 1.5076 |
| **8** | 0.000179724 | 1.0837 |
| **9** | 0.000153455 | 0.92526 |
| **10** | 0.000101203 | 0.61021 |
