# Supplementary material for: The oldest Homo erectus buried lithic horizon from the Eastern Saharan Africa. EDAR 7 - an Acheulean assemblage with Kombewa method from the Eastern Desert, Sudan
Source: PLoS One. 2021 Mar 23;16(3):e0248279. doi: 10.1371/journal.pone.0248279 (PMC7989774; doi:10.1371/journal.pone.0248279)
Supplement: S15 Table — (DOCX) [file pone.0248279.s037.docx]

**S15 Table. Results of the MANOVA and PERMANOVA tests.**

| **MANOVA test** | | **PERMANOVA test** | |
| --- | --- | --- | --- |
| **Pillai’s Trace:** | 2.33 | Permutation N: | 9999 |
| **df1:** | 200 | Total sum of squares: | 0.6039 |
| **df2:** | 236 | Within-group sum of squares: | 0.5006 |
| **F:** | 1.646 | F: | 5.42 |
| **p (<0,05):** | 0.0001198 | p (<0,05): | 0.0001 |
