# Supplementary material for: The oldest Homo erectus buried lithic horizon from the Eastern Saharan Africa. EDAR 7 - an Acheulean assemblage with Kombewa method from the Eastern Desert, Sudan
Source: PLoS One. 2021 Mar 23;16(3):e0248279. doi: 10.1371/journal.pone.0248279 (PMC7989774; doi:10.1371/journal.pone.0248279)
Supplement: S16 Table — KO–Kharga Oasis, site nr 10; BS- Bir Sahara site nr 14, DO–Dakhla Oasis site nr E-72-1. (DOCX) [file pone.0248279.s038.docx]

**S16 Table. Results of similarity in pairs implemented in the PERMANOVA test.** KO – Kharga Oasis, site nr 10; BS- Bir Sahara site nr 14, DO – Dakhla Oasis site nr E-72-1.

| **Site** | **ED 7** | **KO** | **BS** | **ED 133** | **DO** |
| --- | --- | --- | --- | --- | --- |
| **ED 7** | - | 0.0001 | 0.1621 | 0.0001 | 0.0001 |
| **KO** | 0.0001 | - | 0.0126 | 0.0699 | 0.5594 |
| **BS** | 0.1621 | 0.0126 | - | 0.0001 | 0.0118 |
| **ED 133** | 0.0001 | 0.0699 | 0.0001 | - | 0.0097 |
| **DO** | 0.0001 | 0.5594 | 0.0118 | 0.0097 | - |
